# Supplementary material for: Research inefficiencies in external validation studies of the Framingham Wilson coronary heart disease risk rule: A systematic review
Source: PLoS One. 2024 Sep 13;19(9):e0310321. doi: 10.1371/journal.pone.0310321 (PMC12140082; doi:10.1371/journal.pone.0310321)
Supplement: S1 Table — (DOCX) [file pone.0310321.s001.docx]

S1 Table. The definition of coronary heart disease (CHD) outcome provided by external validation studies included in the meta-analyses.

| Study (Publication date) | Outcome definition |
| --- | --- |
| Orford (2002 Jul) | - Angina pectoris: recurrent chest discomfort lasting up to 15 minutes related to exertion and relieved by rest or nitroglycerin. - Acute or old MI: EKG changes (pathologic Q waves), elevation of serum enzymes with chest discomfort consistent with MI, or autopsy. - CHD death: a death certificate with an underlying cause of death coded to 410-414. |
| Empana (2003 Nov) | - Angina pectoris, MI and coronary death. |
| Nawrot (2004 Apr) | - Cardiac events according to ICD-8: chronic ischaemic heart disease (4110, 4120, 4129, 4130), acute MI (ICD-9 code 4100), heart failure (4270, 4271, 4272, 4279, 4280, 4290), and sudden death (7950). |
| Ducloux (2004 Jul) | - Typical history of angina with abnormal coronarography. - Coronary revascularization, including coronary artery bypass surgery or percutaneous transluminal coronary angioplasty. - MI: serial 12-lead ECG evidence or Q-wave infarction and appropriate myocardial enzyme elevations. |
| Blake (2004 Aug) | - Nonfatal MI: clinical (chest pain with serial ECG changes or enzyme elevation) or silent (Minnesota codes 1.1 or 1.2 on resting ECG). - Cardiac death: death during the follow-up period due to acute MI, congestive heart failure, or sudden death not attributable to another cause |
| Jimeno Mollet (2005 Jan) | - Angina, acute MI, or abnormalities in non-invasive tests (such as EKG or Holter) |
| Mora (2005 Apr) | - Angina requiring revascularization, unstable/stable angina with a documented flow-limiting stenosis, MI, or sudden cardiac death. |
| Ferrario (2005 Apr) | - Non-fatal coronary events: hospital discharge diagnosis with ICD-9 410–411 codes for suspected acute infarction and ICD-9 CM 36.0-9 codes for coronary surgery revascularization. - Coronary deaths: underlying causes of death ICD-9 codes 410–414, 798, 799 as well as 250, 428, 440 codes in association with 410–414 codes. |
| Marrugat (2007 Jan) | - Angina pectoris: a positive ischaemia test or coronary angiography or characteristic ECG changes occurred during chest pain. - Unrecognised AMI: the follow-up ECG showed Q waves or ischaemia signs that were absent in the initial ECG examination and were confirmed by echocardiography and cardiac scintigraphy showing signs of a myocardial scar. - Non-fatal acute MI: a characteristic ECG, enzyme or troponin increase (not explained by other conditions), with suggestive symptoms, and the patient survived at least 28 days after symptom onset. - Fatal AMI: all AMI criteria were met and the patient died within 28 days of symptom onset, a diagnostic necropsy existed or sudden death occurred with suggestive symptoms that could not be explained by other diseases. |
| Miyasaka (2007 Apr) | - Angina pectoris with angiographic confirmation: atherosclerotic narrowing of least 50%. - Unstable angina: new or accelerated ischemic symptoms without elevation of cardiac enzymes. - Acute MI, (requires two or more of the followings ): compatible clinical presentation, diagnostic cardiac enzyme, and ECG changes consistent with MI. - Coronary death: death attributable to fatal acute MI and death with the primary cause being CHD |
| Nilsson (2007 May) | - Non-fatal MI (ICD-9 code 410), or death from ischaemic heart disease (ICD 410–414). |
| Cañón-Barroso (2007 Jul) | - Angina and fatal and nonfatal acute MI |
| Fowkes (2008 Jul) | - Major coronary events: nonfatal myocardial infarction, coronary death. - Coronary revascularization and angina were not included. |
| Becker (2008 Oct) | - Coronary revascularization: CABG or (PTCA) - MI was (requires at least two of the following): prolonged chest pain leading to hospital admission, specific ECG changes, elevation of serum creatine kinase levels up to twice the upper limit with an elevated creatine kinase-MB fraction or troponin level. - CAD death: death was proved to be due to coronary atherosclerosis by autopsy, occurred within 1 hour after onset of prolonged severe chest pain, or occurred during hospital admission because of MI. |
| Chang (2009 Nov) | - Coronary revascularization with by-pass surgery or percutaneous approaches were - An MI: clinical, ECG, and enzymatic criteria. - Cardiac death: death of any cardiac cause, including a fatal MI, sudden arrhythmic death, or heart failure. |
| Brunner (2010 May) | - Angina: based on doctor’s diagnosis or ECG. - Non-fatal MI - fatal CHD: underlying cause of ICD-9 code 410–414 |
| Lorenz (2010 Aug) | - Pectoral angina, myocardial infarction, or death |
| Sivapalaratnam (2010 Oct) | - A hospital admission or death caused by CHD event (unstable angina, stable angina and MI). |
| Erbel (2010 Oct) | - Nonfatal acute myocardial infarction and coronary death based on symptoms, signs of ECG, and creatine kinase, as well as troponin T or I, and necropsy. |
| Baena-Díez (2010 Nov) | - Angina: compatible history, with or without ECG changes during the episode, plus a positive exercise stress test, scintigraphy or coronary angiogram. - Nonfatal or fatal acute MI: hospital discharge report with markers of necrosis, autopsy report, or ICD-9 codes 410-414, 429.9 and 798 and ICD-10 codes I20-I25, I46.1 and R96 on the death certificate. |
| Rodondi (2012 Mar) | - Hospitalization for angina or revascularization (coronary angioplasty or surgery), nonfatal MI or coronary death. |
| Treeprasertsuk (2012 Jul) | - CHD event: congestive heart failure, unstable angina or MI, and/or a documented flow-limiting stenosis from angiography or angina requiring revascularization during follow-up. |
| Isaac (2013 Sep) | - A composite endpoint defined as occurrence of MI, heart failure, percutaneous transluminal coronary angioplasty, or coronary artery bypass graft. |
| Leistner (2014 Jun) | - Need for coronary revascularization by CABG or PCI, non-fatal MI, death from cardiovascular causes. |
| Silverman (2014 Sep) | - Probable angina followed by coronary revascularization and definite angina not followed by coronary revascularization: symptomatic event involving ischaemic chest, left arm, or jaw pain, though the symptoms may be atypical - MI: based on symptoms, ECG findings, and levels of cardiac biomarkers. - CHD death: occurred within 28 days after MI, chest pain within the 72 h before death or a history of CHD and there was no known non-atherosclerotic, non-cardiac cause of death. |
| Beaney (2015 Jul) | - Acute MI, silent MI, or undergoing coronary surgery. |
| Nadjiri (2016 Mar) | - Unstable angina pectoris, requiring hospitalization: typical acute chest pain with negative cardiac enzymes, if CAD could not be excluded as the cause of symptoms. - Non-fatal myocardial infarction: typical acute chest pain in rest lasting at least 20 min and at least one of the ECG criteria (ST-segment elevation in at least two limb leads of 0.1 millivolt (mV) or at least 0.2 mV in more than 2 precordial leads or positive cardiac enzymes). - All cause death. |
| Gerritsen (2016 Aug) | - Ischemic cardiac complication: MI, sudden death or fatal congestive heart failure. |
| Herrera (2016 Oct) | - Angina: compatible history, with or without ECG changes during the episode, plus a positive exercise stress test, scintigraphy or coronary arteriogram. - Nonfatal or fatal acute MI: hospital discharge report with markers of necrosis, autopsy report, or ICD-9 codes 410–414, 429.9, or 798, or ICD-10 codes I20-I25, I46.1 or R96 on the death certificate. |
| Iribarren (2016 Dec) | - Hospital primary discharge diagnoses of angina (stable or unstable), coronary revascularization procedures (coronary bypass or percutaneous intervention), MI or death because of CHD. |
| Forés (2018 Jan) | - Cardiac event: angina, MI, and coronary revascularization. |
| Calvo-Hueros (2021 Feb) | - Total coronary risk: angina, non-fatal and fatal MI. |
| Denes (2007 Mar) | - CHD: acute MI necessitating overnight hospitalization, silent MI identified on serial ECGs, or death due to CHD. |
| Mainous (2007 May) | - CHD: a myocardial infarction, cardiac procedure, or fatal CHD. |
| Malik (2011 Oct) | - Angina: clear documentation of chest pain or anginal equivalent and evidence of reversible myocardial ischemia or obstructive coronary artery disease or a positive stress test) - MI - Resuscitated cardiac arrest, or CHD death (MI within 28 days of death, resuscitated cardiac arrest, chest pain within the 72 h before death, or a history of CHD and the absence of a known nonatherosclerotic or noncardiac cause of death). |
| Gander (2014 Aug) | - CHD: self-report of MI or revascularization (including, bypass, coronary balloon, angioplasty, or stent) or death due to CHD. |
| Simmons (2008 Jun) | - CHD: the underlying cause of a hospital admission or death included stable angina, unstable angina, or MI: ICD-9 codes 410 to 414 or ICD-10 codes I22 to I25. |
| Barbier (2016n Jul) | - Major adverse cardiac events: a new diagnosis of angina pectoris, or symptom-driven coronary artery revascularization, non-fatal MI (i.e. a hospital diagnosis of MI set using the criteria defined by the Joint European Society of Cardiology/American College of Cardiology Committee), or cardiac death (i.e. cardiac arrest being registered as the primary cause of death in the death certificate). |
| Van Der Heijden (2009 Nov) | - CHD: nonfatal and fatal ischemic heart disease and sudden death (ICD-9 codes 410 – 414, 427.4, 427.5, and 798). |
| Merry (2012 Aug) | - CHD: unstable angina pectoris, a percutaneous transluminal coronary angioplasty, or coronary artery bypass grafting, an acute MI, or CHD as primary or secondary cause of death according to Statistics Netherlands (ICD9 410–414 or ICD10 I20–I25). |
| Koenig (2004 Mar) | - Coronary event: nonfatal or fatal MI, and sudden cardiac death. |
| Guckelberger (2006 Mar) | - Coronary event: development of coronary heart disease with typical clinical symptoms and confirmed by stress electrocardiogram or coronary angiography, MI confirmed by electrocardiogram or enzyme elevation. |
| Protopsaltis (2004 Jan) | - CAD: symptoms of CAD evaluated with a treadmill test and myocardium scanning, and established by coronary angiography. |

ICD: International Classification of Disease, MI: myocardial infarction, CHD: coronary heart disease, CAD: coronary artery disease, ECG: electrocardiogram, CABG: coronary artery bypass graft, PTCA: percutaneous transluminal coronary angioplasty, PCI: percutaneous coronary intervention
