# Supplementary material for: Research inefficiencies in external validation studies of the Framingham Wilson coronary heart disease risk rule: A systematic review
Source: PLoS One. 2024 Sep 13;19(9):e0310321. doi: 10.1371/journal.pone.0310321 (PMC12140082; doi:10.1371/journal.pone.0310321)
Supplement: S2 Table — (DOCX) [file pone.0310321.s002.docx]

S6 Table. List of excluded articles and categories for the exclusion.

| Article | Category |
| --- | --- |
| 1. Aarabi M, Jackson PR. Predicting coronary risk in UK south asians: An adjustment method for framingham-based tools. European Journal of Cardiovascular Prevention and Rehabilitation 2005;12(1):46-51. | B |
| 1. Abdullah SM, Defina LF, Leonard D, Barlow CE, Radford NB, Willis BL, et al. Long-Term association of low-density lipoprotein cholesterol with cardiovascular mortality in individuals at low 10-year risk of atherosclerotic cardiovascular disease: Results from the cooper center longitudinal study. Circulation 2018;138(21):2315-25. | A |
| 1. Abeles RD, Mullish BH, Forlano R, Kimhofer T, Adler M, Tzallas A, et al. Derivation and validation of a cardiovascular risk score for prediction of major acute cardiovascular events in non-alcoholic fatty liver disease; the importance of an elevated mean platelet volume. Alimentary Pharmacology and Therapeutics 2019. | C |
| 1. Acevedo M, Valentino G, Kramer V, Bustamante MJ, Adasme M, Orellana L, et al. Evaluation the american college of cardiology and american heart association predictive score for cardiovascular diseases. Revista Medica De Chile 2017;145(3):292-8. | C |
| 1. Acharjee S, Qin J, Murphy SA, McCabe C, Cannon CP. Distribution of traditional and novel risk factors and their relation to subsequent cardiovascular events in patients with acute coronary syndromes (from the PROVE IT-TIMI 22 trial). American Journal of Cardiology 2010;105(5):619-23. | A |
| 1. Achterberg S, Kappelle LJ, De Bakker PIW, Traylor M, Algra A, Van Der Graaf Y, et al. No additional prognostic value of genetic information in the prediction of vascular events after cerebral ischemia of arterial origin: The promise study. PLoS ONE 2015;10(4). | A |
| 1. Aggarwal A, Atreja A, Kapadia S, Lopez R, Achkar JP. Conventional risk factors and cardiovascular outcomes of patients with inflammatory bowel disease with confirmed coronary artery disease. Inflammatory Bowel Diseases 2014;20(9):1593-601. | D |
| 1. Ajnakina O, Agbedjro D, McCammon R, Faul J, Murray RM, Stahl D, Steptoe A. Development and validation of prediction model to estimate 10-year risk of all-cause mortality using modern statistical learning methods: A large population-based cohort study and external validation. BMC Medical Research Methodology 2021;21(1). | A |
| 1. Aktas MK, Ozduran V, Pothier CE, Lang R, Lauer MS. Global risk scores and exercise testing for predicting all-cause mortality in a preventive medicine program. JAMA - Journal of the American Medical Association 2004;292(12):1462-8. | B |
| 1. Al-Lawati JA, Barakat MN, Al-Lawati NA, Al-Maskari MY, Elsayed MK, Mikhailidis DP, Al-Zakwani IS. Cardiovascular risk assessment in diabetes mellitus: Comparison of the general framingham risk profile versus the world health organization/ international society of hypertension risk prediction charts in arabs - clinical implications. Angiology 2013;64(5):336-42. | B |
| 1. Al-Shamsi S. Performance of the framingham coronary heart disease risk score for predicting 10-year cardiac risk in adult united arab emirates nationals without diabetes: A retrospective cohort study. BMC Family Practice 2020;21(1). | B |
| 1. Alzamora MT, Baena-Díez JM, Sorribes M, Forés R, Toran P, Vicheto M, et al. Peripheral arterial disease study (PERART): Prevalence and predictive values of asymptomatic peripheral arterial occlusive disease related to cardiovascular morbidity and mortality. BMC Public Health 2007;7. | A |
| 1. Amer MS, Khater MS, Omar OH, Mabrouk RA, El-Kawaly WH. Framingham risk score and ankle- brachial index in diabetic older adults. International Journal of Cardiology 2013;168(2):1620-1. | C |
| 1. Ang TFA, An N, Ding H, Devine S, Auerbach SH, Massaro J, et al. Using data science to diagnose and characterize heterogeneity of alzheimer's disease. Alzheimer's and Dementia: Translational Research and Clinical Interventions 2019;5:264-71. | A |
| 1. Aranceta J, Pérez Rodrigo C, Foz Sala M, Mantilla T, Serra Majem L, Moreno B, et al. Tables of coronary risk evaluation adapted to the spanish population: The DORICA study. Medicina Clinica 2004;123(18):686-91. | A |
| 1. Araujo AB, Hall SA, Ganz P, Chiu GR, Rosen RC, Kupelian V, et al. Does erectile dysfunction contribute to cardiovascular disease risk prediction beyond the framingham risk score? Journal of the American College of Cardiology 2010;55(4):350-6. | C |
| 1. Armenian SH, Yang D, Teh JB, Atencio LC, Gonzales A, Wong FL, et al. Prediction of cardiovascular disease among hematopoietic cell transplantation survivors. Blood Advances 2018;2(14):1756-64. | A |
| 1. Aronis KN, Moreno M, Polyzos SA, Moreno-Navarrete JM, Ricart W, Delgado E, et al. Circulating irisin levels and coronary heart disease: Association with future acute coronary syndrome and major adverse cardiovascular events. International Journal of Obesity 2015;39(1):156-61. | A |
| 1. Arsenault BJ, Lemieux I, Després JP, Wareham NJ, Luben R, Kastelein JJP, et al. Cholesterol levels in small LDL particles predict the risk of coronary heart disease in the epic-norfolk prospective population study. European Heart Journal 2007;28(22):2770-7. | A |
| 1. Arsenault BJ, Rana JS, Lemieux I, Després JP, Wareham NJ, Kastelein JJP, et al. Physical activity, the framingham risk score and risk of coronary heart disease in men and women of the epic-norfolk study. Atherosclerosis 2010;209(1):261-5. | A |
| 1. Artigao-Rodenas LM, Carbayo-Herencia JA, Divisón-Garrote JA, Gil-Guillén VF, Massó-Orozco J, Simarro-Rueda M, et al. Framingham risk score for prediction of cardiovascular diseases: A population-based study from southern europe. PLoS ONE 2013;8(9). | A |
| 1. Arts EEA, Popa C, Den Broeder AA, Semb AG, Toms T, Kitas GD, et al. Performance of four current risk algorithms in predicting cardiovascular events in patients with early rheumatoid arthritis. Annals of the Rheumatic Diseases 2015;74(4):668-74. | B |
| 1. Aslibekyan S, Campos H, Loucks EB, Linkletter CD, Ordovas JM, Baylin A. Development of a cardiovascular risk score for use in low- and middle-income countries. Journal of Nutrition 2011;141(7):1375-80. | A |
| 1. Asselbergs FW, Hillege HL, Van Gilst WH. Framingham score and microalbuminuria: Combined future targets for primary prevention? Kidney International, Supplement 2004;66(92):S111-4. | D |
| 1. Assmann G, Cullen P, Schulte H. Simple scoring scheme for calculating the risk of acute coronary events based on the 10-year follow-up of the prospective cardiovascular münster (PROCAM) study. Circulation 2002;105(3):310-5. | B |
| 1. Bachir Cherif A, Temmar M, Labat C, Atif L, Chibane A, Benkhedda S, et al. Cardiovascular morbimortality after a follow-up of six years in black hypertensive in south algeria. Annales De Cardiologie Et D'Angeiologie 2014;63(3):168-75. | A |
| 1. Bachmann JM, Willis BL, Ayers CR, Khera A, Berry JD. Association between family history and coronary heart disease death across long-term follow-up in men: The cooper center longitudinal study. Circulation 2012;125(25):3092-8. | A |
| 1. Bacon SL, Lavoie KL, Arsenault A, Dupuis J, Pilote L, Laurin C, et al. The research on endothelial function in women and men at risk for cardiovascular disease (REWARD) study: Methodology. BMC Cardiovascular Disorders 2011;11. | A |
| 1. Baena-Díez JM, Alzamora MT, Forés R, Pera G, Torán P, Sorribes M. Ankle-brachial index improves the classification of cardiovascular risk: PERART/ARTPER study. Revista Espanola De Cardiologia 2011;64(3):186-92. | A |
| 1. Baena-Díez JM, Bermúdez-Chillida N, García-Lareo M, Byram AO, Vidal-Solsona M, Vilató-García M, et al. Role of pulse pressure, systolic blood pressure, and diastolic blood pressure in the prediction of cardiovascular risk. Cohort study. Medicina Clinica 2008;130(10):361-5. | A |
| 1. Baena-Díez JM, Subirana I, Ramos R, Gómez de la Cámara A, Elosua R, Vila J, et al. Validity assessment of low-risk SCORE function and SCORE function calibrated to the spanish population in the FRESCO cohorts. Revista Espanola De Cardiologia 2018;71(4):274-82. | A |
| 1. Balady GJ, Larson MG, Vasan RS, Leip EP, O'Donnell CJ, Levy D. Usefulness of exercise testing in the prediction of coronary disease risk among asymptomatic persons as a function of the framingham risk score. Circulation 2004;110(14):1920-5. | D |
| 1. Baldassarre D, Amato M, Pustina L, Castelnuovo S, Sanvito S, Gerosa L, et al. Measurement of carotid artery intima-media thickness in dyslipidemic patients increases the power of traditional risk factors to predict cardiovascular events. Atherosclerosis 2007;191(2):403-8. | A |
| 1. Balkau B. Prediction of the risk of cardiovascular mortality using a score that includes glucose as a risk factor. The DECODE study. Diabetologia 2004;47(12):2118-28. | A |
| 1. Bangalore S, Messerli FH, Ou FS, Tamis-Holland J, Palazzo A, Roe MT, et al. Blood pressure paradox in patients with non-st-segment elevation acute coronary syndromes. Results from 139,194 patients in the can rapid risk stratification of unstable angina patients suppress adverse outcomes with early implementation of the american college of cardiology/american heart association guidelines (CRUSADE) quality improvement initiative. American Heart Journal 2009;157(3):525-31. | A |
| 1. Bansal M, Kasliwal RR, Trehan N. Comparative accuracy of different risk scores in assessing cardiovascular risk in indians: A study in patients with first myocardial infarction. Indian Heart Journal 2014;66(6):580-6. | A |
| 1. Bansal M, Ranjan S, Kasliwal RR. Cardiovascular risk calculators and their applicability to south asians. Current Diabetes Reviews 2021;17(9). | A |
| 1. Bansal S, Wackers FJT, Inzucchi SE, Chyun DA, Davey JA, Staib LH, Young LH. Five-year outcomes in high-risk participants in the detection of ischemia in asymptomatic diabetics (DIAD) study: A post hoc analysis. Diabetes Care 2011;34(1):204-9. | D |
| 1. Bargnoux AS, Morena M, Jaussent I, Maurice F, Chalabi L, Leray-Moragues H, et al. A combined index of cardiac biomarkers as a risk factor for early cardiovascular mortality in hemodialysis patients. Clinical Chemistry and Laboratory Medicine 2013;51(9):1865-74. | C |
| 1. Barón AM, García-Peña A, García PK, Salazar E, Blanco CA, Betancur MC, Amaya NF. Risk factors associated with cardiovascular events in kidney transplant patients. Revista Colombiana De Cardiologia 2018;25(1):13-8. | A |
| 1. Barreto SM, Azeredo Passos VM, Aquino Cardoso AR, Lima-Costa MF. Quantifying the risk of coronary artery disease in a community. The bambuí project. Arquivos Brasileiros De Cardiologia 2003;81(6):549-61. | A |
| 1. Barrett-Connor E, Bergstrom J, Wright CM, Kramer CK. Heart disease risk factors in midlife predict subclinical coronary atherosclerosis more than 25 years later in survivors without clinical heart disease: The rancho bernardo study. Journal of the American Geriatrics Society 2009;57(6):1041-4. | A |
| 1. Barzi F, Patel A, Gu D, Sritara P, Lam TH, Rodgers A, Woodward M. Cardiovascular risk prediction tools for populations in asia. Journal of Epidemiology and Community Health 2007;61(2):115-21. | A |
| 1. Bates RE, Omer M, Abdelmoneim SS, Arruda-Olson AM, Scott CG, Bailey KR, et al. Impact of stress testing for coronary artery disease screening in asymptomatic patients with diabetes mellitus: A community-based study in olmsted county, minnesota. Mayo Clinic Proceedings 2016;91(11):1535-44. | C |
| 1. Bendzala M, Sabaka P, Caprnda M, Komornikova A, Bisahova M, Baneszova R, et al. Atherogenic index of plasma is positively associated with the risk of all-cause death in elderly women: A 10-year follow-up. Wiener Klinische Wochenschrift 2017;129(21-22):793-8. | A |
| 1. Benguzzi M, Mansell H, Hassan A, Elmoselhi H, Mainra R, Shoker A. Contribution of impaired renal function to cardiovascular risk prediction models in renal transplant recipients. Clinical Transplantation 2014;28(12):1383-92. | B |
| 1. Benn M, Nordestgaard BG, Jensen GB, Tybjærg-Hansen A. Improving prediction of ischemic cardiovascular disease in the general population using apolipoprotein B: The copenhagen city heart study. Arteriosclerosis, Thrombosis, and Vascular Biology 2007;27(3):661-70. | A |
| 1. Bérard E, Bongard V, Arveiler D, Amouyel P, Wagner A, Dallongeville J, et al. Ten-year risk of all-cause mortality: Assessment of a risk prediction algorithm in a french general population. European Journal of Epidemiology 2011;26(5):359-68. | A |
| 1. Bérard E, Bongard V, Ruidavets JB, Amar J, Ferrières J. Pulse wave velocity, pulse pressure and number of carotid or femoral plaques improve prediction of cardiovascular death in a population at low risk. Journal of Human Hypertension 2013;27(9):529-34. | C |
| 1. Bérard E, Séguro F, Bongard V, Dallongeville J, Arveiler D, Amouyel P, et al. Predictive accuracy of the european society of cardiology score among french people. Journal of Cardiopulmonary Rehabilitation and Prevention 2016;36(1):38-48. | A |
| 1. Berry JD, Lloyd-Jones DM, Garside DB, Greenland P. Framingham risk score and prediction of coronary heart disease death in young men. American Heart Journal 2007;154(1):80-6. | C |
| 1. Biering-Sørensen T, Biering-Sørensen SR, Olsen FJ, Sengeløv M, Jørgensen PG, Mogelvang R, et al. Global longitudinal strain by echocardiography predicts long-term risk of cardiovascular morbidity and mortality in a low-risk general population: The copenhagen city heart study. Circulation: Cardiovascular Imaging 2017;10(3). | C |
| 1. Biering-Sørensen T, Mogelvang R, Schnohr P, Jensen JS. Cardiac time intervals measured by tissue doppler imaging m-mode: Association with hypertension, left ventricular geometry, and future ischemic cardiovascular diseases. Journal of the American Heart Association 2016;5(1). | A |
| 1. Blaha MJ, Hung RK, Dardari Z, Feldman DI, Whelton SP, Nasir K, et al. Age-dependent prognostic value of exercise capacity and derivation of fitne-aociated biologic age. Heart 2016;102(6):431-7. | A |
| 1. Blaha MJ, Yeboah J, Al Rifai M, Liu K, Kronmal R, Greenland P. Providing evidence for subclinical CVD in risk assessment. Global Heart 2016;11(3):275-85. | A |
| 1. Bliden KP, Chaudhary R, Navarese EP, Sharma T, Kaza H, Tantry US, Gurbel PA. Thrombogenicity and central pulse pressure to enhance prediction of ischemic event occurrence in patients with established coronary artery disease: The magma-ischemia score. Atherosclerosis 2018;268:55-62. | D |
| 1. Bodde MC, Hermans MPJ, Jukema JW, Schalij MJ, Lijfering WM, Rosendaal FR, et al. Apolipoproteins A1, B, and apob/apoa1 ratio are associated with first st-segment elevation myocardial infarction but not with recurrent events during long-term follow-up. Clinical Research in Cardiology 2019;108(5):520-38. | A |
| 1. Boden WE, Hartigan PM, Mancini J, Teo KK, Chaitman BR, Maron DJ, et al. Risk prediction tool for assessing the probability of death or myocardial infarction in patients with stable coronary artery disease. American Journal of Cardiology 2020;130:1-6. | A |
| 1. Boekholdt SM, Hack CE, Sandhu MS, Luben R, Bingham SA, Wareham NJ, et al. C-reactive protein levels and coronary artery disease incidence and mortality in apparently healthy men and women: The epic-norfolk prospective population study 1993-2003. Atherosclerosis 2006;187(2):415-22. | A |
| 1. Bogers RP, Bemelmans WJE, Hoogenveen RT, Boshuizen HC, Woodward M, Knekt P, et al. Association of overweight with increased risk of coronary heart disease partly independent of blood pressure and cholesterol levels: A meta-analysis of 21 cohort studies including more than 300 000 persons. Archives of Internal Medicine 2007;167(16):1720-8. | A |
| 1. Bolton JL, Stewart MCW, Wilson JF, Anderson N, Price JF. Improvement in prediction of coronary heart disease risk over conventional risk factors using snps identified in genome-wide association studies. PLoS ONE 2013;8(2). | A |
| 1. Bos S, Grobbee DE, Boer JMA, Verschuren WM, Beulens JWJ. Alcohol consumption and risk of cardiovascular disease among hypertensive women. European Journal of Cardiovascular Prevention and Rehabilitation 2010;17(1):119-26. | A |
| 1. Boudík F, Reissigová J, Hrach K, Tomečková M, Bultas J, Anger Z, et al. Primary prevention of coronary artery disease among middle aged men in prague: Twenty-year follow-up results. Atherosclerosis 2006;184(1):86-93. | B |
| 1. Bouillon K, Batty GD, Hamer M, Sabia S, Shipley MJ, Britton A, et al. Cardiovascular disease risk scores in identifying future frailty: The whitehall II prospective cohort study. Heart 2013;99(10):737-42. | B |
| 1. Boutouyrie P, Tropeano AI, Asmar R, Gautier I, Benetos A, Lacolley P, Laurent S. Aortic stiffness is an independent predictor of primary coronary events in hypertensive patients: A longitudinal study. Hypertension 2002;39(1):10-5. | D |
| 1. Boyar A. Creating a web application that combines framingham risk with electron beam CT coronary calcium score to calculate a new event risk. Journal of Thoracic Imaging 2006;21(1):91-6. | A |
| 1. Bozorgmanesh M, Hadaegh F, Azizi F. Predictive accuracy of the 'framingham's general CVD algorithm' in a middle eastern population: Tehran lipid and glucose study. International Journal of Clinical Practice 2011;65(3):264-73. | B |
| 1. Bozorgmanesh M, Hadaegh F, Azizi F. Predictive performances of lipid accumulation product vs. Adiposity measures for cardiovascular diseases and all-cause mortality, 8.6-year follow-up: Tehran lipid and glucose study. Lipids in Health and Disease 2010;9. | B |
| 1. Bozorgmanesh M. Added predictive ability of the information on job strain beyond the standard framingham risk score. International Journal of Epidemiology 2012;41(1):322-4. | A |
| 1. Brant LJ, Ferrucci L, Sheng SL, Concin H, Zonderman AB, Kelleher CC, et al. Gender differences in the accuracy of time-dependent blood pressure indices for predicting coronary heart disease: A random-effects modeling approach. Gender Medicine 2010;7(6):616-27. | A |
| 1. Brautbar A, Ballantyne CM, Lawson K, Nambi V, Chambless L, Folsom AR, et al. Impact of adding a single allele in the 9p21 locus to traditional risk factors on reclassification of coronary heart disease risk and implications for lipid-modifying therapy in the atherosclerosis risk in communities study. Circulation: Cardiovascular Genetics 2009;2(3):279-85. | A |
| 1. Brautbar A, Pompeii LA, Dehghan A, Ngwa JS, Nambi V, Virani SS, et al. A genetic risk score based on direct associations with coronary heart disease improves coronary heart disease risk prediction in the atherosclerosis risk in communities (ARIC), but not in the rotterdam and framingham offspring, studies. Atherosclerosis 2012;223(2):421-6. | A |
| 1. Brouwers FP, Asselbergs FW, Hillege HL, Gansevoort RT, de Boer RA, van Gilst WH. Elevated urinary albumin excretion complements the framingham risk score for the prediction of cardiovascular risk - response to treatment in the PREVEND IT trial. IJC Heart and Vessels 2014;4(1):193-7. | C |
| 1. Brown JM, Stewart JC, Stump TE, Callahan CM. Risk of coronary heart disease events over 15 years among older adults with depressive symptoms. American Journal of Geriatric Psychiatry 2011;19(8):721-9. | A |
| 1. Brügger-Andersen T, Aarsetøy H, Grundt H, Staines H, Nilsen DWT. The long-term prognostic value of multiple biomarkers following a myocardial infarction. Thrombosis Research 2008;123(1):60-6. | A |
| 1. Budoff MJ, Shaw LJ, Liu ST, Weinstein SR, Mosler TP, Tseng PH, et al. Long-Term prognosis associated with coronary calcification. Observations from a registry of 25,253 patients. Journal of the American College of Cardiology 2007;49(18):1860-70. | A |
| 1. Cabrera de León A, Alemán Sánchez JJ, Rodríguez Pérez MC, del Castillo-Rodríguez JC, Domínguez-Coello S, Almeida-González D, et al. Framingham function estimates the risk of cardio vascular mortality more effectively than SCORE function in the population of the canary islands (spain). Gaceta Sanitaria 2009;23(3):216-21. | C |
| 1. Calvo Bonacho E, Catalina Romero C, Fernández-Labandera Ramos C, Cortés Arcas MV, García Margallo MT, Sánchez-Chaparro MA. Clinical usefulness of a predictive model of cardiovascular risk among workers. Revista De La Asociacion Espanola De Especialistas En Medicina Del Trabajo 2018;27(3):133-40. | B |
| 1. Cameron AJ, Magliano DJ, Shaw JE, Zimmet PZ, Carstensen B, Alberti KGM, et al. The influence of hip circumference on the relationship between abdominal obesity and mortality. International Journal of Epidemiology 2012;41(2):484-94. | A |
| 1. Cañón Barroso L, Cruces Muro E, Fernández Gómez A, Nieto Hernández T, Fernández Ochoa G, Buitrago Ramírez F. Analysis of the concordance and validation of the framingham-regicor and DORICA equations in diabetic population in a health care center followed-up for 10 years. Semergen 2007;33(2):50-7. | A |
| 1. Canouï-Poitrine F, Luc G, Bard JM, Ferrieres J, Yarnell J, Arveiler D, et al. Relative contribution of lipids and apolipoproteins to incident coronary heart disease and ischemic stroke: The PRIME study. Cerebrovascular Diseases 2010;30(3):252-9. | A |
| 1. Cao JJ, Biggs ML, Barzilay J, Konen J, Psaty BM, Kuller L, et al. Cardiovascular and mortality risk prediction and stratification using urinary albumin excretion in older adults ages 68-102: The cardiovascular health study. Atherosclerosis 2008;197(2):806-13. | A |
| 1. Carreras-Torres R, Kundu S, Zanetti D, Esteban E, Via M, Moral P. Genetic risk score of NOS gene variants associated with myocardial infarction correlates with coronary incidence across europe. PLoS ONE 2014;9(5). | A |
| 1. Cazzola M, Calzetta L, Matera MG, Muscoli S, Rogliani P, Romeo F. Chronic obstructive pulmonary disease and coronary disease: COPDCoRi, a simple and effective algorithm for predicting the risk of coronary artery disease in COPD patients. Respiratory Medicine 2015;109(8):1019-25. | A |
| 1. Cedeño Mora S, Goicoechea M, Torres E, Verdalles, Pérez de José A, Verde E, et al. Cardiovascular risk prediction in chronic kidney disease patients. Nefrologia 2017;37(3):293-300. | A |
| 1. Cerecero P, Hernández-Prado B, Denova E, Valdés R, Vázquez G, Camarillo E, Huitrón G. Association between serum uric acid levels and cardiovascular risk among university workers from the state of mexico: A nested case-control study. BMC Public Health 2013;13(1). | A |
| 1. Chamberlain AM, Agarwal SK, Folsom AR, Soliman EZ, Chambless LE, Crow R, et al. A clinical risk score for atrial fibrillation in a biracial prospective cohort (from the atherosclerosis risk in communities [ARIC] study). American Journal of Cardiology 2011;107(1):85-91. | C |
| 1. Chambless LE, Folsom AR, Sharrett AR, Sorlie P, Couper D, Szklo M, Nieto FJ. Coronary heart disease risk prediction in the atherosclerosis risk in communities (ARIC) study. Journal of Clinical Epidemiology 2003;56(9):880-90. | A |
| 1. Chamnan P, Simmons RK, Hori H, Sharp S, Khaw KT, Wareham NJ, Grijfin SJ. A simple risk score using routine data for predicting cardiovascular disease in primary care. British Journal of General Practice 2010;60(577):e327-34. | B |
| 1. Chang A, Kramer H. Should egfr and albuminuria be added to the framingham risk score? Chronic kidney disease and cardiovascular disease risk prediction. Nephron - Clinical Practice 2011;119(2):c171-7. | A |
| 1. Chang X, Salim A, Dorajoo R, Han Y, Khor CC, Van Dam RM, et al. Utility of genetic and non- genetic risk factors in predicting coronary heart disease in singaporean chinese. European Journal of Preventive Cardiology 2017;24(2):153-60. | A |
| 1. Chen R, Yang Y, Miao F, Cai Y, Lin D, Zheng J, Li Y. 3-year risk prediction of coronary heart disease in hypertension patients: A preliminary study; 39th annual international conference of the IEEE engineering in medicine and biology society, EMBC 2017. Institute of Electrical and Electronics Engineers Inc.; 2017al. | C |
| 1. Chen Z, Yang G, Zhou M, Smith M, Offer A, Ma J, et al. Body mass index and mortality from ischaemic heart disease in a lean population: 10 year prospective study of 220 000 adult men. International Journal of Epidemiology 2006;35(1):141-50. | A |
| 1. Chen ZW, Chen YH, Qian JY, Ma JY, Ge JB. Validation of a novel clinical prediction score for severe coronary artery diseases before elective coronary angiography. PLoS ONE 2014;9(4). | A |
| 1. Cheng VY, Dey D, Tamarappoo B, Nakazato R, Gransar H, Miranda-Peats R, et al. Pericardial fat burden on ecg-gated noncontrast CT in asymptomatic patients who subsequently experience adverse cardiovascular events. JACC: Cardiovascular Imaging 2010;3(4):352-60. | C |
| 1. Cheruvu C, Precious B, Naoum C, Blanke P, Ahmadi A, Soon J, et al. Long term prognostic utility of coronary CT angiography in patients with no modifiable coronary artery disease risk factors: Results from the 5 year follow-up of the CONFIRM international multicenter registry. Journal of Cardiovascular Computed Tomography 2016;10(1):22-7. | A |
| 1. Chia YC, Gray SYW, Ching SM, Lim HM, Chinna K. Validation of the framingham general cardiovascular risk score in a multiethnic asian population: A retrospective cohort study. BMJ Open 2015;5(5). | B |
| 1. Chien KL, Sung FC, Hsu HC, Su TC, Chang WD, Lee YT. Relative importance of atherosclerotic risk factors for coronary heart disease in taiwan. European Journal of Cardiovascular Prevention and Rehabilitation 2005;12(2):95-101. | A |
| 1. Chimonas T, Athyros VG, Ganotakis E, Nicolaou V, Panagiotakos DB, Mikhailidis DP, Elisaf M. Cardiovascular risk factors and estimated 10-year risk of fatal cardiovascular events using various equations in greeks with metabolic syndrome. Angiology 2010;61(1):49-57. | A |
| 1. Chironi G, Simon A, Megnien JL, Sirieix ME, Mousseaux E, Pessana F, Armentano R. Impact of coronary artery calcium on cardiovascular risk categorization and lipid-lowering drug eligibility in asymptomatic hypercholesterolemic men. International Journal of Cardiology 2011;151(2):200-4. | A |
| 1. Cho EJ, Park SJ, Chang SA, Jeong DS, Lee SC, Park SW, Park PW. Incidence of coronary artery disease before valvular surgery in isolated severe aortic stenosis. Chinese Medical Journal 2014;127(22):3963-9. | A |
| 1. Cho IJ, Sung JM, Chang HJ, Chung N, Kim HC. Incremental value of repeated risk factor measurements for cardiovascular disease prediction in middle-aged korean adults: Results from the NHIS-HEALS (national health insurance system-national health screening cohort). Circulation: Cardiovascular Quality and Outcomes 2017;10(11). | A |
| 1. Chow CK, Joshi R, Celermajer DS, Patel A, Neal BC. Recalibration of a framingham risk equation for a rural population in india. Journal of Epidemiology and Community Health 2009;63(5):379-85. | A |
| 1. Chow EJ, Chen Y, Hudson MM, Feijen EAM, Kremer LC, Border WL, et al. Prediction of ischemic heart disease and stroke in survivors of childhood cancer. Journal of Clinical Oncology 2018;36(1):44-52. | A |
| 1. Christianson TJH, Bryant SC, Weymiller AJ, Smith SA, Montori VM. A pen-and-paper coronary risk estimator for office use with patients with type 2 diabetes. Mayo Clinic Proceedings 2006;81(5):632-8. | A |
| 1. Chuang ML, Gona P, Salton CJ, Yeon SB, Kissinger KV, Blease SJ, et al. Usefulness of the left ventricular myocardial contraction fraction in healthy men and women to predict cardiovascular morbidity and mortality. American Journal of Cardiology 2012;109(10):1454-8. | C |
| 1. Colaco K, Lee KA, Akhtari S, Winer R, Welsh P, Sattar N, et al. Targeted metabolomic profiling and prediction of cardiovascular events: A prospective study of patients with psoriatic arthritis and psoriasis. Annals of the Rheumatic Diseases 2021;80(11):1429-35. | A |
| 1. Conroy RM, Pyörälä K, Fitzgerald AP, Sans S, Menotti A, De Backer G, et al. Estimation of ten- year risk of fatal cardiovascular disease in europe: The SCORE project. European Heart Journal 2003;24(11):987-1003. | A |
| 1. Conroy RM, Pyörälä K, Fitzgerald AP, Sans S, Menotti A, De Backer G, et al. Estimation of ten- year risk of fatal cardiovascular disease in europe: The SCORE project. European Heart Journal 2003;24(11):987-1003. | A |
| 1. Cook NR, Buring JE, Ridker PM. The effect of including c-reactive protein in cardiovascular risk prediction models for women. Annals of Internal Medicine 2006;145(1):21-9. | A |
| 1. Cooper H, Wells S, Mehta S. Are competing-risk models superior to standard cox models for predicting cardiovascular risk in older adults? Analysis of a whole-of-country primary prevention cohort aged ≥65 years. International Journal of Epidemiology 2022;51(2):604-14. | A |
| 1. Cosson E, Nguyen MT, Chanu B, Banu I, Chiheb S, Balta C, et al. Cardiovascular risk prediction is improved by adding asymptomatic coronary status to routine risk assessment in type 2 diabetic patients. Diabetes Care 2011;34(9):2101-7. | C |
| 1. Cross DS, McCarty CA, Hytopoulos E, Beggs M, Nolan N, Harrington DS, et al. Coronary risk assessment among intermediate risk patients using a clinical and biomarker based algorithm developed and validated in two population cohorts. Current Medical Research and Opinion 2012;28(11):1819-30. | A |
| 1. Cross DS, McCarty CA, Steinhubl SR, Carey DJ, Erlich PM. Development of a multi-institutional cohort to facilitate cardiovascular disease biomarker validation using existing biorepository samples linked to electronic health records. Clinical Cardiology 2013;36(8):486-91. | C |
| 1. Crowson CS, Gabriel SE, Semb AG, van Riel PLCM, Karpouzas G, Dessein PH, et al. Rheumatoid arthritis-specific cardiovascular risk scores are not superior to general risk scores: A validation analysis of patients from seven countries. Rheumatology 2017;56(7):1102-10. | B |
| 1. Cui J, Forbes A, Kirby A, Simes J, Tonkin A. Laboratory and non-laboratory-based risk prediction models for secondary prevention of cardiovascular disease: The LIPID study. European Journal of Cardiovascular Prevention and Rehabilitation 2009;16(6):660-8. | A |
| 1. Cui Y, Blumenthal RS, Flaws JA, Whiteman MK, Langenberg P, Bachorik PS, Bush TL. Non-high- density lipoprotein cholesterol level as a predictor of cardiovascular disease mortality. Archives of Internal Medicine 2001;161(11):1413-9. | A |
| 1. Culleton BF, Larson MG, Kannel WB, Levy D. Serum uric acid and risk for cardiovascular disease and death: The framingham heart study. Annals of Internal Medicine 1999;131(1):7-13. | A |
| 1. Cunningham R, Poppe K, Peterson D, Every-Palmer S, Soosay I, Jackson R. Prediction of cardiovascular disease risk among people with severe mental illness: A cohort study. PLoS ONE 2019;14(9). | A |
| 1. D'Agostino Sr RB, Grundy S, Sullivan LM, Wilson P. Validation of the framingham coronary heart disease prediction scores: Results of a multiple ethnic groups investigation. JAMA - Journal of the American Medical Association 2001;286(2):180-7. | A |
| 1. Dai J, Krasnow RE, Reed T. Midlife moderation-quantified healthy diet and 40-year mortality risk from CHD: The prospective national heart, lung, and blood institute twin study. British Journal of Nutrition 2016;116(2):326-34. | C |
| 1. DarbÃ J, Kaskens L, Aranda P, Arango C, Bobes J, Carmena R, Rejas J. A simulation model to estimate 10-year risk of coronary heart disease events in patients with schizophrenia spectrum disorders treated with second-generation antipsychotic drugs. Annals of Clinical Psychiatry 2013;25(1):17-26. | A |
| 1. Davidson KW, Mostofsky E, Whang W. Don't worry, be happy: Positive affect and reduced 10- year incident coronary heart disease: The canadian nova scotia health survey. European Heart Journal 2010;31(9):1065-70. | A |
| 1. Davidson KW, Mostofsky E. Anger expression and risk of coronary heart disease: Evidence from the nova scotia health survey. American Heart Journal 2010;159(2):199-206. | A |
| 1. Davidson KW, Schwartz JE, Kirkland SA, Mostofsky E, Fink D, Guernsey D, Shimbo D. Relation of inflammation to depression and incident coronary heart disease (from the canadian nova scotia health survey [NSHS95] prospective population study). American Journal of Cardiology 2009;103(6):755-61. | A |
| 1. Daviglus ML, Stamler J, Pirzada A, Yan LL, Garside DB, Liu K, et al. Favorable cardiovascular risk profile in young women and long-term risk of cardiovascular and all-cause mortality. JAMA - Journal of the American Medical Association 2004;292(13):1588-92. | A |
| 1. Davis WA, Knuiman MW, Davis TME. An australian cardiovascular risk equation for type 2 diabetes: The fremantle diabetes study. Internal Medicine Journal 2010;40(4):286-92. | A |
| 1. de Freitas EV, Brandão AA, Pozzan R, Magalhães ME, Fonseca F, Pizzi O, et al. Importance of high-density lipoprotein-cholesterol (HDL-C) levels to the incidence of cardiovascular disease (CVD) in the elderly. Archives of Gerontology and Geriatrics 2011;52(2):217-22. | A |
| 1. De Freitas EV, Brandão AA, Pozzan R, Magalhães ME, Fonseca F, Pizzi O, et al. Importance of hdl-c for the occurrence of cardiovascular disease in the elderly. Arquivos Brasileiros De Cardiologia 2009;93(3):216-222+227-234+231-238. | A |
| 1. de Hartog-Keyzer JML, Pedersen SS, El Messaoudi S, Nijveldt R, Pop VJM. Psychological distress is independently related to new coronary events at 8 years' follow-up in elderly primary care patients with hypertension. Journal of Psychosomatic Research 2022;160. | A |
| 1. De Vries PS, Kavousi M, Ligthart S, Uitterlinden AG, Hofman A, Franco OH, Dehghan A. Incremental predictive value of 152 single nucleotide polymorphisms in the 10-year risk prediction of incident coronary heart disease: The rotterdam study. International Journal of Epidemiology 2015;44(2):682-8. | A |
| 1. Defina LF, Radford NB, Barlow CE, Willis BL, Leonard D, Haskell WL, et al. Association of all- cause and cardiovascular mortality with high levels of physical activity and concurrent coronary artery calcification. JAMA Cardiology 2019;4(2):174-81. | A |
| 1. Dekker JM, Girman C, Rhodes T, Nijpels G, Stehouwer CDA, Bouter LM, Heine RJ. Metabolic syndrome and 10-year cardiovascular disease risk in the hoorn study. Circulation 2005;112(5):666-73. | C |
| 1. Denova-Gutiérrez E, Tucker KL, Flores M, Barquera S, Salmerón J. Dietary patterns are associated with predicted cardiovascular disease risk in an urban mexican adult population. Journal of Nutrition 2016;146(1):90-7. | A |
| 1. Deseive S, Shaw LJ, Min JK, Achenbach S, Andreini D, Al-Mallah MH, et al. Improved 5-year prediction of all-cause mortality by coronary CT angiography applying the CONFIRM score. European Heart Journal Cardiovascular Imaging 2017;18(3):286-93. | C |
| 1. Detrano R, Guerci AD, Carr JJ, Bild DE, Burke G, Folsom AR, et al. Coronary calcium as a predictor of coronary events in four racial or ethnic groups. New England Journal of Medicine 2008;358(13):1336-45. | A |
| 1. Dhaliwal SS, Welborn TA. Central obesity and cigarette smoking are key determinants of cardiovascular disease deaths in australia: A public health perspective. Preventive Medicine 2009;49(2-3):153-7. | B |
| 1. Dhamoon MS, Moon YP, Paik MC, Sacco RL, Elkind MSV. The inclusion of stroke in risk stratification for primary prevention of vascular events: The northern manhattan study. Stroke 2011;42(10):2878-82. | B |
| 1. Dogan MV, Beach SRH, Simons RL, Lendasse A, Penaluna B, Philibert RA. Blood-based biomarkers for predicting the risk for five-year incident coronary heart disease in the framingham heart study via machine learning. Genes 2018;9(12). | D |
| 1. Dogan MV, Grumbach IM, Michaelson JJ, Philibert RA. Integrated genetic and epigenetic prediction of coronary heart disease in the framingham heart study. PLoS ONE 2018;13(1). | A |
| 1. Dogan MV, Knight S, Dogan TK, Knowlton KU, Philibert R. External validation of integrated genetic-epigenetic biomarkers for predicting incident coronary heart disease. Epigenomics 2021;13(14):1095-112. | D |
| 1. Doukky R, Frogge N, Balakrishnan G, Hayes K, Collado FM, Rangel MO, et al. The prognostic value of cardiac SPECT performed at the primary care physician's office. Journal of Nuclear Cardiology 2013;20(4):519-28. | D |
| 1. Doukky R, Hayes K, Frogge N, Balakrishnan G, Dontaraju VS, Rangel MO, et al. Impact of appropriate use on the prognostic value of single-photon emission computed tomography myocardial perfusion imaging. Circulation 2013;128(15):1634-43. | D |
| 1. Du B, Si D, Zhao D, Zhao Y, Wagatsuma K, He Y, Yang P. A new peripheral endothelial function measurement improves prediction of symptomatic coronary artery disease. Journal of International Medical Research 2020;48(6). | B |
| 1. Dutta A, Henley W, Lang IA, Murray A, Guralnik J, Wallace RB, Melzer D. The coronary artery disease-associated 9p21 variant and later life 20-year survival to cohort extinction. Circulation: Cardiovascular Genetics 2011;4(5):542-8. | C |
| 1. Dziopa K, Asselbergs FW, Gratton J, Chaturvedi N, Schmidt AF. Cardiovascular risk prediction in type 2 diabetes: A comparison of 22 risk scores in primary care settings. Diabetologia 2022;65(4):644-56. | C |
| 1. Eberly LE, Cohen JD, Prineas R, Yang L. Impact of incident diabetes and incident nonfatal cardiovascular disease on 18-year mortality: The multiple risk factor intervention trial experience. Diabetes Care 2003;26(3):848-54. | A |
| 1. El Harchaoui K, van der Steeg WA, Stroes ESG, Kuivenhoven JA, Otvos JD, Wareham NJ, et al. Value of low-density lipoprotein particle number and size as predictors of coronary artery disease in apparently healthy men and women. The epic-norfolk prospective population study. Journal of the American College of Cardiology 2007;49(5):547-53. | A |
| 1. Elias-Smale SE, Proença RV, Koller MT, Kavousi M, Van Rooij FJA, Hunink MG, et al. Coronary calcium score improves classification of coronary heart disease risk in the elderly: The rotterdam study. Journal of the American College of Cardiology 2010;56(17):1407-14. | A |
| 1. Ellims AH, Wong G, Weir JM, Lew P, Meikle PJ, Taylor AJ. Plasma lipidomic analysis predicts non-calcified coronary artery plaque in asymptomatic patients at intermediate risk of coronary artery disease. European Heart Journal Cardiovascular Imaging 2014;15(8):908-16. | B |
| 1. Elsayed HAG, Galal MA, Syed L. HeartCare+: A smart heart care mobile application for framingham-based early risk prediction of hard coronary heart diseases in middle east. Mobile Information Systems 2017;2017. | A |
| 1. Empana JP, Tafflet M, Escolano S, Vergnaux AC, Bineau S, Ruidavets JB, et al. Predicting CHD risk in france: A pooled analysis of the D.E.S.I.R., Three city, PRIME, and SU.VI.MAX studies. European Journal of Cardiovascular Prevention and Rehabilitation 2011;18(2):175-85. | B |
| 1. Erbel R, Budoff M. Improvement of cardiovascular risk prediction using coronary imaging: Subclinical atherosclerosis: The memory of lifetime risk factor exposure. European Heart Journal 2012;33(10):1201-17. | A |
| 1. Erbel R, Eisele L, Moebus S, Dragano N, Möhlenkamp S, Bauer M, et al. The heinz nixdorf recall study. Bundesgesundheitsblatt - Gesundheitsforschung - Gesundheitsschutz 2012;55(6-7):809-15. | A |
| 1. Erikssen G, Bodegard J, Bjørnholt JV, Liestøl K, Thelle DS, Erikssen J. Exercise testing of healthy men in a new perspective: From diagnosis to prognosis. European Heart Journal 2004;25(11):978-86. | A |
| 1. Ernste FC, Sánchez-Menéndez M, Wilton KM, Crowson CS, Matteson EL, Maradit Kremers H. Cardiovascular risk profile at the onset of psoriatic arthritis: A population-based cohort study. Arthritis Care and Research 2015;67(7):1015-21. | B |
| 1. Esteghamati A, Hafezi-Nejad N, Sheikhbahaei S, Heidari B, Zandieh A, Ebadi M, Nakhjavani M. Risk of coronary heart disease associated with metabolic syndrome and its individual components in iranian subjects: A matched cohort study. Journal of Clinical Lipidology 2014;8(3):279-86. | A |
| 1. Everett BM, Kurth T, Buring JE, Ridker PM. The relative strength of c-reactive protein and lipid levels as determinants of ischemic stroke compared with coronary heart disease in women. Journal of the American College of Cardiology 2006;48(11):2235-42. | A |
| 1. Faghihi-Kashani S, Bonnet F, Hafezi-Nejad N, Heidari B, Aghajani Nargesi A, Sheikhbahaei S, et al. Fasting hyperinsulinaemia and 2-h glycaemia predict coronary heart disease in patients with type 2 diabetes. Diabetes and Metabolism 2016;42(1):55-61. | A |
| 1. Fatema K, Rahman B, Zwar NA, Milton AH, Ali L. Short-term predictive ability of selected cardiovascular risk prediction models in a rural bangladeshi population: A case-cohort study. BMC Cardiovascular Disorders 2016;16(1). | D |
| 1. Fazelzadeh A, Mehdizadeh A, Ostovan MA, Raiss-Jalali GA. Incidence of cardiovascular risk factors and complications before and after kidney transplantation. Transplantation Proceedings 2006;38(2):506-8. | A |
| 1. Feldman B, Orbach-Zinger S, Leventer-Roberts M, Hoshen M, Dagan N, Balicer R, Eidelman LA. Maternal age and cardiovascular and metabolic disease outcomes: A retrospective cohort study using data from population-based electronic medical records. Journal of Maternal-Fetal and Neonatal Medicine 2020;33(11):1853-60. | A |
| 1. Feng Y, Huang X, Sun H, Liu C, Zhang B, Zhang Z, et al. Framingham risk score modifies the effect of PM<inf>10</inf> on heart rate variability. Science of the Total Environment 2015;523:146-51. | C |
| 1. Ferket BS, Van Kempen BJH, Hunink MGM, Agarwal I, Kavousi M, Franco OH, et al. Predictive value of updating framingham risk scores with novel risk markers in the U.S. General population. PLoS ONE 2014;9(2). | A |
| 1. Filev PD, Stillman AE. Long-Term prognostic value of stress perfusion cardiovascular magnetic resonance imaging. Current Treatment Options in Cardiovascular Medicine 2019;21(10). | A |
| 1. Filippella M, Lillaz E, Ciccarelli A, Giardina S, Massimetti E, Navaretta F, et al. Ankle brachial pressure index usefulness as predictor factor for coronary heart disease in diabetic patients. Journal of Endocrinological Investigation 2007;30(9):721-5. | A |
| 1. Findlay SG, Kasliwal RR, Bansal M, Tarique A, Zaman A. A comparison of cardiovascular risk scores in native and migrant south asian populations. SSM - Population Health 2020;11. | C |
| 1. Folsom AR, Chambless LE, Ballantyne CM, Coresh J, Heiss G, Wu KK, et al. An assessment of incremental coronary risk prediction using c-reactive protein and other novel risk markers: The atherosclerosis risk in communities study. Archives of Internal Medicine 2006;166(13):1368-73. | A |
| 1. Folsom AR, Chambless LE, Duncan BB, Gilbert AC, Pankow JS. Prediction of coronary heart disease in middle-aged adults with diabetes. Diabetes Care 2003;26(10):2777-84. | A |
| 1. Ford ES, Giles WH, Mokdad AH. The distribution of 10-year risk for coronary heart disease among U.S. Adults: Findings from the national health and nutrition examination survey III. Journal of the American College of Cardiology 2004;43(10):1791-6. | A |
| 1. Ford ES. Trends in predicted 10-year risk of coronary heart disease and cardiovascular disease among U.S. Adults from 1999 to 2010. Journal of the American College of Cardiology 2013;61(22):2249-52. | A |
| 1. Forte JC, Folkertsma P, Gannamani R, Kumaraswamy S, Mount S, de Koning TJ, et al. Development and validation of decision rules models to stratify coronary artery disease, diabetes, and hypertension risk in preventive care: Cohort study of returning UK biobank participants. Journal of Personalized Medicine 2021;11(12). | C |
| 1. Fovino LN, Saladini G, Cervino AR, Saladini F, Gregianin M, Razzolini R, Evangelista L. Prognostic value of myocardial perfusion scintigraphy in elderly patients with hypertension: A 10- year follow-up analysis. European Journal of Nuclear Medicine and Molecular Imaging 2012;39(10):1570-80. | A |
| 1. Fox ER, Samdarshi TE, Musani SK, Pencina MJ, Sung JH, Bertoni AG, et al. Development and validation of risk prediction models for cardiovascular events in black adults: The jackson heart study cohort. JAMA Cardiology 2016;1(1):15-25. | B |
| 1. Franco J, Leahy F, editors. Discriminative and generative models for clinical risk estimation: An empirical comparison; 6th imperial college computing student workshop, ICCSW 2017. Schloss Dagstuhl- Leibniz-Zentrum fur Informatik GmbH, Dagstuhl Publishing; 2018m. | A |
| 1. Frary CE, Blicher MK, Olesen TB, Stidsen JV, Greve SV, Vishram-Nielsen JKK, et al. Circulating biomarkers for long-term cardiovascular risk stratification in apparently healthy individuals from the MONICA 10 cohort. European Journal of Preventive Cardiology 2020;27(6):570-8. | A |
| 1. Freiberg MS, Chang CCH, Kuller LH, Skanderson M, Lowy E, Kraemer KL, et al. HIV infection and the risk of acute myocardial infarction. JAMA Internal Medicine 2013;173(8):614-22. | C |
| 1. Frikke-Schmidt R, Tybjærg-Hansen A, Schnohr P, Jensen GB, Nordestgaard BG. Common clinical practice versus new PRIM score in predicting coronary heart disease risk. Atherosclerosis 2010;213(2):532-8. | A |
| 1. Fujihara K, Suzuki H, Sato A, Ishizu T, Kodama S, Heianza Y, et al. Comparison of the framingham risk score, UK prospective diabetes study (UKPDS) risk engine, japanese atherosclerosis longitudinal study-existing cohorts combine (JALS-ECC) and maximum carotid intima-media thickness for predicting coronary artery stenosis in patients with asymptomatic type 2 diabetes. Journal of Atherosclerosis and Thrombosis 2014;21(8):799-815. | A |
| 1. Gadepally V, Mattson T, Stonebraker M, Wang F, Luo G, Laing Y, Dubovitskaya A. Heterogeneous Data Management, Polystores, and Analytics for Healthcare: VLDB 2018 Workshops, Poly and DMAH, Rio De Janeiro, Brazil, August 31, 2018, Revised Selected Papers. Springer, 2019. | C |
| 1. Gallo WT, Teng HM, Falba TA, Kasl SV, Krumholz HM, Bradley EH. The impact of late career job loss on myocardial infarction and stroke: A 10 year follow up using the health and retirement survey. Occupational and Environmental Medicine 2006;63(10):683-7. | A |
| 1. Game FL, Bartlett WA, Bayly GR, Jones AF. Comparative accuracy of cardiovascular risk prediction methods in patients with diabetes mellitus. Diabetes, Obesity and Metabolism 2001;3(4):279-86. | A |
| 1. Ganna A, Magnusson PKE, Pedersen NL, De Faire U, Reilly M, Ärnlöv J, et al. Multilocus genetic risk scores for coronary heart disease prediction. Arteriosclerosis, Thrombosis, and Vascular Biology 2013;33(9):2267-72. | A |
| 1. Ganna A, Salihovic S, Sundström J, Broeckling CD, Hedman K, Magnusson PKE, et al. Large- scale metabolomic profiling identifies novel biomarkers for incident coronary heart disease. PLoS Genetics 2014;10(12). | A |
| 1. Geibe JR, Holder J, Peeples L, Kinney AM, Burress JW, Kales SN. Predictors of on-duty coronary events in male firefighters in the united states. American Journal of Cardiology 2008;101(5):585-9. | A |
| 1. Geisel MH, Bauer M, Hennig F, Hoffmann B, Lehmann N, Möhlenkamp S, et al. Comparison of coronary artery calcification, carotid intima-media thickness and ankle-brachial index for predicting 10-year incident cardiovascular events in the general population. European Heart Journal 2017;38(23):1815-22. | C |
| 1. Ghasemzadeh N, Brooks MM, Vlachos H, Hardison R, Sikora S, Sperling L, et al. An aggregate biomarker risk score predicts high risk of near-term myocardial infarction and death: Findings from BARI 2D (bypass angioplasty revascularization investigation 2 diabetes). Journal of the American Heart Association 2017;6(7). | A |
| 1. Gil-Guillen V, Orozco-Beltran D, Redon J, Pita-Fernandez S, Navarro-Pérez J, Pallares V, et al. Rationale and methods of the cardiometabolic valencian study (escarval-risk) for validation of risk scales in mediterranean patients with hypertension, diabetes or dyslipidemia. BMC Public Health 2010;10. | A |
| 1. Glazer NL, Smith NL, Heckbert SR, Doggen CJM, Lemaitre RN, Psaty BM. Risk of myocardial infarction attributable to elevated levels of total cholesterol among hypertensives. American Journal of Hypertension 2005;18(6):759-66. | B |
| 1. Glynn RJ, Chae CU, Guralnik JM, Taylor JO, Hennekens CH. Pulse pressure and mortality in older people. Archives of Internal Medicine 2000;160(18):2765-72. | A |
| 1. Glynn RJ, L'Italien GJ, Sesso HD, Jackson EA, Buring JE. Development of predictive models for long-term cardiovascular risk associated with systolic and diastolic blood pressure. Hypertension 2002;39(1):105-10. | A |
| 1. Glynn RJ, Rosner B. Comparison of risk factors for the competing risks of coronary heart disease, stroke, and venous thromboembolism. American Journal of Epidemiology 2005;162(10):975-82. | A |
| 1. Goldman A, Hod H, Chetrit A, Dankner R. Incidental abnormal ECG findings and long-term cardiovascular morbidity and all-cause mortality: A population based prospective study. International Journal of Cardiology 2019;295:36-41. | A |
| 1. Goldman O, Raphaeli O, Goldman E, Leshno M. Improvement in the prediction of coronary heart disease risk by using artificial neural networks. Quality Management in Health Care 2021;30(4):244-50. | B |
| 1. González-Diego P, Moreno-Iribas C, Guembe MJ, Viñes JJ, Vila J. Adaptation of the framingham-wilson coronary risk equation for the population of navarra (RICORNA). Revista Espanola De Cardiologia 2009;62(8):875-85. | A |
| 1. Gopinath B, Flood VM, Rochtchina E, Thiagalingam A, Mitchell P. Serum homocysteine and folate but not vitamin B12 are predictors of CHD mortality in older adults. European Journal of Preventive Cardiology 2012;19(6):1420-9. | A |
| 1. Gorostegi-Anduaga I, Maldonado-Martín S, MartinezAguirre-Betolaza A, Corres P, Romaratezabala E, Whittaker AC, et al. Effects on cardiovascular risk scores and vascular age after aerobic exercise and nutritional intervention in sedentary and overweight/obese adults with primary hypertension: The EXERDIET-HTA randomized trial study. High Blood Pressure and Cardiovascular Prevention 2018;25(4):361-8. | B |
| 1. Gotto Jr AM, Whitney E, Stein EA, Shapiro DR, Clearfield M, Weis S, et al. Relation between baseline and on-treatment lipid parameters and first acute major coronary events in the air force/texas coronary atherosclerosis prevention study (AFCAPS/texcaps). Circulation 2000;101(5):477-84. | A |
| 1. Graham G, Blaha MJ, Budoff MJ, Rivera JJ, Agatston A, Raggi P, et al. Impact of coronary artery calcification on all-cause mortality in individuals with and without hypertension. Atherosclerosis 2012;225(2):432-7. | A |
| 1. Greenland P, Knoll MD, Stamler J, Neaton JD, Dyer AR, Garside DB, Wilson PW. Major risk factors as antecedents of fatal and nonfatal coronary heart disease events. JAMA - Journal of the American Medical Association 2003;290(7):891-7. | A |
| 1. Greenland P, LaBree L, Azen SP, Doherty TM, Detrano RC. Coronary artery calcium score combined with framingham score for risk prediction in asymptomatic individuals. JAMA - Journal of the American Medical Association 2004;291(2):210-5. | B |
| 1. Greenland P, Smith Jr SC, Grundy SM. Improving coronary heart disease risk assessment in asymptomatic people: Role of traditional risk factors and noninvasive cardiovascular tests. Circulation 2001;104(15):1863-7. | A |
| 1. Gronewold J, Hermann DM, Lehmann N, Kröger K, Lauterbach K, Berger K, et al. Ankle- brachial index predicts stroke in the general population in addition to classical risk factors. Atherosclerosis 2014;233(2):545-50. | C |
| 1. Grønhøj MH, Gerke O, Mickley H, Steffensen FH, Lambrechtsen J, Sand NPR, et al. External validity of a cardiovascular screening including a coronary artery calcium examination in middle- aged individuals from the general population. European Journal of Preventive Cardiology 2018;25(11):1156-66. | A |
| 1. Grunau GL, Sheps S, Goldner EM, Ratner PA. Specific comorbidity risk adjustment was a better predictor of 5-year acute myocardial infarction mortality than general methods. Journal of Clinical Epidemiology 2006;59(3):274-80. | A |
| 1. Grundy SM, D'Agostino Sr RB, Mosca L, Burke GL, Wilson PWF, Rader DJ, et al. Cardiovascular risk assessment based on US cohort studies: Findings from a national heart, lung, and blood institute workshop. Circulation 2001;104(4):491-6. | A |
| 1. Gulati G, Upshaw J, Wessler BS, Brazil RJ, Nelson J, Van Klaveren D, et al. Generalizability of cardiovascular disease clinical prediction models: 158 independent external validations of 104 unique models. Circulation: Cardiovascular Quality and Outcomes 2022;15(4):E008487. | D |
| 1. Gulati M, Arnsdorf MF, Shaw LJ, Pandey DK, Thisted RA, Lauderdale DS, et al. Prognostic value of the duke treadmill score in asymptomatic women. American Journal of Cardiology 2005;96(3):369-75. | C |
| 1. Gulati M, Pandey DK, Arnsdorf MF, Lauderdale DS, Thisted RA, Wicklund RH, et al. Exercise capacity and the risk of death in women: The st. James women take heart project. Circulation 2003;108(13):1554-9. | C |
| 1. Guthrie JR, Taffe JR, Lehert P, Burger HG, Dennerstein L. Association between hormonal changes at menopause and the risk of a coronary event: A longitudinal study. Menopause 2004;11(3):315-22. | A |
| 1. Hadaegh F, Hatami M, Sheikholeslami F, Azizi F. Pulse pressure and systolic blood pressure are powerful independent predictors of cardiovascular disease in diabetic adults: Results of an 8.4 years follow-up of tehran lipid and glucose study (TLGS). Experimental and Clinical Endocrinology and Diabetes 2010;118(9):638-43. | A |
| 1. Hadamitzky M, Achenbach S, Al-Mallah M, Berman D, Budoff M, Cademartiri F, et al. Optimized prognostic score for coronary computed tomographic angiography: Results from the CONFIRM registry (coronary CT angiography evaluation for clinical outcomes: An international multicenter registry). Journal of the American College of Cardiology 2013;62(5):468-76. | C |
| 1. Hadamitzky M, Distler R, Meyer T, Hein F, Kastrati A, Martinoff S, et al. Prognostic value of coronary computed tomographic angiography in comparison with calcium scoring and clinical risk scores. Circulation: Cardiovascular Imaging 2011;4(1):16-23. | D |
| 1. Hadamitzky M, Freißmuth B, Meyer T, Hein F, Kastrati A, Martinoff S, et al. Prognostic value of coronary computed tomographic angiography for prediction of cardiac events in patients with suspected coronary artery disease. JACC: Cardiovascular Imaging 2009;2(4):404-11. | D |
| 1. Hadamitzky M, Hein F, Meyer T, Bischoff B, Martinoff S, Schömig A, Hausleiter J. Prognostic value of coronary computed tomographic angiography in diabetic patients without known coronary artery disease. Diabetes Care 2010;33(6):1358-63. | D |
| 1. Hadamitzky M, Meyer T, Hein F, Bischoff B, Martinoff S, Schömig A, Hausleiter J. Prognostic value of coronary computed tomographic angiography in asymptomatic patients. American Journal of Cardiology 2010;105(12):1746-51. | D |
| 1. Hall SA, Shackelton R, Rosen RC, Araujo AB. Sexual activity, erectile dysfunction, and incident cardiovascular events. American Journal of Cardiology 2010;105(2):192-7. | A |
| 1. Haluska BA, Jeffries L, Carlier S, Marwick TH. Measurement of arterial distensibility and compliance to assess prognosis. Atherosclerosis 2010;209(2):474-80. | A |
| 1. Hamdan H, Boubiche DE, Hidoussi F, editors. An automatic early risk classification of hard coronary heart diseases using framingham scoring model; 2nd international conference on internet of things and cloud computing, ICC 2017. Association for Computing Machinery; 2017aj. | A |
| 1. Han D, Kolli KK, Gransar H, Lee JH, Choi SY, Chun EJ, et al. Machine learning based risk prediction model for asymptomatic individuals who underwent coronary artery calcium score: Comparison with traditional risk prediction approaches. Journal of Cardiovascular Computed Tomography 2020;14(2):168-76. | A |
| 1. Hansen TW, Staessen JA, Torp-Pedersen C, Rasmussen S, Li Y, Dolan E, et al. Ambulatory arterial stiffness index predicts stroke in a general population. Journal of Hypertension 2006;24(11):2247-53. | A |
| 1. Hansen TW, Staessen JA, Torp-Pedersen C, Rasmussen S, Thijs L, Ibsen H, Jeppesen J. Prognostic value of aortic pulse wave velocity as index of arterial stiffness in the general population. Circulation 2006;113(5):664-70. | A |
| 1. Haq IU, Ramsay LE, Jackson PR, Wallis EJ. Prediction of coronary risk for primary prevention of coronary heart disease: A comparison of methods. QJM - Monthly Journal of the Association of Physicians 1999;92(7):379-85. | B |
| 1. Hassinen M, Komulainen P, Lakka TA, Väisänen SB, Haapala I, Gylling H, et al. Metabolic syndrome and the progression of carotid intima-media thickness in elderly women. Archives of Internal Medicine 2006;166(4):444-9. | C |
| 1. Hathaway QA, Yanamala N, Budoff MJ, Sengupta PP, Zeb I. Deep neural survival networks for cardiovascular risk prediction: The multi-ethnic study of atherosclerosis (MESA). Computers in Biology and Medicine 2021;139. | A |
| 1. Hedayatnia M, Asadi Z, Zare-Feyzabadi R, Yaghooti-Khorasani M, Ghazizadeh H, Ghaffarian- Zirak R, et al. Dyslipidemia and cardiovascular disease risk among the MASHAD study population. Lipids in Health and Disease 2020;19(1). | A |
| 1. Heidemann C, Hoffmann K, Klipstein-Grobusch K, Weikert C, Pischon T, Hense HW, Boeing H. Potentially modifiable classic risk factors and their impact on incident myocardial infarction: Results from the epic-potsdam study. European Journal of Cardiovascular Prevention and Rehabilitation 2007;14(1):65-71. | A |
| 1. Hense HW, Schulte H, Löwel H, Assmann G, Keil U. Framingham risk function overestimates risk of coronary heart disease in men and women from germany - results from the MONICA augsburg and the PROCAM cohorts. European Heart Journal 2003;24(10):937-45. | B |
| 1. Hermann DM, Gronewold J, Lehmann N, Seidel UK, Möhlenkamp S, Weimar C, et al. Intima-media thickness predicts stroke risk in the heinz nixdorf recall study in association with vascular risk factors, age and gender. Atherosclerosis 2012;224(1):84-9. | C |
| 1. Hermansson J, Kahan T. Systematic review of validity assessments of framingham risk score results in health economic modelling of lipid-modifying therapies in europe. PharmacoEconomics 2018;36(2):205-13. | A |
| 1. Hermes W, Tamsma JT, Grootendorst DC, Franx A, van der Post J, van Pampus MG, et al. Cardiovascular risk estimation in women with a history of hypertensive pregnancy disorders at term: A longitudinal follow-up study. BMC Pregnancy and Childbirth 2013;13. | B |
| 1. Hermida RC, Ayala DE, Mojón A, Smolensky MH, Crespo JJ, Otero A, et al. Cardiovascular disease risk stratification by the framigham score is markedly improved by ambulatory compared with office blood pressure. Revista Espanola De Cardiologia 2021;74(11):953-61. | B |
| 1. Hernesniemi JA, Tynkkynen J, Havulinna AS, Oksala N, Vartiainen E, Laatikainen T, Salomaa V. Significant interactions between traditional risk factors affect cardiovascular risk prediction in healthy general population. Annals of Medicine 2015;47(1):53-60. | A |
| 1. Higny J, Dupont M, Guédès A. Cardiac computed tomography in asymptomatic siblings of patients with premature coronary disease: Illustrations and current knowledge. Acta Cardiologica 2020;75(2):107-15. | A |
| 1. Higny J, Dupont M, Guédès A. Cardiac computed tomography in asymptomatic siblings of patients with premature coronary disease: Illustrations and current knowledge. Acta Cardiologica 2020;75(2):107-15. | A |
| 1. Hildrum B, Mykletun A, Dahl AA, Midthjell K. Metabolic syndrome and risk of mortality in middle-aged versus elderly individuals: The nord-trøndelag health study (HUNT). Diabetologia 2009;52(4):583-90. | A |
| 1. Hirai H, Asahi K, Yamaguchi S, Mori H, Satoh H, Iseki K, et al. New risk prediction model of coronary heart disease in participants with and without diabetes: Assessments of the framingham risk and suita scores in 3-year longitudinal database in a japanese population. Scientific Reports 2019;9(1). | D |
| 1. Hirata T, Arai Y, Takayama M, Abe Y, Ohkuma K, Takebayashi T. Carotid plaque score and risk of cardiovascular mortality in the oldest old: Results from the TOOTH study. Journal of Atherosclerosis and Thrombosis 2018;25(1):55-64. | A |
| 1. Hirono A, Kusunose K, Kageyama N, Sumitomo M, Abe M, Fujinaga H, Sata M. Development and validation of optimal cut-off value in inter-arm systolic blood pressure difference for prediction of cardiovascular events. Journal of Cardiology 2018;71(1):24-30. | A |
| 1. Hochholzer W, Valina CM, Stratz C, Amann M, Schlittenhardt D, Büttner HJ, et al. High- sensitivity cardiac troponin for risk prediction in patients with and without coronary heart disease. International Journal of Cardiology 2014;176(2):444-9. | A |
| 1. Hong Y, Jin X, Mo J, Lin HM, Duan Y, Pu M, et al. Metabolic syndrome, its preeminent clusters, incident coronary heart disease and all-cause mortality - results of prospective analysis for the atherosclerosis risk in communities study. Journal of Internal Medicine 2007;262(1):113-23. | A |
| 1. Hopkins PN, Ellison RC, Province MA, Pankow JS, Carr JJ, Arnett DK, et al. Association of coronary artery calcified plaque with clinical coronary heart disease in the national heart, lung, and blood institute's family heart study. American Journal of Cardiology 2006;97(11):1564-9. | C |
| 1. Hou ZH, Lu B, Gao Y, Jiang SL, Wang Y, Li W, Budoff MJ. Prognostic value of coronary CT angiography and calcium score for major adverse cardiac events in outpatients. JACC: Cardiovascular Imaging 2012;5(10):990-9. | A |
| 1. Howard G, Cushman M, Prineas RJ, Howard VJ, Moy CS, Sullivan LM, et al. Advancing the hypothesis that geographic variations in risk factors contribute relatively little to observed geographic variations in heart disease and stroke mortality. Preventive Medicine 2009;49(2-3):129-32. | A |
| 1. Hozawa A, Folsom AR, Sharrett AR, Chambless LE. Absolute and attributable risks of cardiovascular disease incidence in relation to optimal and borderline risk factors: Comparison of african american with white subjects - atherosclerosis risk in communities study. Archives of Internal Medicine 2007;167(6):573-9. | A |
| 1. Hsia J, Rodabough RJ, Manson JE, Liu S, Freiberg MS, Graettinger W, et al. Evaluation of the american heart association cardiovascular disease prevention guideline for women. Circulation: Cardiovascular Quality and Outcomes 2010;3(2):128-34. | B |
| 1. Hsiao LC, Muo CH, Chen YC, Chou CY, Tseng CH, Chang KC. Increased risk of coronary heart disease in patients with chronic osteomyelitis: A population-based study in a cohort of 23 million. Heart 2014;100(18):1450-4. | A |
| 1. Hu D, Jablonski KA, Sparling YH, Robbins DC, Lee ET, Welty TK, Howard BV. Accuracy of lipoprotein lipids and apoproteins in predicting coronary heart disease in diabetic american indians: The strong heart study. Annals of Epidemiology 2002;12(2):79-85. | A |
| 1. Hu G, Tuomilehto J, Borodulin K, Jousilahti P. The joint associations of occupational, commuting, and leisure-time physical activity, and the framingham risk score on the 10-year risk of coronary heart disease. European Heart Journal 2007;28(4):492-8. | B |
| 1. Huang JC, Chen SC, Su HM, Chang JM, Hwang SJ, Chen HC. Performance of the framingham risk score in patients receiving hemodialysis. Nephrology 2013;18(7):510-5. | D |
| 1. Huang JH, Li RH, Huang SL, Sia HK, Yu CH, Tang FC. Gender difference in the relationships between inflammatory markers, serum uric acid and framingham risk score. International Journal of Environmental Research and Public Health 2021;18(13). | B |
| 1. Huang S, Xie X, Sun Y, Zhang T, Cai Y, Xu X, et al. Development of a nomogram that predicts the risk for coronary atherosclerotic heart disease. Aging 2020;12(10):9427-39. | A |
| 1. Huang Z, Dong W. Adversarial MACE prediction after acute coronary syndrome using electronic health records. IEEE Journal of Biomedical and Health Informatics 2019;23(5):2117-26. | A |
| 1. Huerta JM, Gavrila D, Navarro C. Cardiovascular risk estimated after 13 years of follow-up in a low-incidence mediterranean region with high-prevalence of cardiovascular risk factors. BMC Public Health 2010;10. | A |
| 1. Humphries SE, Cooper JA, Talmud PJ, Miller GJ. Candidate gene genotypes, along with conventional risk factor assessment, improve estimation of coronary heart disease risk in healthy UK men. Clinical Chemistry 2007;53(1):8-16. | A |
| 1. Hunt KJ, Sharrett AR, Chambless LE, Folsom AR, Evans GW, Heiss G. Acoustic shadowing on b- mode ultrasound of the carotid artery predicts CHD. Ultrasound in Medicine and Biology 2001;27(3):357-65. | A |
| 1. Hypolite IO, Bucci J, Hshieh P, Cruess D, Agodoa LYC, Yuan CM, et al. Acute coronary syndromes after renal transplantation in patients with end-stage renal disease resulting from diabetes. American Journal of Transplantation 2002;2(3):274-81. | A |
| 1. Icaza G, Núñez L, Marrugat J, Mujica V, Escobar MC, Jiménez AL, et al. Estimation of coronary heart disease risk in chilean subjects based on adapted framingham equations. Revista Medica De Chile 2009;137(10):1273-82. | A |
| 1. Ichikawa K, Miyoshi T, Osawa K, Miki T, Toda H, Ejiri K, et al. Incremental prognostic value of non-alcoholic fatty liver disease over coronary computed tomography angiography findings in patients with suspected coronary artery disease. European Journal of Preventive Cardiology 2021;28(18):2059-66. | D |
| 1. Ingelsson E, Schaefer EJ, Contois JH, McNamara JR, Sullivan L, Keyes MJ, et al. Clinical utility of different lipid measures for prediction of coronary heart disease in men and women. JAMA - Journal of the American Medical Association 2007;298(7):776-85. | A |
| 1. Ingle L, Carroll S, Stamatakis E, Hamer M. Benefit of adding lifestyle-related risk factors for prediction of cardiovascular death among cardiac patients. International Journal of Cardiology 2013;163(2):196-200. | B |
| 1. Ito H, Pacold IV, Durazo-Arvizu R, Liu K, Shilipak MG, Goff Jr DC, et al. The effect of including cystatin C or creatinine in a cardiovascular risk model for asymptomatic individuals. American Journal of Epidemiology 2011;174(8):949-57. | A |
| 1. Ito K, Yoshida H, Yanai H, Kurosawa H, Sato R, Manita D, et al. Relevance of intermediate- density lipoprotein cholesterol to framingham risk score of coronary heart disease in middle-aged men with increased non-hdl cholesterol. International Journal of Cardiology 2013;168(4):3853-8. | A |
| 1. Iwatsuka R, Matsue Y, Yonetsu T, O'Uchi T, Matsumura A, Hashimoto Y, Hirao K. Arterial inflammation measured by 18F-FDG-PET-CT to predict coronary events in older subjects. Atherosclerosis 2018;268:49-54. | D |
| 1. Jacobs PC, Prokop M, van der Graaf Y, Gondrie MJ, Janssen KJ, de Koning HJ, et al. Comparing coronary artery calcium and thoracic aorta calcium for prediction of all-cause mortality and cardiovascular events on low-dose non-gated computed tomography in a high-risk population of heavy smokers. Atherosclerosis 2010;209(2):455-62. | A |
| 1. Janda K, Krzanowski M, Dumnicka P, Kapusta M, Klimeczek P, Chowaniec E, et al. Risk stratification in dialysis patients: Coronary artery calcification score combined with high sensitive c-reactive protein and framingham score for cardiovascular risk prediction in asymptomatic subjects. Journal of Clinical and Experimental Cardiology 2014;5(3). | C |
| 1. Jaquet A, Deloumeaux J, Dumoulin M, Bangou J, Donnet JP, Foucan L. Metabolic syndrome and framingham risk score for prediction of cardiovascular events in caribbean indian patients with blood glucose abnormalities. Diabetes and Metabolism 2008;34(2):177-81. | A |
| 1. Jensen MK, Chiuve SE, Rimm EB, Dethlefsen C, Tjønneland A, Joensen AM, Overvad K. Obesity, behavioral lifestyle factors, and risk of acute coronary events. Circulation 2008;117(24):3062-9. | A |
| 1. Jeong JC, Ro H, Hwang YH, Lee HK, Ha J, Ahn C, Yang J. Cardiovascular diseases after kidney transplantation in korea. Journal of Korean Medical Science 2010;25(11):1589-94. | A |
| 1. Jeppesen J, Hansen TW, Rasmussen S, Ibsen H, Torp-Pedersen C, Madsbad S. Insulin resistance, the metabolic syndrome, and risk of incident cardiovascular disease. A population- based study. Journal of the American College of Cardiology 2007;49(21):2112-9. | C |
| 1. Jöckel KH, Lehmann N, Jaeger BR, Moebus S, Möhlenkamp S, Schmermund A, et al. Smoking cessation and subclinical atherosclerosis-results from the heinz nixdorf recall study. Atherosclerosis 2009;203(1):221-7. | A |
| 1. Jones AF, Walker J, Jewkes C, Game FL, Bartlett WA, Marshall T, Bayly GR. Comparative accuracy of cardiovascular risk prediction methods in primary care patients. Heart 2001;85(1):37-43. | A |
| 1. Jones DW, Chambless LE, Folsom AR, Heiss G, Hutchinson RG, Sharrett AR, et al. Risk factors for coronary heart disease in african americans: The atherosclerosis risk in communities study, 1987-1997. Archives of Internal Medicine 2002;162(22):2565-71. | A |
| 1. Jørgensen PG, Jensen JS, Marott JL, Jensen GB, Appleyard M, Mogelvang R. Electrocardiographic changes improve risk prediction in asymptomatic persons age 65 years or above without cardiovascular disease. Journal of the American College of Cardiology 2014;64(9):898-906. | B |
| 1. Jovičić S, Ignjatović S, Majkić-Singh N. Comparison of two different methods for cardiovascular risk assessment: Framingham risk score and SCORE system. Journal of Medical Biochemistry 2007;26(2):94-7. | B |
| 1. Jung KJ, Jee YH, Jee SH. Metabolic risk score and vascular mortality among korean adults: The korean metabolic syndrome mortality study. Asia-Pacific Journal of Public Health 2017;29(2):122-31. | A |
| 1. Juonala M, Viikari JSA, Räsänen L, Helenius H, Pietikäinen M, Raitakari OT. Young adults with family history of coronary heart disease have increased arterial vulnerability to metabolic risk factors: The cardiovascular risk in young finns study. Arteriosclerosis, Thrombosis, and Vascular Biology 2006;26(6):1376-82. | A |
| 1. Kadota A, Miura K, Okamura T, Fujiyoshi A, Ohkubo T, Kadowaki T, et al. Carotid intima-media thickness and plaque in apparently healthy japanese individuals with an estimated 10-year absolute risk of CAD death according to the japan atherosclerosis society (JAS) guidelines 2012: The shiga epidemiological study of subclinical atherosclerosis (SESSA). Journal of Atherosclerosis and Thrombosis 2013;20(10):755-66. | A |
| 1. Kahan T, Forslund L, Held C, Björkander I, Billing E, Eriksson SV, et al. Risk prediction in stable angina pectoris. European Journal of Clinical Investigation 2013;43(2):141-51. | A |
| 1. Kanjilal S, Rao VS, Mukherjee M, Natesha BK, Renuka KS, Sibi K, et al. Application of cardiovascular disease risk prediction models and the relevance of novel biomarkers to risk stratification in asian indians. Vascular Health and Risk Management 2008;4(1):199-211. | A |
| 1. Kannel WB, Wilson PWF, Nam BH, D'Agostino RB. Risk stratification of obesity as a coronary risk factor. American Journal of Cardiology 2002;90(7):697-701. | A |
| 1. Karalis IK, Alegakis AK, Kafatos AG, Koutis AD, Vardas PE, Lionis CD. Risk factors for ischaemic heart disease in a cretan rural population: A twelve year follow-up study. BMC Public Health 2007;7. | A |
| 1. Karp I, Abrahamowicz M, Bartlett G, Pilote L. Updated risk factor values and the ability of the multivariable risk score to predict coronary heart disease. American Journal of Epidemiology 2004;160(7):707-16. | A |
| 1. Kavousi M, Desai CS, Ayers C, Blumenthal RS, Budoff MJ, Mahabadi AA, et al. Prevalence and prognostic implications of coronary artery calcification in low-risk women: A meta-analysis. JAMA - Journal of the American Medical Association 2016;316(20):2126-34. | A |
| 1. Kavousi M, Elias-Smale S, Rutten JHW, Leening MJG, Vliegenthart R, Verwoert GC, et al. Evaluation of newer risk markers for coronary heart disease risk classification: A cohort study. Annals of Internal Medicine 2012;156(6):438-44. | A |
| 1. Kawai VK, Solus JF, Oeser A, Rho YH, Raggi P, Bian A, et al. Novel cardiovascular risk prediction models in patients with systemic lupus erythematosus. Lupus 2011;20(14):1526-34. | B |
| 1. Kelkar AA, Schultz WM, Khosa F, Schulman-Marcus J, O'Hartaigh BWJ, Gransar H, et al. Long- Term prognosis after coronary artery calcium scoring among low-intermediate risk women and men. Circulation: Cardiovascular Imaging 2016;9(4). | A |
| 1. Kemmler W, Von Stengel S, Bebenek M, Kalender WA. Long-term exercise and risk of metabolic and cardiac diseases: The erlangen fitness and prevention study. Evidence-based Complementary and Alternative Medicine 2013;2013. | A |
| 1. Ken-Dror G, Cooper JA, Humphries SE, Drenos F, Ireland HA. Free protein s level as a risk factor for coronary heart disease and stroke in a prospective cohort study of healthy united kingdom men. American Journal of Epidemiology 2011;174(8):958-68. | A |
| 1. Kengne AP, Patel A, Colagiuri S, Heller S, Hamet P, Marre M, et al. The framingham and UK prospective diabetes study (UKPDS) risk equations do not reliably estimate the probability of cardiovascular events in a large ethnically diverse sample of patients with diabetes: The action in diabetes and vascular disease: Preterax and diamicron-mr controlled evaluation (ADVANCE) study. Diabetologia 2010;53(5):821-31. | B |
| 1. Ketlogetswe K, Blumenthal RS. Thirty-year multivariate risk assessment is a stronger predictor of cardiovascular disease than the 10-year model. Clinical Chemistry 2009;55(12):2085-7. | A |
| 1. Khaleeli E, Peters SR, Bobrowsky K, Oudiz RJ, Ko JY, Budoff MJ. Diabetes and the associated incidence of subclinical atherosclerosis and coronary artery disease: Implications for management. American Heart Journal 2001;141(4):637-44.q | A |
| 1. Khalili D, Hadaegh F, Soori H, Steyerberg EW, Bozorgmanesh M, Azizi F. Clinical usefulness of the framingham cardiovascular risk profile beyond its statistical performance. American Journal of Epidemiology 2012;176(3):177-86. | B |
| 1. Khalili S, Hatami M, Hadaegh F, Sheikholeslami F, Azizi F. Prediction of cardiovascular events with consideration of general and central obesity measures in diabetic adults: Results of the 8.4- year follow-up. Metabolic Syndrome and Related Disorders 2012;10(3):218-24. | A |
| 1. Khandaker MH, Miller TD, Chareonthaitawee P, Wells Askew J, Hodge DO, Gibbons RJ. Stress single photon emission computed tomography for detection of coronary artery disease and risk stratification of asymptomatic patients at moderate risk. Journal of Nuclear Cardiology 2009;16(4):516-23. | A |
| 1. Kimball AB, Guerin A, Latremouille-Viau D, Yu AP, Gupta S, Bao Y, Mulani P. Coronary heart disease and stroke risk in patients with psoriasis: Retrospective analysis. American Journal of Medicine 2010;123(4):350-7. | A |
| 1. Kip KE, Marroquin OC, Shaw LJ, Arant CB, Wessel TR, Olson MB, et al. Global inflammation predicts cardiovascular risk in women: A report from the women's ischemia syndrome evaluation (WISE) study. American Heart Journal 2005;150(5):900-6. | A |
| 1. Kivimäki M, David Batty G, Hamer M, Ferrie JE, Vahtera J, Virtanen M, et al. Using additional information on working hours to predict coronary heart disease. Annals of Internal Medicine 2011;154(7):457-63. | A |
| 1. Kivimäki M, Nyberg ST, Batty GD, Shipley MJ, Ferrie JE, Virtanen M, et al. Does adding information on job strain improve risk prediction for coronary heart disease beyond the standard framingham risk score? The whitehall ii study. International Journal of Epidemiology 2011;40(6):1577-84. | A |
| 1. Knuiman MW, Hung J, Divitini ML, Davis TM, Beilby JP. Utility of the metabolic syndrome and its components in the prediction of incident cardiovascular disease: Aprospective cohort study. European Journal of Cardiovascular Prevention and Rehabilitation 2009;16(2):235-41. | A |
| 1. Ko DT, Sivaswamy A, Sud M, Kotrri G, Azizi P, Koh M, et al. Calibration and discrimination of the framingham risk score and the pooled cohort equations. CMAJ 2020;192(17):E442-9. | C |
| 1. Koller MT, Leening MJG, Wolbers M, Steyerberg EW, Myriam Hunink MG, Schoop R, et al. Development and validation of a coronary risk prediction model for older U.S. And european persons in the cardiovascular health study and the rotterdam study. Annals of Internal Medicine 2012;157(6):389-97. | B |
| 1. Koller MT, Steyerberg EW, Wolbers M, Stijnen T, Bucher HC, Hunink MGM, Witteman JCM. Validity of the framingham point scores in the elderly: Results from the rotterdam study. American Heart Journal 2007;154(1):87-93. | B |
| 1. Konety SH, Koene RJ, Norby FL, Wilsdon T, Alonso A, Siscovick D, et al. Echocardiographic predictors of sudden cardiac death. Circulation: Cardiovascular Imaging 2016;9(8). | C |
| 1. Konno S, Munakata M. Moderately increased albuminuria is an independent risk factor of cardiovascular events in the general japanese population under 75 years of age: The watari study. PLoS ONE 2015;10(4). | A |
| 1. Kral BG, Becker DM, Vaidya D, Yanek LR, Becker LC. Severity of inducible myocardial ischemia predicts incident acute coronary syndromes in asymptomatic individuals with a family history of premature coronary artery disease. Journal of Nuclear Cardiology 2012;19(1):28-36. | A |
| 1. Kral BG, Becker LC, Vaidya D, Yanek LR, Becker DM. Silent myocardial ischaemia and long- term coronary artery disease outcomes in apparently healthy people from families with early- onset ischaemic heart disease. European Heart Journal 2011;32(22):2766-72. | A |
| 1. Kral BG, Mathias RA, Suktitipat B, Ruczinski I, Vaidya D, Yanek LR, et al. A common variant in the CDKN2B gene on chromosome 9p21 protects against coronary artery disease in americans of african ancestry. Journal of Human Genetics 2011;56(3):224-9. | A |
| 1. Krikke M, Hoogeveen R, Hoepelman A, Visseren F, Arends JE. Cardiovascular risk prediction in hiv-infected patients: Comparing the framingham, atherosclerotic cardiovascular disease risk score (ASCVD), systematic coronary risk evaluation for the netherlands (SCORE-NL) and data collection on adverse events of anti-hiv drugs (D: A: D) risk prediction models. HIV Medicine 2016;17(4):289-97. | A |
| 1. Krohn JB, Neubauer C, Fischer S, Oberkanins C, Katus HA, Gleissner CA. Optimisation of individual cardiovascular risk assessment in a german coronary artery disease cohort using a commercial test for genetic polymorphisms–a pilot study. Acta Cardiologica 2022. | A |
| 1. Kullo IJ, Jouni H, Austin EE, Brown SA, Kruisselbrink TM, Isseh IN, et al. Incorporating a genetic risk score into coronary heart disease risk estimates: Effect on low-density lipoprotein cholesterol levels (the MI-GENES clinical trial). Circulation 2016;133(12):1181-8. | A |
| 1. Kumar A, Prineas RJ, Arnold AM, Psaty BM, Furberg CD, Robbins J, Lloyd-Jones DM. Prevalence, prognosis, and implications of isolated minor nonspecific st-segment and t-wave abnormalities in older adults cardiovascular health study. Circulation 2008;118(25):2790-6. | A |
| 1. Kuo CF, Yu KH, See LC, Chou IJ, Ko YS, Chang HC, et al. Risk of myocardial infarction among patients with gout: A nationwide population-based study. Rheumatology 2013;52(1):111-7. | A |
| 1. L'Italien G, Ford I, Norrie J, Lapuerta P, Ehreth J, Jackson J, Shepherd J. The cardiovascular event reduction tool (CERT) - A simplified cardiac risk prediction model developed from the west of scotland coronary prevention study (WOSCOPS). American Journal of Cardiology 2000;85(6):720-4. | A |
| 1. Lau KK, Chan YH, Yiu KH, Tam S, Li SW, Lau CP, Tse HF. Incremental predictive value of vascular assessments combined with the framingham risk score for prediction of coronary events in subjects of low-intermediate risk. Postgraduate Medical Journal 2008;84(989):153-7. | A |
| 1. Law M, Friis-Möller N, Weber R, Reiss P, Thiebaut R, Kirk O, et al. Modelling the 3-year risk of myocardial infarction among participants in the data collection of adverse events of anti-hiv drugs (DAD) study. HIV Medicine 2003;4(1):1-10. | A |
| 1. Law MG, Friis-Møller N, El-Sadr WM, Weber R, Reiss P, D'Arminio Monforte A, et al. The use of the framingham equation to predict myocardial infarctions in hiv-infected patients: Comparison with observed events in the D:A:D study. HIV Medicine 2006;7(4):218-30. | B |
| 1. Lee AJ, Price JF, Russell MJ, Smith FB, Van Wijk MCW, Fowkes FGR. Improved prediction of fatal myocardial infarction using the ankle brachial index in addition to conventional risk factors: The edinburgh artery study. Circulation 2004;110(19):3075-80. | A |
| 1. Lee BC, Lee WJ, Lo SC, Hsu HC, Chien KL, Chang YC, Chen MF. The ratio of epicardial to body fat improves the prediction of coronary artery disease beyond calcium and framingham risk scores. International Journal of Cardiovascular Imaging 2016;32:117-27. | A |
| 1. Lee CD, Folsom AR, Pankow JS, Brancati FL. Cardiovascular events in diabetic and nondiabetic adults with or without history of myocardial infarction. Circulation 2004;109(7):855-60. | A |
| 1. Lee ET, Howard BV, Wang W, Welty TK, Galloway JM, Best LG, et al. Prediction of coronary heart disease in a population with high prevalence of diabetes and albuminuria: The strong heart study. Circulation 2006;113(25):2897-905. | A |
| 1. Lee J, Heng D, Chia KS, Chew SK, Tan BY, Hughes K. Risk factors and incident coronary heart disease in chinese, malay and asian indian males: The singapore cardiovascular cohort study. International Journal of Epidemiology 2001;30(5):983-8. | A |
| 1. Lee J, Heng D, Ma S, Chew SK, Hughes K, Tai ES. The metabolic syndrome and mortality: The singapore cardiovascular cohort study. Clinical Endocrinology 2008;69(2):225-30. | C |
| 1. Lee JH, Rizvi A, Hartaigh B, Han D, Park MW, Roudsari HM, et al. The predictive value of coronary artery calcium scoring for major adverse cardiac events according to renal function (from the coronary computed tomography angiography evaluation for clinical outcomes: An international multicenter [CONFIRM] registry). American Journal of Cardiology 2019;123(9):1435-42. | D |
| 1. Lee K. 10-year risk for atherosclerotic cardiovascular disease and coronary heart disease among korean adults: Findings from the korean national health and nutrition examination survey 2009-2010. International Journal of Cardiology 2014;176(2):418-22. | A |
| 1. Lehmann N, Erbel R, Mahabadi AA, Rauwolf M, Möhlenkamp S, Moebus S, et al. Value of progression of coronary artery calcification for risk prediction of coronary and cardiovascular events: Result of the hnr study (heinz nixdorf recall). Circulation 2018;137(7):665-79. | A |
| 1. Lehmann N, Möhlenkamp S, Mahabadi AA, Schmermund A, Roggenbuck U, Seibel R, et al. Effect of smoking and other traditional risk factors on the onset of coronary artery calcification: Results of the heinz nixdorf recall study. Atherosclerosis 2014;232(2):339-45. | C |
| 1. Lenselink C, Ties D, Pleijhuis R, Van Der Harst P. Validation and comparison of 28 risk prediction models for coronary artery disease. European Journal of Preventive Cardiology 2022;29(4):666-74. | D |
| 1. Li C, Engström G, Hedblad B, Janzon L. Sex-specific cardiovascular morbidity and mortality in a cohort treated for hypertension. Journal of Hypertension 2006;24(8):1523-9. | A |
| 1. Li H, Cui Y, Zhu Y, Yan H, Xu W. Association of high normal hba1c and TSH levels with the risk of CHD: A 10-year cohort study and SVM analysis. Scientific Reports 2017;7. | A |
| 1. Li TC, Wang HC, Li CI, Liu CS, Lin WY, Lin CH, et al. Establishment and validation of a prediction model for ischemic stroke risks in patients with type 2 diabetes. Diabetes Research and Clinical Practice 2018;138:220-8. | A |
| 1. Liao KP, Ananthakrishnan AN, Kumar V, Xia Z, Cagan A, Gainer VS, et al. Methods to develop an electronic medical record phenotype algorithm to compare the risk of coronary artery disease across 3 chronic disease cohorts. PLoS ONE 2015;10(8). | A |
| 1. Liao Y, Hawthorne V, Kozarevic D, Vojvodic N, Gillis C, Hole D, et al. Prediction of mortality from coronary heart disease among diverse populations: Is there a common predictive function? Heart 2002;88(3):222-8. | A |
| 1. Liao Y, McGee DL, Cooper RS, Sutkowski MBE. How generalizable are coronary risk prediction models? Comparison of framingham and two national cohorts. American Heart Journal 1999;137(5):837-45. | A |
| 1. Liao Y, McGee DL, Cooper RS. Prediction of coronary heart disease mortality in blacks and whites: Pooled data from two national cohorts. American Journal of Cardiology 1999;84(1):31-6. | A |
| 1. Lima-Costa MF, Cesar CC, Chor D, Proietti FA. Self-rated health compared with objectively measured health status as a tool for mortality risk screening in older adults: 10-year follow-up of the bambuí cohort study of aging. American Journal of Epidemiology 2012;175(3):228-35. | A |
| 1. Liu J, Hong Y, D'Agostino Sr RB, Wu Z, Wang W, Sun J, et al. Predictive value for the chinese population of the framingham CHD risk assessment tool compared with the chinese multi- provincial cohort study. JAMA - Journal of the American Medical Association 2004;291(21):2591-9. | B |
| 1. Liu L, Tang Z, Li X, Luo Y, Guo J, Li H, et al. A novel risk score to the prediction of 10-year risk for coronary artery disease among the elderly in beijing based on competing risk model. Medicine 2016;95(11). | A |
| 1. Lloyd-Jones DM, Nam BH, D'Agostino Sr RB, Levy D, Murabito JM, Wang TJ, et al. Parental cardiovascular disease as a risk factor for cardiovascular disease in middle-aged adults: A prospective study of parents and offspring. JAMA - Journal of the American Medical Association 2004;291(18):2204-11. | A |
| 1. Lloyd-Jones DM, Wilson PWF, Larson MG, Beiser A, Leip EP, D'Agostino RB, Levy D. Framingham risk score and prediction of lifetime risk for coronary heart disease. American Journal of Cardiology 2004;94(1):20-4. | A |
| 1. Lloyd-Jones DM, Wilson PWF, Larson MG, Leip E, Beiser A, D'Agostino RB, et al. Lifetime risk of coronary heart disease by cholesterol levels at selected ages. Archives of Internal Medicine 2003;163(16):1966-72. | A |
| 1. Lluis-Ganella C, Subirana I, Lucas G, Tomás M, Muñoz D, Sentí M, et al. Assessment of the value of a genetic risk score in improving the estimation of coronary risk. Atherosclerosis 2012;222(2):456-63. | A |
| 1. Lo KY, Leung KF, Chu CM, Loke KL, Chan CK, Yue CS. Prognostic value of adenosine stress myocardial perfusion by cardiac magnetic resonance imaging in patients with known or suspected coronary artery disease. QJM 2011;104(5):425-32. | A |
| 1. Łopuszańska M, Szklarska A, Lipowicz A, Jankowska EA, Kozieł S. Life satisfaction and cardiovascular disease risk in poland. Archives of Medical Science 2013;9(4):629-34. | A |
| 1. Lu SE, Beckles GL, Crosson JC, Bilik D, Karter AJ, Gerzoff RB, et al. Evaluation of risk equations for prediction of short-term coronary heart disease events in patients with long- standing type 2 diabetes: The translating research into action for diabetes (TRIAD) study. BMC Endocrine Disorders 2012;12. | D |
| 1. Lumey LH, Martini LH, Myerson M, Stein AD, Prineas RJ. No relation between coronary artery disease or electrocardiographic markers of disease in middle age and prenatal exposure to the dutch famine of 1944-5. Heart 2012;98(22):1653-9. | A |
| 1. Lundberg C, Johansson L, Barbier CE, Lind L, Ahlström H, Hansen T. Total atherosclerotic burden by whole body magnetic resonance angiography predicts major adverse cardiovascular events. Atherosclerosis 2013;228(1):148-52. | D |
| 1. Lyngbæk S, Marott JL, Sehestedt T, Hansen TW, Olsen MH, Andersen O, et al. Cardiovascular risk prediction in the general population with use of supar, CRP, and framingham risk score. International Journal of Cardiology 2013;167(6):2904-11. | C |
| 1. Ma CP, Wang X, Wang QS, Liu XL, He XN, Nie SP. A modified HEART risk score in chest pain patients with suspected non-st-segment elevation acute coronary syndrome. Journal of Geriatric Cardiology 2016;13(1):64-9. | A |
| 1. Mackness B, Durrington P, McElduff P, Yarnell J, Azam N, Watt M, Mackness M. Low paraoxonase activity predicts coronary events in the caerphilly prospective study. Circulation 2003;107(22):2775-9. | A |
| 1. Magri CJ, Debono R, Calleja N, Galea J, Fava S. Prognostic indicators and generation of novel risk equations for estimation of 10-year and 20-year mortality following acute coronary syndrome. Postgraduate Medical Journal 2017;93(1099):245-9. | A |
| 1. Mainous Iii AG, Everett CJ, Player MS, King DE, Diaz VA. Importance of a patient's personal health history on assessments of future risk of coronary heart disease. Journal of the American Board of Family Medicine 2008;21(5):408-13. | B |
| 1. Majed B, Tafflet M, Kee F, Haas B, Ferrieres J, Montaye M, et al. External validation of the 2008 framingham cardiovascular risk equation for CHD and stroke events in a european population of middle-aged men. The PRIME study. Preventive Medicine 2013;57(1):49-54. | B |
| 1. Malik S, Zhao Y, Budoff M, Nasir K, Blumenthal RS, Bertoni AG, Wong ND. Coronary artery calcium score for long-term risk classification in individuals with type 2 diabetes and metabolic syndrome from the multi-ethnic study of atherosclerosis. JAMA Cardiology 2017;2(12):1332-40.q | A |
| 1. Mallat Z, Benessiano J, Simon T, Ederhy S, Sebella-Arguelles C, Cohen A, et al. Circulating secretory phospholipase A2 activity and risk of incident coronary events in healthy men and women: The EPIC-NORFOLK study. Arteriosclerosis, Thrombosis, and Vascular Biology 2007;27(5):1177-83. | A |
| 1. Mannan H, Stevenson C, Peeters A, Walls H, McNeil J. Framingham risk prediction equations for incidence of cardiovascular disease using detailed measures for smoking. Heart International 2010;5(2):49-57. | A |
| 1. Mansell H, Stewart SA, Shoker A. Validity of cardiovascular risk prediction models in kidney transplant recipients. The Scientific World Journal 2014;2014. | A |
| 1. Maracy MR, Hosseini SM, Amini M. Risk scores in ischaemic heart disease patients with type 2 diabetes, isfahan, iran. Acta Cardiologica 2008;63(6):729-34. | A |
| 1. Marchioli R, Avanzini F, Barzi F, Chieffo C, Di Castelnuovo A, Franzosi MG, et al. Assessment of absolute risk of death after myocardial infarction by use of multiple-risk-factor assessment equations: GISSI-prevenzione mortality risk chart. European Heart Journal 2001;22(22):2085-103. | A |
| 1. Marma AK, Lloyd-Jones DM. Systematic examination of the updated framingham heart study general cardiovascular risk profile. Circulation 2009;120(5):384-90. | A |
| 1. Marques-Vidal P, Rodondi N, Bochud M, Chiolero A, Pécoud A, Hayoz D, et al. Predictive accuracy of original and recalibrated framingham risk score in the swiss population. International Journal of Cardiology 2009;133(3):346-53. | A |
| 1. Marrugat J, D'Agostino R, Sullivan L, Elosua R, Wilson P, Ordovas J, et al. An adaptation of the framingham coronary heart disease risk function to european mediterranean areas. Journal of Epidemiology and Community Health 2003;57(8):634-8. | B |
| 1. Marrugat J, Solanas P, D'Agostino R, Sullivan L, Ordovas J, Cordón F, et al. Coronary risk estimation in spain using a calibrated framingham function. Revista Espanola De Cardiologia 2003;56(3):253-61. | A |
| 1. Marrugat J, Subirana I, Ramos R, Vila J, Marín-Ibañez A, Guembe MJ, et al. Derivation and validation of a set of 10-year cardiovascular risk predictive functions in spain: The FRESCO study. Preventive Medicine 2014;61:66-74. | B |
| 1. Marschner IC, Colquhoun D, John Simes R, Glasziou P, Harris P, Singh BB, et al. Long-term risk stratification for survivors of acute coronary syndromes: Results from the long-term intervention with pravastatin in ischemic disease (LIPID) study. Journal of the American College of Cardiology 2001;38(1):56-63. | A |
| 1. Matsubara Y, Kimachi M, Fukuma S, Onishi Y, Fukuhara S. Development of a new risk model for predicting cardiovascular events among hemodialysis patients: Population-based hemodialysis patients from the japan dialysis outcome and practice patterns study (JDOPPS). PLoS ONE 2017;12(3). | D |
| 1. Matsumoto M, Ishikawa S, Kayaba K, Gotoh T, Nago N, Tsutsumi A, Kajii E. Risk charts illustrating the 10-year risk of myocardial infarction among residents of japanese rural communities: The JMS cohort study. Journal of Epidemiology 2009;19(2):94-100. | A |
| 1. McClelland RL, Jorgensen NW, Budoff M, Blaha MJ, Post WS, Kronmal RA, et al. 10-Year coronary heart disease risk prediction using coronary artery calcium and traditional risk factors derivation in the MESA (multi-ethnic study of atherosclerosis) with validation in the HNR (heinz nixdorf recall) study and the DHS (dallas heart study). Journal of the American College of Cardiology 2015;66(15):1643-53. | A |
| 1. McEvoy JW, Blaha MJ, Rivera JJ, Budoff MJ, Khan AN, Shaw LJ, et al. Mortality rates in smokers and nonsmokers in the presence or absence of coronary artery calcification. JACC: Cardiovascular Imaging 2012;5(10):1037-45. | A |
| 1. McGeechan K, Liew G, Macaskill P, Irwig L, Klein R, Klein BEK, et al. Meta-analysis: Retinal vessel caliber and risk for coronary heart disease. Annals of Internal Medicine 2009;151(6):404-13. | A |
| 1. McGorrian C, Yusuf S, Islam S, Jung H, Rangarajan S, Avezum A, et al. Estimating modifiable coronary heart disease risk in multiple regions of the world: The INTERHEART modifiable risk score. European Heart Journal 2011;32(5):581-90. | A |
| 1. McKnight J. Predicting vascular risk in type 1 diabetes: Stratification in a hospital based population in scotland. Diabetic Medicine 2005;22(2):164-71. | A |
| 1. Meigs JB, Larson MG, D'Agostino RB, Levy D, Clouse ME, Nathan DM, et al. Coronary artery calcification in type 2 diabetes and insulin resistance: The framingham offspring study. Diabetes Care 2002;25(8):1313-9. | A |
| 1. Meisinger C, Loewel H, Mraz W, Koenig W. Prognostic value of apolipoprotein B and A-I in the prediction of myocardial infarction in middle-aged men and women: Results from the MONICA/ KORA augsburg cohort study. European Heart Journal 2005;26(3):271-8. | A |
| 1. Mendivil CO, Rimm EB, Furtado J, Chiuve SE, Sacks FM. Low-density lipoproteins containing apolipoprotein C-III and the risk of coronary heart disease. Circulation 2011;124(19):2065-72. | A |
| 1. Menotti A, Lanti M, Agabiti-Rosei E, Carratelli L, Cavera G, Dormi A, et al. Riskard 2005. New tools for prediction of cardiovascular disease risk derived from italian population studies. Nutrition, Metabolism and Cardiovascular Diseases 2005;15(6):426-40. | A |
| 1. Menotti A, Lanti M. An italian chart for cardiovascular risk estimate including high-density lipoprotein-cholesterol. Disease Management and Health Outcomes 2008;16(3):183-97. | A |
| 1. Merino J, Planas A, De Moner A, Gasol A, Contreras C, Marrugat J, et al. The association of peripheral arterial occlusive disease with major coronary events in a mediterranean population with low coronary heart disease incidence. European Journal of Vascular and Endovascular Surgery 2008;36(1):71-6. | A |
| 1. Merry AHH, Boer JMA, Schouten LJ, Feskens EJM, Verschuren WM, Gorgels APM, van den Brandt PA. Smoking, alcohol consumption, physical activity, and family history and the risks of acute myocardial infarction and unstable angina pectoris: A prospective cohort study. BMC Cardiovascular Disorders 2011;11. | A |
| 1. Miller TD, Roger VL, Hodge DO, Gibbons RJ. A simple clinical score accurately predicts outcome in a community-based population undergoing stress testing. American Journal of Medicine 2005;118(8):866-72. | A |
| 1. Miname M, Bensenor IM, Lotufo PA. Different methods of calculating ankle-brachial index in mid-elderly men and women: The brazilian longitudinal study of adult health (elsa-brasil). Brazilian Journal of Medical and Biological Research 2016;49(12). | A |
| 1. Minoura A, Wang DH, Sato Y, Zou Y, Sakano N, Kubo M, et al. Association of dietary fat and carbohydrate consumption and predicted ten-year risk for developing coronary heart disease in a general japanese population. Acta Medica Okayama 2014;68(3):129-35. | A |
| 1. Mladenov V, Mastorakis N, Bulucea A, editors. Online adaptive coronary heart disease risk prediction model; 21st international conference on circuits, systems, communications and computers, CSCC 2017. EDP Sciences; 2017ak. | A |
| 1. Möller CS, Zethelius B, Sundström J, Lind L. Persistent ischaemic ECG abnormalities on repeated ECG examination have important prognostic value for cardiovascular disease beyond established risk factors: A population-based study in middle-aged men with up to 32 years of follow-up. Heart 2007;93(9):1104-10. | C |
| 1. Moon SJ, Chun EJ, Yoon YE, Park KS, Jang HC, Lim S. Long-Term prognostic value of coronary computed tomography angiography in an asymptomatic elderly population. Journal of the American Heart Association 2019;8(23). | B |
| 1. Moorman AJ, Dean LS, Yang E, Drezner JA. Cardiovascular risk assessment in the older athlete. Sports Health 2021;13(6):622-9. | A |
| 1. Mora S, Redberg RF, Cui Y, Whiteman MK, Flaws JA, Sharrett AR, Blumenthal RS. Ability of exercise testing to predict cardiovascular and all-cause death in asymptomatic women: A 20-year follow-up of the lipid research clinics prevalence study. JAMA - Journal of the American Medical Association 2003;290(12):1600-7. | C |
| 1. Moreno PR. Prediction of MACE after ACS: Demographics and angiography versus imaging. JACC: Cardiovascular Imaging 2013;6(12):1273-6. | A |
| 1. Morieri ML, Gao H, Pigeyre M, Shah HS, Sjaarda J, Mendonca C, et al. Genetic tools for coronary risk assessment in type 2 diabetes: A cohort study from the ACCORD clinical trial. Diabetes Care 2018;41(11):2404-13. | A |
| 1. Morrison AC, Bare LA, Chambless LE, Ellis SG, Malloy M, Kane JP, et al. Prediction of coronary heart disease risk using a genetic risk score: The atherosclerosis risk in communities study. American Journal of Epidemiology 2007;166(1):28-35. | A |
| 1. Mostofsky E, MacLure M, Sherwood JB, Tofler GH, Muller JE, Mittleman MA. Risk of acute myocardial infarction after the death of a significant person in one's life: The determinants of myocardial infarction onset study. Circulation 2012;125(3):491-6. | A |
| 1. Motwani M, Dey D, Berman DS, Germano G, Achenbach S, Al-Mallah MH, et al. Machine learning for prediction of all-cause mortality in patients with suspected coronary artery disease: A 5-year multicentre prospective registry analysis. European Heart Journal 2017;38(7):500-7. | C |
| 1. Mozaffarian D, Kamineni A, Prineas RJ, Siscovick DS. Metabolic syndrome and mortality in older adults: The cardiovascular health study. Archives of Internal Medicine 2008;168(9):969-78. | A |
| 1. Mühlenbruch K, Ludwig T, Jeppesen C, Joost HG, Rathmann W, Meisinger C, et al. Update of the german diabetes risk score and external validation in the german MONICA/KORA study. Diabetes Research and Clinical Practice 2014;104(3):459-66. | A |
| 1. Mukamal KJ, Kizer JR, Djoussé L, Ix JH, Zieman S, Siscovick DS, et al. Prediction and classification of cardiovascular disease risk in older adults with diabetes. Diabetologia 2013;56(2):275-83. | A |
| 1. Nakanishi K, Fukuda S, Shimada K, Ehara S, Inanami H, Matsumoto K, et al. Non-obstructive low attenuation coronary plaque predicts three-year acute coronary syndrome events in patients with hypertension: Multidetector computed tomographic study. Journal of Cardiology 2012;59(2):167-75. | D |
| 1. Nance Jr JW, Schlett CL, Schoepf UJ, Oberoi S, Leisy HB, Barraza Jr JM, et al. Incremental prognostic value of different components of coronary atherosclerotic plaque at cardiac CT angiography beyond coronary calcification in patients with acute chest pain. Radiology 2012;264(3):679-90. | A |
| 1. Nargesi AA, Esteghamati S, Heidari B, Hafezi-Nejad N, Sheikhbahaei S, Pajouhi A, et al. Nonlinear relation between pulse pressure and coronary heart disease in patients with type 2 diabetes or hypertension. Journal of Hypertension 2016;34(5):974-80. | A |
| 1. Nasir K, Vasamreddy C, Blumenthal RS, Rumberger JA. Comprehensive coronary risk determination in primary prevention: An imaging and clinical based definition combining computed tomographic coronary artery calcium score and national cholesterol education program risk score. International Journal of Cardiology 2006;110(2):129-36. | A |
| 1. Nassef Y, Lee KJ, Nfor ON, Tantoh DM, Chou MC, Liaw YP. The impact of aerobic exercise and badminton on hdl cholesterol levels in taiwanese adults. Nutrients 2020;12(5). | B |
| 1. Navas-Nacher EL, Colangelo L, Beam C, Greenland P. Risk factors for coronary heart disease in men 18 to 39 years of age. Annals of Internal Medicine 2001;134(6):433-9. | A |
| 1. Negi SI, Nambi V. The role of carotid intimal thickness and plaque imaging in risk stratification for coronary heart disease. Current Atherosclerosis Reports 2012;14(2):115-23. | A |
| 1. Neuhauser HK, Ellert U, Kurth BM. A comparison of framingham and score-based cardiovascular risk estimates in participants of the german national health interview and examination survey 1998. European Journal of Cardiovascular Prevention and Rehabilitation 2005;12(5):442-50. | B |
| 1. Newman JD, Davidson KW, Shaffer JA, Schwartz JE, Chaplin W, Kirkland S, Shimbo D. Observed hostility and the risk of incident ischemic heart disease: A prospective population study from the 1995 canadian nova scotia health survey. Journal of the American College of Cardiology 2011;58(12):1222-8. | A |
| 1. Nielsen M, Ganz M, Lauze F, Pettersen PC, de Bruijne M, Clarkson TB, et al. Distribution, size, shape, growth potential and extent of abdominal aortic calcified deposits predict mortality in postmenopausal women. BMC Cardiovascular Disorders 2010;10. | C |
| 1. Nigam A, Bourassa MG, Fortier A, Guertin MC, Tardif JC. The metabolic syndrome and its components and the long-term risk of death in patients with coronary heart disease. American Heart Journal 2006;151(2):514-21. | A |
| 1. Nikpour M, Gladman DD, Ibañez D, Bruce IN, Burns RJ, Urowitz MB. Myocardial perfusion imaging in assessing risk of coronary events in patients with systemic lupus erythematosus. Journal of Rheumatology 2009;36(2):288-94. | A |
| 1. Nishimoto M, Tagawa M, Matsui M, Eriguchi M, Samejima KI, Iseki K, et al. A prediction model with lifestyle in addition to previously known risk factors improves its predictive ability for cardiovascular death. Scientific Reports 2019;9(1). | A |
| 1. Noda H, Maruyama K, Iso H, Dohi S, Terai T, Fujioka S, et al. Prediction of myocardial infarction using coronary risk scores among japanese male workers: 3M study. Journal of Atherosclerosis and Thrombosis 2010;17(5):452-9. | A |
| 1. Noto D, Cefalù AB, Barbagallo CM, Sapienza M, Cavera G, Nardi I, et al. Hypertension and diabetes mellitus are associated with cardiovascular events in the elderly without cardiovascular disease. Results of a 15-year follow-up in a mediterranean population. Nutrition, Metabolism and Cardiovascular Diseases 2009;19(5):321-6. | A |
| 1. Novo S, Carità P, Lo Voi A, Muratori I, Tantillo R, Corrado E, et al. Impact of preclinical carotid atherosclerosis on global cardiovascular risk stratification and events in a 10-year follow-up: Comparison between the algorithms of the framingham heart study, the european SCORE and the italian ‘progetto cuore’. Journal of Cardiovascular Medicine 2019;20(2):91-6. | B |
| 1. Novo S, Visconti CL, Amoroso GR, Corrado E, Fazio G, Muratori I, et al. Asymptomatic carotid lesions add to cardiovascular risk prediction. European Journal of Cardiovascular Prevention and Rehabilitation 2010;17(5):514-8. | A |
| 1. Nucifora G, Schuijf JD, van Werkhoven JM, Jukema JW, Djaberi R, Scholte AJHA, et al. Prevalence of coronary artery disease across the framingham risk categories: Coronary artery calcium scoring and MSCT coronary angiography. Journal of Nuclear Cardiology 2009;16(3):368-75. | A |
| 1. O'Connor SD, Graffy PM, Zea R, Pickhardt PJ. Does nonenhanced ct-based quantification of abdominal aortic calcification outperform the framingham risk score in predicting cardiovascular events in asymptomatic adults? Radiology 2019;290(1):108-15. | A |
| 1. Ogino I, Watanabe S, Iwahashi N, Kosuge M, Sakamaki K, Kunisaki C, Kimura K. Symptomatic radiation-induced cardiac disease in long-term survivors of esophageal cancer. Strahlentherapie Und Onkologie 2016;192(6):359-67. | A |
| 1. Okayama KI, Mita T, Gosho M, Yamamoto R, Yoshida M, Kanazawa A, et al. Carotid intima- media thickness progression predicts cardiovascular events in japanese patients with type 2 diabetes. Diabetes Research and Clinical Practice 2013;101(3):286-92. | A |
| 1. Okwuosa TM, Greenland P, Burke GL, Eng J, Cushman M, Michos ED, et al. Prediction of coronary artery calcium progression in individuals with low framingham risk score: The multi- ethnic study of atherosclerosis. JACC: Cardiovascular Imaging 2012;5(2):144-53. | A |
| 1. Okwuosa TM, Greenland P, Ning H, Liu K, Lloyd-Jones DM. Yield of screening for coronary artery calcium in early middle-age adults based on the 10-year framingham risk score: The CARDIA study. JACC: Cardiovascular Imaging 2012;5(9):923-30. | B |
| 1. Olesnevich ME, Kuczmarski MF, Mason M, Fang C, Zonderman AB, Evans MK. Serum ferritin levels associated with increased risk for developing CHD in a low-income urban population. Public Health Nutrition 2012;15(7):1291-8. | A |
| 1. on behalf of the DADSG, Ryom L, Rickenbach M, Sabin CA, El-Sadr W, d'Arminio Monforte A, et al. Increased risk of cardiovascular disease (CVD) with age in hiv-positive men: A comparison of the D: A: D CVD risk equation and general population CVD risk equations. HIV Medicine 2014;15(10):595-603. | A |
| 1. Pai JK, Pischon T, Ma J, Manson JE, Hankinson SE, Joshipura K, et al. Inflammatory markers and the risk of coronary heart disease in men and women. New England Journal of Medicine 2004;351(25):2599-610. | A |
| 1. Palladino R, Caporale O, Nardone A, Fiorentino D, Torre I, Triassi M. Use of framingham risk score as a clinical tool for the assessment of fitness for work: Results from a cohort study. Journal of Occupational and Environmental Medicine 2016;58(8):805-9. | B |
| 1. Palmieri L, Panico S, Vanuzzo D, Ferrario M, Pilotto L, Sega R, et al. Evaluation of the global cardiovascular absolute risk: The progetto CUORE individual score. Annali Dell'Istituto Superiore Di Sanita 2004;40(4):393-9. | A |
| 1. Panagiotakos DB, Fitzgerald AP, Pitsavos C, Pipilis A, Graham I, Stefanadis C. Statistical modelling of 10-year fatal cardiovascular disease risk in greece: The hellenicscore (a calibration of the ESC SCORE project). Hellenic Journal of Cardiology 2007;48(2):55-63. | A |
| 1. Panagiotakos DB, Magriplis E, Zampelas A, Mitsopoulou AV, Karageorgou D, Dimakopoulos I, et al. The recalibrated hellenicscore based on newly derived risk factors from the hellenic national nutrition and health survey (HNNHS); the hellenicscore II. Hellenic Journal of Cardiology 2021;62(4):285-90. | A |
| 1. Panagiotakos DB, Magriplis E, Zampelas A, Mitsopoulou AV, Karageorgou D, Dimakopoulos I, et al. The recalibrated hellenicscore based on newly derived risk factors from the hellenic national nutrition and health survey (HNNHS); the hellenicscore II. Hellenic Journal of Cardiology 2020. | A |
| 1. Parikh NI, Jeppson RP, Berger JS, Eaton CB, Kroenke CH, Leblanc ES, et al. Reproductive risk factors and coronary heart disease in the women's health initiative observational study. Circulation 2016;133(22):2149-58. | A |
| 1. Park GM, Han S, Kim SH, Jo MW, Her SH, Lee JB, et al. Model for assessing cardiovascular risk in a korean population. Circulation: Cardiovascular Quality and Outcomes 2014;7(6):944-51. | C |
| 1. Park HE, Chun EJ, Choi SI, Lee SP, Yoon CH, Kim HK, et al. Clinical and imaging parameters to predict cardiovascular outcome in asymptomatic subjects. International Journal of Cardiovascular Imaging 2013;29(7):1595-602. | B |
| 1. Park KH, Kim MK, Kim HS, Park WJ, Cho GY, Choi YJ. Clinical significance of framingham risk score, flow-mediated dilation and pulse wave velocity in patients with stable angina. Circulation Journal 2011;75(5):1177-83. | C |
| 1. Partanapat C, Jaruskulchai C, Jandaeng C. A hybrid model for coronary heart disease prediction in thai population. Advances in Science, Technology and Engineering Systems 2020;5(5):414-25. | A |
| 1. Pasqualini L, Schillaci G, Pirro M, Vaudo G, Siepi D, Innocente S, et al. Renal dysfunction predicts long-term mortality in patients with lower extremity arterial disease. Journal of Internal Medicine 2007;262(6):668-77. | C |
| 1. Patil SS, Joshi R, Gupta G, Reddy MVR, Pai M, Kalantri SP. Risk factors for acute myocardial infarction in a rural population of central india: A hospital-based case-control study. National Medical Journal of India 2004;17(4):189-94. | A |
| 1. Pattarabanjird T, Cress C, Nguyen A, Taylor A, Bekiranov S, McNamara C. A machine learning model utilizing a novel SNP shows enhanced prediction of coronary artery disease severity. Genes 2020;11(12):1-14. | A |
| 1. Paynter NP, Sharrett AR, Louis TA, Rosamond W, Folsom AR, Coresh J. Paired comparison of observed and expected coronary heart disease rates over 12 years from the atherosclerosis risk in communities study. Annals of Epidemiology 2010;20(9):683-90. | A |
| 1. Pencina MJ, D'Agostino RB, Song L. Quantifying discrimination of framingham risk functions with different survival C statistics. Statistics in Medicine 2012;31(15):1543-53. | A |
| 1. Perazzo H, Munteanu M, Ngo Y, Lebray P, Seurat N, Rutka F, et al. Prognostic value of liver fibrosis and steatosis biomarkers in type-2 diabetes and dyslipidaemia. Alimentary Pharmacology and Therapeutics 2014;40(9):1081-93. | C |
| 1. Petersson U, Östgren CJ, Brudin L, Nilsson PM. A consultation-based method is equal to SCORE and an extensive laboratory-based method in predicting risk of future cardiovascular disease. European Journal of Cardiovascular Prevention and Rehabilitation 2009;16(5):536-40. | A |
| 1. Petursson H, Sigurdsson JA, Bengtsson C, Nilsen TIL, Getz L. Is the use of cholesterol in mortality risk algorithms in clinical guidelines valid? Ten years prospective data from the norwegian HUNT 2 study. Journal of Evaluation in Clinical Practice 2012;18(1):159-68. | A |
| 1. Pezel T, Garot P, Kinnel M, Unterseeh T, Hovasse T, Champagne S, et al. Prognostic value of stress cardiovascular magnetic resonance in asymptomatic patients without known coronary artery disease. European Radiology 2021;31(8):6172-83. | A |
| 1. Pfister R, Luben RN, Khaw KT, Wareham NJ. Common genetic variants of the natriuretic peptide gene locus are not associated with heart failure risk in participants in the epic-norfolk study. European Journal of Heart Failure 2013;15(6):624-7. | A |
| 1. Pike MM, Decker PA, Larson NB, St. Sauver JL, Takahashi PY, Roger VL, et al. Improvement in cardiovascular risk prediction with electronic health records. Journal of Cardiovascular Translational Research 2016;9(3):214-22. | D |
| 1. Piko P, Kosa Z, Sandor J, Adany R. Comparative risk assessment for the development of cardiovascular diseases in the hungarian general and roma population. Scientific Reports 2021;11(1). | B |
| 1. Pletcher MJ, Hulley BJ, Houston T, Kiefe CI, Benowitz N, Sidney S. Menthol cigarettes, smoking cessation, atherosclerosis, and pulmonary function: The coronary artery risk development in young adults (CARDIA) study. Archives of Internal Medicine 2006;166(17):1915-22. | A |
| 1. Pletcher MJ, Sibley CT, Pignone M, Vittinghoff E, Greenland P. Interpretation of the coronary artery calcium score in combination with conventional cardiovascular risk factors: The multi- ethnic study of atherosclerosis (MESA). Circulation 2013;128(10):1076-84. | B |
| 1. Polak JF, Ouyang P, Vaidya D. Total brachial artery reactivity and first time incident coronary heart disease events in a longitudinal cohort study: The multi-ethnic study of atherosclerosis. PLoS ONE 2019;14(4). | A |
| 1. Polak JF, Pencina MJ, Pencina KM, O'Donnell CJ, Wolf PA, D'Agostino Sr RB. Carotid-wall intima-media thickness and cardiovascular events. New England Journal of Medicine 2011;365(3):213-21. | A |
| 1. Polak JF, Szklo M, O'Leary DH. Associations of coronary heart disease with common carotid artery near and far wall intima-media thickness: The multi-ethnic study of atherosclerosis. Journal of the American Society of Echocardiography 2015;28(9):1114-21. | A |
| 1. Prasad GVR, Huang M, Silver SA, Al-Lawati AI, Rapi L, Nash MM, Zaltzman JS. Metabolic syndrome definitions and components in predicting major adverse cardiovascular events after kidney transplantation. Transplant International 2015;28(1):79-88. | A |
| 1. Preis SR, Hwang SJ, Fox CS, Massaro JM, Levy D, Hoffmann U, O'Donnell CJ. Eligibility of individuals with subclinical coronary artery calcium and intermediate coronary heart disease risk for reclassification (from the framingham heart study). American Journal of Cardiology 2009;103(12):1710-5. | A |
| 1. Preiss D, Thomas LE, Sun JL, Haffner SM, Holman RR, Standl E, et al. Predictors of cardiovascular events in a contemporary population with impaired glucose tolerance: An observational analysis of the nateglinide and valsartan in impaired glucose tolerance outcomes research (NAVIGATOR) trial. BMJ Open 2012;2(6). | A |
| 1. Price JF, Tzoulaki I, Lee AJ, Fowkes FGR. Ankle brachial index and intima media thickness predict cardiovascular events similarly and increased prediction when combined. Journal of Clinical Epidemiology 2007;60(10):1067-75. | A |
| 1. Psaty BM, Anderson M, Kronmal RA, Tracy RP, Orchard T, Fried LP, et al. The association between lipid levels and the risks of incident myocardial infarction, stroke, and total mortality: The cardiovascular health study. Journal of the American Geriatrics Society 2004;52(10):1639-47. | A |
| 1. Quercioli A, Montecucco F, Galan K, Ratib O, Roux-Lombard P, Pagano S, et al. Anti- apolipoprotein A-1 igg levels predict coronary artery calcification in obese but otherwise healthy individuals. Mediators of Inflammation 2012;2012. | A |
| 1. Quirke TP, Gill PS, Mant JW, Allan TF. The applicability of the framingham coronary heart disease prediction function to black and minority ethnic groups in the UK. Heart 2003;89(7):785-6. | C |
| 1. Raggi P, Cooil B, Callister TQ. Use of electron beam tomography data to develop models for prediction of hard coronary events. American Heart Journal 2001;141(3):375-82. | A |
| 1. Raggi P, Cooil B, Ratti C, Callister TQ, Budoff M. Progression of coronary artery calcium and occurrence of myocardial infarction in patients with and without diabetes mellitus. Hypertension 2005;46(1):238-43. | A |
| 1. Raggi P, Shaw LJ, Berman DS, Callister TQ. Prognostic value of coronary artery calcium screening in subjects with and without diabetes. Journal of the American College of Cardiology 2004;43(9):1663-9. | B |
| 1. Raihan M, Mondal S, More A, Boni PK, Sagor MOF. Smartphone based heart attack risk prediction system with statistical analysis and data mining approaches. Advances in Science, Technology and Engineering Systems 2017;2(3):1815-22. | A |
| 1. Ramírez-Rodrigo J, Moreno-Vázquez JA, Ruiz-Villaverde A, Sánchez-Caravaca M, Lopez De La Torre-Casares M, Villaverde-Gutiérrez C. A computer tool for cardiovascular risk estimation according to framingham and SCORE equations. Journal of Evaluation in Clinical Practice 2013;19(2):277-84. | A |
| 1. Ramos R, Solanas P, Cordón F, Rohlfs I, Elosua R, Sala J, et al. Comparison of population coronary heart disease risk estimated by the framingham original and REGICOR calibrated functions. Medicina Clinica 2003;121(14):521-6. | A |
| 1. Ray WA, Chung CP, Murray KT, Hall K, Stein CM. Atypical antipsychotic drugs and the risk of sudden cardiac death. New England Journal of Medicine 2009;360(3):225-35. | A |
| 1. Reissigová J, Zvárová J. Estimates of absolute cardiovascular risk in the czech republic. Prakticky Lekar 2008;88(5):288-92. | A |
| 1. Ren Y, Wei B, Song Y, Guo H, Zhang X, Wang X, et al. Factor analysis of metabolic syndrome and its relationship with the risk of cardiovascular disease in ethnic populations in rural xinjiang, china. International Journal of General Medicine 2021;14:4317-25. | A |
| 1. Ridker PM, Buring JE, Rifai N, Cook NR. Development and validation of improved algorithms for the assessment of global cardiovascular risk in women: The reynolds risk score. JAMA - Journal of the American Medical Association 2007;297(6):611-9. | A |
| 1. Ridker PM, Rifai N, Rose L, Buring JE, Cook NR. Comparison of c-reactive protein and low- density lipoprotein cholesterol levels in the prediction of first cardiovascular events. New England Journal of Medicine 2002;347(20):1557-65. | A |
| 1. Riveros BS, Torelli Reis WC, Lucchetta RC, Moreira LB, Lewsey J, Correr CJ, Wu O. Brazilian analytical decision model for cardiovascular disease: An adaptation of the scottish cardiovascular disease policy model. Value in Health Regional Issues 2018;17:210-6. | A |
| 1. Ryoo JH, Cho SH, Kim SW. Prediction of risk factors for coronary heart disease using framingham risk score in korean men. PLoS ONE 2012;7(9). | B |
| 1. Sacco RL, Khatri M, Rundek T, Xu Q, Gardener H, Boden-Albala B, et al. Improving global vascular risk prediction with behavioral and anthropometric factors. The multiethnic NOMAS (northern manhattan cohort study). Journal of the American College of Cardiology 2009;54(24):2303-11. | A |
| 1. Sajid MR, Muhammad N, Zakaria R, Shahbaz A, Bukhari SAC, Kadry S, Suresh A. Nonclinical features in predictive modeling of cardiovascular diseases: A machine learning approach. Interdisciplinary Sciences – Computational Life Sciences 2021;13(2):201-11. | A |
| 1. Salameh MJ, Rundek T, Boden-Albala B, Jin Z, Ratchford EV, Di Tullio MR, et al. Self-reported peripheral arterial disease predicts future vascular events in a community-based cohort. Journal of General Internal Medicine 2008;23(9):1423-8. | A |
| 1. Salminen M, Kuoppamäki M, Vahlberg T, Räihä I, Irjala K, Kivelä SL. Metabolic syndrome and vascular risk: A 9-year follow-up among the aged in finland. Acta Diabetologica 2011;48(2):157-65. | A |
| 1. Salminen M, Kuoppamäki M, Vahlberg T, Räihä I, Irjala K, Kivelä SL. The metabolic syndrome defined by modified international diabetes federation criteria and mortality: A 9-year follow-up of the aged in finland. Diabetes and Metabolism 2010;36(6):437-42. | A |
| 1. Samad MD, Ulloa A, Wehner GJ, Jing L, Hartzel D, Good CW, et al. Predicting survival from large echocardiography and electronic health record datasets: Optimization with machine learning. JACC: Cardiovascular Imaging 2019;12(4):681-9. | C |
| 1. Sánchez-Íñigo L, Navarro-González D, Fernández-Montero A, Pastrana-Delgado J, Martínez JA. The tyg index may predict the development of cardiovascular events. European Journal of Clinical Investigation 2016;46(2):189-97. | A |
| 1. Santora LJ, Pillutla P, Norris T, Santora R, Brandt R, Jenkins M, et al. Coronary calcium scanning independently detects coronary artery disease in asymptomatic firefighters: A prospective study. Journal of Cardiovascular Computed Tomography 2013;7(1):46-50. | A |
| 1. Santos AC, Barros H. Impact of metabolic syndrome definitions on prevalence estimates: A study in a portuguese community. Diabetes and Vascular Disease Research 2007;4(4):320-7. | A |
| 1. Sbrana F, Puntoni M, Bigazzi F, Landi P, Sampietro T, Rossi G, Rovai D. High density lipoprotein cholesterol in coronary artery disease: When higher means later. Journal of Atherosclerosis and Thrombosis 2013;20(1):23-31. | A |
| 1. Schisterman EF, Whitcomb BW. Coronary age as a risk factor in the modified framingham risk score. BMC Medical Imaging 2004;4. | B |
| 1. Schneeweiss S, Seeger JD, Maclure M, Wang PS, Avorn J, Glynn RJ. Performance of comorbidity scores to control for confounding in epidemiologic studies using claims data. American Journal of Epidemiology 2001;154(9):854-64. | A |
| 1. Schneider HJ, Wallaschofski H, Völzke H, Markus MRP, Doerr M, Felix SB, et al. Incremental effects of endocrine and metabolic biomarkers and abdominal obesity on cardiovascular mortality prediction. PLoS ONE 2012;7(3). | A |
| 1. Schoening W, Buescher N, Neidel N, Helbig M, Andreou A, Pascher A, et al. Cerebrovascular events in 20 years of follow-up after liver transplantation: An underestimated issue? Clinical Transplantation 2016;30(10):1276-82. | A |
| 1. Schouten BWV, Bohnen AM, Bosch JLHR, Bernsen RMD, Deckers JW, Dohle GR, Thomas S. Erectile dysfunction prospectively associated with cardiovascular disease in the dutch general population: Results from the krimpen study. International Journal of Impotence Research 2008;20(1):92-9. | B |
| 1. Schulz CA, Mavarani L, Reinsch N, Albayrak-Rena S, Potthoff A, Brockmeyer N, et al. Prediction of future cardiovascular events by framingham, SCORE and ascvd risk scores is less accurate in hiv-positive individuals from the HIV-HEART study compared with the general population. HIV Medicine 2021;22(8):732-41. | C |
| 1. Schulze MB, Hoffmann K, Boeing H, Linseisen J, Rohrmann S, Möhlig M, et al. An accurate risk score based on anthropometric, dietary, and lifestyle factors to predict the development of type 2 diabetes. Diabetes Care 2007;30(3):510-5. | A |
| 1. Sedlak T, Herscovici R, Cook-Wiens G, Handberg E, Wei J, Shufelt C, et al. Predicted versus observed major adverse cardiac event risk in women with evidence of ischemia and no obstructive coronary artery disease: A report from wise (women’s ischemia syndrome evaluation). Journal of the American Heart Association 2020;9(7). | B |
| 1. Sehestedt T, Hansen TW, Li Y, Richart T, Boggia J, Kikuya M, et al. Are blood pressure and diabetes additive or synergistic risk factors outcome in 8494 subjects randomly recruited from 10 populations. Hypertension Research 2011;34(6):714-21. | A |
| 1. Setayeshgar S, Whiting SJ, Vatanparast H. Prevalence of 10-year risk of cardiovascular diseases and associated risks in canadian adults: The contribution of cardiometabolic risk assessment introduction. International Journal of Hypertension 2013;2013. | A |
| 1. Shah AJ, Veledar E, Hong Y, Bremner JD, Vaccarino V. Depression and history of attempted suicide as risk factors for heart disease mortality in young individuals. Archives of General Psychiatry 2011;68(11):1135-42. | C |
| 1. Shah T, Casas JP, Cooper JA, Tzoulaki I, Sofat R, McCormack V, et al. Critical appraisal of CRP measurement for the prediction of coronary heart disease events: New data and systematic review of 31 prospective cohorts. International Journal of Epidemiology 2009;38(1):217-31. | A |
| 1. Shang E, Sakauchi F, Washio M, Sonoda T, Ikeda T, Mori M. Predictive index of coronary heart disease (CHD) risk based on the results of health check up. Sapporo Medical Journal 2004;73(1-3):13-7. | A |
| 1. Shanker J, Kakkar VV. Contribution of classical and emerging risk factors to coronary artery disease in asian indians. International Journal of Cardiology 2016;214:97-106. | A |
| 1. Sharma TS, Wasko MM, Tang X, Vedamurthy D, Yan X, Cote J, Bili A. Hydroxychloroquine use is associated with decreased incident cardiovascular events in rheumatoid arthritis patients. Journal of the American Heart Association 2016;5(1). | A |
| 1. Shaw LJ, Raggi P, Callister TQ, Berman DS. Prognostic value of coronary artery calcium screening in asymptomatic smokers and non-smokers. European Heart Journal 2006;27(8):968-75. | A |
| 1. Shaw LJ, Raggi P, Schisterman E, Berman DS, Callister TQ. Prognostic value of cardiac risk factors and coronary artery calcium screening for all-cause mortality. Radiology 2003;228(3):826-33. | B |
| 1. Sheridan SL, Draeger LB, Pignone MP, Keyserling TC, Simpson RJ, Rimer B, et al. A randomized trial of an intervention to improve use and adherence to effective coronary heart disease prevention strategies. BMC Health Services Research 2011;11. | A |
| 1. Shiffman D, O'Meara ES, Rowland CM, Louie JZ, Cushman M, Tracy RP, et al. The contribution of a 9p21.3 variant, a KIF6 variant, and c-reactive protein to predicting risk of myocardial infarction in a prospective study. BMC Cardiovascular Disorders 2011;11. | A |
| 1. Silventoinen K, Pankow J, Lindström J, Jousilahti P, Hu G, Tuomilehto J. The validity of the finnish diabetes risk score for the prediction of the incidence of coronary heart disease and stroke, and total mortality. European Journal of Cardiovascular Prevention and Rehabilitation 2005;12(5):451-8. | A |
| 1. Silver SA, Huang M, Nash MM, Prasad GVR. Framingham risk score and novel cardiovascular risk factors underpredict major adverse cardiac events in kidney transplant recipients. Transplantation 2011;92(2):183-9. | D |
| 1. Silverman MG, Blaha MJ, Budoff MJ, Rivera JJ, Raggi P, Shaw LJ, et al. Potential implications of coronary artery calcium testing for guiding aspirin use among asymptomatic individuals with diabetes. Diabetes Care 2012;35(3):624-6. | A |
| 1. Simmonds MC, Jwald N. Risk estimation versus screening performance: A comparison of six risk algorithms for cardiovascular disease. Journal of Medical Screening 2012;19(4):201-5. | B |
| 1. Simons LA, Simons J, Friedlander Y, McCallum J, Palaniappan L. Risk functions for prediction of cardiovascular disease in elderly australians: The dubbo study. Medical Journal of Australia 2003;178(3):113-6. | B |
| 1. Sivakumaran J, Harvey P, Omar A, Tayer-Shifman O, Urowitz MB, Gladman DD, et al. Assessment of cardiovascular risk tools as predictors of cardiovascular disease events in systemic lupus erythematosus. Lupus Science and Medicine 2021;8(1). | B |
| 1. Skaarup KG, Lassen MCH, Marott JL, Biering-Sørensen SR, Johansen ND, Modin D, et al. Diastolic function assessed with speckle tracking over a decade and its prognostic value: The copenhagen city heart study. Echocardiography 2021;38(6):964-73. | A |
| 1. Skogsberg J, McMahon AD, Karpe F, Hamsten A, Packard CJ, Ehrenborg E. Peroxisome proliferator activated receptor delta genotype in relation to cardiovascular risk factors and risk of coronary heart disease in hypercholesterolaemic men. Journal of Internal Medicine 2003;254(6):597-604. | A |
| 1. Smink PA, Lambers Heerspink HJ, Gansevoort RT, De Jong PE, Hillege HL, Bakker SJL, De Zeeuw D. Albuminuria, estimated GFR, traditional risk factors, and incident cardiovascular disease: The PREVEND (prevention of renal and vascular endstage disease) study. American Journal of Kidney Diseases 2012;60(5):804-11. | A |
| 1. Sobchak C, Akhtari S, Harvey P, Gladman D, Chandran V, Cook R, Eder L. Value of carotid ultrasound in cardiovascular risk stratification in patients with psoriatic disease. Arthritis and Rheumatology 2019;71(10):1651-9. | B |
| 1. Solomon DH, Greenberg J, Curtis JR, Liu M, Farkouh ME, Tsao P, et al. Derivation and internal validation of an expanded cardiovascular risk prediction score for rheumatoid arthritis: A consortium of rheumatology researchers of north america registry study. Arthritis and Rheumatology 2015;67(8):1995-2003. | A |
| 1. Sosnowski M, Kozakiewicz K, Syzdól M, Nadrowski P, Mlynarski R, Brzoska J, Tendera M. Coronary artery calcium in type 2 diabetes: A nested case-control study. Polskie Archiwum Medycyny Wewnetrznej 2013;123(1-2):38-44. | A |
| 1. Stamler J, Stamler R, Neaton JD, Wentworth D, Daviglus ML, Garside D, et al. Low risk-factor profile and long-term cardiovascular and noncardiovascular mortality and life expectancy. Findings for 5 large cohorts of young adult and middle-aged men and women. JAMA - Journal of the American Medical Association 1999;282(21):2012-8. | A |
| 1. Staniak HL, Bittencourt MS, de Souza Santos I, Sharovsky R, Sabbag C, Goulart AC, et al. Association between psoriasis and coronary calcium score. Atherosclerosis 2014;237(2):847-52. | B |
| 1. Stengård JH, Dyson G, Frikke-Schmidt R, Tybjærg-Hansen A, Nordestgaard BG, Sing CF. Context-dependent associations between variation in risk of ischemic heart disease and variation in the 5ε promoter region of the apolipoprotein E gene in danish women. Circulation: Cardiovascular Genetics 2010;3(1):22-30. | A |
| 1. Stern MP, Williams K, González-Villalpando C, Hunt KJ, Haffner SM. Does the metabolic- syndrome improve identification of individuals at risk of type 2 diabetes and/or cardiovascular disease? Diabetes Care 2004;27(11):2676-81. | C |
| 1. Stinson LJ, Stroud LR, Buka SL, Eaton CB, Lu B, Niaura R, Loucks EB. Prospective evaluation of associations between prenatal cortisol and adulthood coronary heart disease risk: The new england family study. Psychosomatic Medicine 2015;77(3):237-45. | C |
| 1. Storti FC, Moffa PJ, Uchida AH, Hueb WA, César LAM, Ferreira BMA, et al. New prognostic score for stable coronary disease evaluation. Arquivos Brasileiros De Cardiologia 2011;96(5):411-8. | A |
| 1. Sullivan LM, Massaro JM, D'Agostino Sr RB. Presentation of multivariate data for clinical use: The framingham study risk score functions. Statistics in Medicine 2004;23(10):1631-60. | A |
| 1. Sung J, Lim SJ, Choe Y, Choi YH, Lee MK, Lee SH, et al. Comparison of the coronary calcium score with the estimated coronary risk. Coronary Artery Disease 2008;19(7):475-9. | A |
| 1. Tabaei BP, Chamany S, Perlman S, Thorpe L, Bartley K, Wu WY. Heart age, cardiovascular disease risk, and disparities by sex and race/ethnicity among new york city adults. Public Health Reports 2019;134(4):404-16. | B |
| 1. Takeuchi T, Nemoto KI, Takahashi O, Urayama KY, Deshpande GA, Izumo H. Comparison of cardiovascular disease risk associated with 3 lipid measures in japanese adults. Journal of Clinical Lipidology 2014;8(5):501-9. | A |
| 1. Talmud PJ, Hingorani AD, Cooper JA, Marmot MG, Brunner EJ, Kumari M, et al. Utility of genetic and non-genetic risk factors in prediction of type 2 diabetes: Whitehall II prospective cohort study. BMJ (Online) 2010;340(7739):192. | B |
| 1. Tanabe N, Iso H, Okada K, Nakamura Y, Harada A, Ohashi Y, et al. Serum total and non-high- density lipoprotein cholesterol and the risk prediction of cardiovascular events: - the jals-ecc. Circulation Journal 2010;74(7):1347-56. | A |
| 1. Tanaka F, Makita S, Onoda T, Tanno K, Ohsawa M, Itai K, et al. Predictive value of lipoprotein indices for residual risk of acute myocardial infarction and sudden death in men with low-density lipoprotein cholesterol levels <120 mg/dl. American Journal of Cardiology 2013;112(8):1063-8. | A |
| 1. Teramoto T, Ohashi Y, Nakaya N, Yokoyama S, Mizuno K, Nakamura H. Practical risk prediction tools for coronary heart disease in mild to moderate hypercholesterolemia in japan - originated from the MEGA study data. Circulation Journal 2008;72(10):1569-75. | A |
| 1. Thomas IC, McClelland RL, Michos ED, Allison MA, Forbang NI, Longstreth WTJ, et al. Density of calcium in the ascending thoracic aorta and risk of incident cardiovascular disease events. Atherosclerosis 2017;265:190-6. | A |
| 1. Thompson-Paul AM, Lichtenstein KA, Armon C, Palella FJJ, Skarbinski J, Chmiel JS, et al. Cardiovascular disease risk prediction in the HIV outpatient study. Clinical Infectious Diseases 2016;63(11):1508-16. | B |
| 1. Thomsen TF, McGee D, Davidsen M, Jørgensen T. A cross-validation of risk-scores for coronary heart disease mortality based on data from the glostrup population studies and framingham heart study. International Journal of Epidemiology 2002;31(4):817-22. | A |
| 1. Thurston RC, Kubzansky LD. Multiple sources of psychosocial disadvantage and risk of coronary heart disease. Psychosomatic Medicine 2007;69(8):748-55. | A |
| 1. Tindle HA, Chang YF, Kuller LH, Manson JE, Robinson JG, Rosal MC, et al. Optimism, cynical hostility, and incident coronary heart disease and mortality in the women's health initiative. Circulation 2009;120(8):656-62. | A |
| 1. Tohidi M, Hatami M, Hadaegh F, Safarkhani M, Harati H, Azizi F. Lipid measures for prediction of incident cardiovascular disease in diabetic and non-diabetic adults: Results of the 8.6 years follow-up of a population based cohort study. Lipids in Health and Disease 2010;9. | A |
| 1. Tonelli M, Muntner P, Lloyd A, Manns BJ, Klarenbach S, Pannu N, et al. Risk of coronary events in people with chronic kidney disease compared with those with diabetes: A population-level cohort study. The Lancet 2012;380(9844):807-14. | A |
| 1. Topel ML, Shen J, Morris AA, Al Mheid I, Sher S, Dunbar SB, et al. Comparisons of the framingham and pooled cohort equation risk scores for detecting subclinical vascular disease in blacks versus whites. American Journal of Cardiology 2018;121(5):564-9. | C |
| 1. Torres T, Sales R, Vasconcelos C, Martins Da Silva B, Selores M. Framingham risk score underestimates cardiovascular disease risk in severe psoriatic patients: Implications in cardiovascular risk factors management and primary prevention of cardiovascular disease. Journal of Dermatology 2013;40(11):923-6. | A |
| 1. Towfighi A, Markovic D, Ovbiagele B. Temporal trends in risk of future cardiac events among stroke survivors in the united states. International Journal of Stroke 2012;7(3):207-12. | A |
| 1. Towfighi A, Markovic D, Ovbiagele B. Utility of framingham coronary heart disease risk score for predicting cardiac risk after stroke. Stroke 2012;43(11):2942-7. | C |
| 1. Treeprasertsuk S, Björnsson E, Enders F, Suwanwalaikorn S, Lindor KD. NAFLD fibrosis score: A prognostic predictor for mortality and liver complications among NAFLD patients. World Journal of Gastroenterology 2013;19(8):1219-29. | A |
| 1. Triant VA, Perez J, Regan S, Massaro JM, Meigs JB, Grinspoon SK, D'Agostino RBS. Cardiovascular risk prediction functions underestimate risk in HIV infection. Circulation 2018;137(21):2203-14. | A |
| 1. Troughton JA, Woodside JV, Yarnell JWG, Arveiler D, Amouyel P, Ferrières J, et al. Paraoxonase activity and coronary heart disease risk in healthy middle-aged males: The PRIME study. Atherosclerosis 2008;197(2):556-63. | A |
| 1. Tsai CC, Hsieh MH, Li AH, Chen PL, Jeng C. Dietary supplementation and engaging in physical activity as predictors of coronary artery disease among middle-aged women. Journal of Clinical Nursing 2013;22(17-18):2487-98. | A |
| 1. Tsang TSM, Barnes ME, Gersh BJ, Takemoto Y, Rosales AG, Bailey KR, Seward JB. Prediction of risk for first age-related cardiovascular events in an elderly population: The incremental value of echocardiography. Journal of the American College of Cardiology 2003;42(7):1199-205. | A |
| 1. Tuomilehto J, Lindström J, Hellmich M, Lehmacher W, Westermeier T, Evers T, et al. Development and validation of a risk-score model for subjects with impaired glucose tolerance for the assessment of the risk of type 2 diabetes mellitus-the STOP-NIDDM risk-score. Diabetes Research and Clinical Practice 2010;87(2):267-74. | A |
| 1. Tzoulaki I, Liberopoulos G, Ioannidis JPA. Assessment of claims of improved prediction beyond the framingham risk score. JAMA - Journal of the American Medical Association 2009;302(21):2345-52. | A |
| 1. Ukah UV, Dayan N, Auger N, He S, Platt RW. Development and internal validation of a model predicting premature cardiovascular disease among women with hypertensive disorders of pregnancy: A population-based study in quebec, canada. Journal of the American Heart Association 2020;9(20). | A |
| 1. Uthoff H, Staub D, Socrates T, Meyerhans A, Bundi B, Schmid HP, Frauchiger B. Procam-, framingham-, score- and smart-risk score for predicting cardiovascular morbidity and mortality in patients with overt atherosclerosis. Vasa - Journal of Vascular Diseases 2010;39(4):325-33. | C |
| 1. Vaarhorst AAM, Verhoeven A, Weller CM, Böhringer S, Göraler S, Meissner A, et al. A metabolomic profile is associated with the risk of incident coronary heart disease. American Heart Journal 2014;168(1):45-52.e7. | A |
| 1. van der Net JB, Janssens ACJW, Defesche JC, Kastelein JJP, Sijbrands EJG, Steyerberg EW. Usefulness of genetic polymorphisms and conventional risk factors to predict coronary heart disease in patients with familial hypercholesterolemia. American Journal of Cardiology 2009;103(3):375-80. | A |
| 1. Van Dis I, Geleijnse JM, Boer JMA, Kromhout D, Boshuizen H, Grobbee DE, et al. Effect of including nonfatal events in cardiovascular risk estimation, illustrated with data from the netherlands. European Journal of Preventive Cardiology 2014;21(3):377-83. | A |
| 1. Van Kempen BJH, S. Ferket B, Kavousi M, J.g. Leening M, W. Steyerberg E, Ikram MA, et al. Performance of framingham cardiovascular disease (CVD) predictions in the rotterdam study taking into account competing risks and disentangling CVD into coronary heart disease (CHD) and stroke. International Journal of Cardiology 2014;171(3):413-8. | B |
| 1. Vartiainen E, Laatikainen T, Peltonen M, Puska P. Predicting coronary heart disease and stroke: The FINRISK calculator. Global Heart 2016;11(2):213-6. | A |
| 1. Vasan RS, Sullivan LM, Wilson PWF, Sempos CT, Sundström J, Kannel WB, et al. Relative importance of borderline and elevated levels of coronary heart disease risk factors. Annals of Internal Medicine 2005;142(6):393-402+I-23. | A |
| 1. Vasile VC, Meeusen JW, Medina Inojosa JR, Donato LJ, Scott CG, Hyun MS, et al. Ceramide scores predict cardiovascular risk in the community. Arteriosclerosis, Thrombosis, and Vascular Biology 2021;41(4):1558-69. | A |
| 1. Veeranna V, Zalawadiya SK, Niraj A, Pradhan J, Ference B, Burack RC, et al. Homocysteine and reclassification of cardiovascular disease risk. Journal of the American College of Cardiology 2011;58(10):1025-33. | B |
| 1. Velescu A, Clara A, Peñafiel J, Ramos R, Marti R, Grau M, et al. Adding low ankle brachial index to classical risk factors improves the prediction of major cardiovascular events: The REGICOR study. Atherosclerosis 2015;241(2):357-63. | B |
| 1. Venkatesh S, O'Neal WT, Broughton ST, Shah AJ, Soliman EZ. Utility of normal findings on electrocardiogram and echocardiogram in subjects ≥65 years. American Journal of Cardiology 2017;119(6):856-61. | A |
| 1. Verdiesen RMG, Onland-Moret NC, van Gils CH, van der Schouw YT. Circulating anti-müllerian hormone levels and markers of subclinical cardiovascular disease in middle-aged and older men. Maturitas 2022;163:38-45. | C |
| 1. Vergallo R, Xing L, Minami Y, Soeda T, Ong DS, Gao L, et al. Associations between the framingham risk score and coronary plaque characteristics as assessed by three-vessel optical coherence tomography. Coronary Artery Disease 2016;27(6):460-6. | D |
| 1. Vergnaud AC, Bertrais S, Galan P, Hercberg S, Czernichow S. Ten-year risk prediction in french men using the framingham coronary score: Results from the national SU.VI.MAX cohort. Preventive Medicine 2008;47(1):61-5. | B |
| 1. Veronesi G, Gianfagna F, Chambless LE, Giampaoli S, Mancia G, Cesana G, Ferrario MM. Long- term prediction of major coronary or ischaemic stroke event in a low-incidence southern european population: Model development and evaluation of clinical utility. BMJ Open 2013;3(11). | A |
| 1. Viera AJ, Sheridan SL. Global risk of coronary heart disease: Assessment and application. American Family Physician 2010;82(3):265-74. | A |
| 1. Vittecoq D, Escaut L, Chironi G, Teicher E, Monsuez JJ, Andrejak M, Simon A. Coronary heart disease in hiv-infected patients in the highly active antiretroviral treatment era. AIDS 2003;17(SUPPL. 1):S70-6. | A |
| 1. Vliegenthart R, Oudkerk M, Song B, Van Der Kuip DAM, Hofman A, Witteman JCM. Coronary calcification detected by electron-beam computed tomography and myocardial infarction: The rotterdam coronary calcification study. European Heart Journal 2002;23(20):1596-603. | A |
| 1. Vlismas K, Panagiotakos DB, Pitsavos C, Chrysohoou C, Skoumas Y, Stavrinos V, Stefanadis C. The role of dietary and socioeconomic status assessment on the predictive ability of the hellenicscore. Hellenic Journal of Cardiology 2011;52(5):391-8. | A |
| 1. Wadhwa D, Mahajan VK, Mehta KS, Chauhan PS, Yadav RS, Bhushan S, et al. Malondialdehyde, lipoprotein-a, lipoprotein ratios, comprehensive lipid tetrad index and atherogenic index as surrogate markers for cardiovascular disease in patients with psoriasis: A case–control study. Archives of Dermatological Research 2019;311(4):287-97. | A |
| 1. Wallis EJ, Ramsay LE, Haq IU, Ghahramani P, Jackson PR, Rowland-Yeo K, Yeo WW. Coronary and cardiovascular risk estimation for primary prevention: Validation of a new sheffield table in the 1995 scottish health survey population. British Medical Journal 2000;320(7236):671-675,676. | A |
| 1. Wan Z, Liu X, Wang X, Liu F, Liu W, Wu Y, et al. Small artery elasticity predicts future cardiovascular events in chinese patients with angiographic coronary artery disease. Angiology 2014;65(4):298-302. | A |
| 1. Wang C, Zhao Y, Jin B, Gan X, Liang B, Xiang Y, et al. Development and validation of a predictive model for coronary artery disease using machine learning. Frontiers in Cardiovascular Medicine 2021;8. | A |
| 1. Wang CH, Chen CJ, Lee MH, Yang HI, Hsiao CK. Chronic hepatitis B infection and risk of atherosclerosis-related mortality: A 17-year follow-up study based on 22,472 residents in taiwan. Atherosclerosis 2010;211(2):624-9. | A |
| 1. Wang TJ, Gona P, Larson MG, Tofler GH, Levy D, Newton-Cheh C, et al. Multiple biomarkers for the prediction of first major cardiovascular events and death. New England Journal of Medicine 2006;355(25):2631-9. | A |
| 1. Wannamethee SG, Welsh P, Lowe GD, Gudnason V, Di Angelantonio E, Lennon L, et al. N- terminal pro-brain natriuretic peptide is a more useful predictor of cardiovascular disease risk than c-reactive protein in older men with and without pre-existing cardiovascular disease. Journal of the American College of Cardiology 2011;58(1):56-64. | A |
| 1. Weale ME, Riveros-Mckay F, Selzam S, Seth P, Moore R, Tarran WA, et al. Validation of an integrated risk tool, including polygenic risk score, for atherosclerotic cardiovascular disease in multiple ethnicities and ancestries. American Journal of Cardiology 2021;148:157-64. | A |
| 1. Wehrberger C, Temml C, Gutjahr G, Berger I, Rauchenwald M, Ponholzer A, Madersbacher S. Is there an association between lower urinary tract symptoms and cardiovascular risk in men? A cross sectional and longitudinal analysis. Urology 2011;78(5):1063-7. | C |
| 1. Wei T, Yang B, Liu H, Xin F, Fu L. Development and validation of a nomogram to predict coronary heart disease in patients with rheumatoid arthritis in northern china. Aging 2020;12(4):3190-204. | C |
| 1. Wei T, Yang B, Liu H, Xin F, Fu L. Development and validation of a nomogram to predict coronary heart disease in patients with rheumatoid arthritis in northern china. Aging 2020;12(4):3190-204. | D |
| 1. Weinberg I, Gona P, O'Donnell CJ, Jaff MR, Murabito JM. The systolic blood pressure difference between arms and cardiovascular disease in the framingham heart study. American Journal of Medicine 2014;127(3):209-15. | C |
| 1. Weiner DE, Tighiouart H, Elsayed EF, Griffith JL, Salem DN, Levey AS, Sarnak MJ. The framingham predictive instrument in chronic kidney disease. Journal of the American College of Cardiology 2007;50(3):217-24. | A |
| 1. Weiner DE, Tighiouart H, Griffith JL, Elsayed E, Levey AS, Salem DN, Sarnak MJ. Kidney disease, framingham risk scores, and cardiac and mortality outcomes. American Journal of Medicine 2007;120(6):552.e1-8. | A |
| 1. Wells BJ, Roth R, Nowacki AS, Arrigain S, Yu C, Rosenkrans Jr WA, Kattan MW. Prediction of morbidity and mortality in patients with type 2 diabetes. PeerJ 2013;2013(1). | D |
| 1. Wilson PWF, Bozeman SR, Burton TM, Hoaglin DC, Ben-Joseph R, Pashos CL. Prediction of first events of coronary heart disease and stroke with consideration of adiposity. Circulation 2008;118(2):124-30. | A |
| 1. Wilson PWF, Pencina M, Jacques P, Selhub J, D'Agostino R, O'Donnell CJ. C-reactive protein and reclassification of cardiovascular risk in the framingham heart study. Circulation: Cardiovascular Quality and Outcomes 2008;1(2):92-7. | A |
| 1. Wolbers M, Koller MT, Witteman JCM, Steyerberg EW. Prognostic models with competing risks methods and application to coronary risk prediction. Epidemiology 2009;20(4):555-61. | A |
| 1. Woodward M, Brindle P, Tunsfall-Pedoe H. Adding social deprivation and family history to cardiovascular risk assessment: The ASSIGN score from the scottish heart health extended cohort (SHHEC). Heart 2007;93(2):172-6. | C |
| 1. Woodward M. Coronary risk prediction for those with and without diabetes. European Journal of Cardiovascular Prevention and Rehabilitation 2006;13(1):30-6. | A |
| 1. Wright KA, Crowson CS, Michet CJ, Matteson EL. Time trends in incidence, clinical features, and cardiovascular disease in ankylosing spondylitis over three decades: A population-based study. Arthritis Care and Research 2015;67(6):836-41. | C |
| 1. Wu Y, Liu X, Li X, Li Y, Zhao L, Chen Z, et al. Estimation of 10-year risk of fatal and nonfatal ischemic cardiovascular diseases in chinese adults. Circulation 2006;114(21):2217-25. | A |
| 1. Yaghoubvand E, Aghili R, Khajavi A, Khamseh ME. Cardiovascular events in people with type 2 diabetes: Performance of framingham, ukpds, and advance risk equations. Acta Medica Iranica 2021;59(10):610-6. | D |
| 1. Yagi H, Kawai M, Komukai K, Ogawa T, Minai K, Nagoshi T, et al. Impact of chronic kidney disease on the severity of initially diagnosed coronary artery disease and the patient prognosis in the japanese population. Heart and Vessels 2011;26(4):370-8. | A |
| 1. Yamamoto-Honda R, Ehara H, Kitazato H, Takahashi Y, Kawazu S, Akanuma Y, Noda M. The long- term coronary heart disease risk of previously obese patients with type 2 diabetes mellitus. BMC Endocrine Disorders 2013;13. | A |
| 1. Yanek LR, Kral BG, Moy TF, Vaidya D, Lazo M, Becker LC, Becker DM. Effect of positive well- being on incidence of symptomatic coronary artery disease. American Journal of Cardiology 2013;112(8):1120-5. | A |
| 1. Yang EY, Nambi V, Tang Z, Virani SS, Boerwinkle E, Hoogeveen RC, et al. Clinical implications of JUPITER (justification for the use of statins in prevention: An intervention trial evaluating rosuvastatin) in a U.S. Population. Insights from the ARIC (atherosclerosis risk in communities) study. Journal of the American College of Cardiology 2009;54(25):2388-95. | A |
| 1. Yawn BP, Wollan PC, Yawn RA, Jacobsen SJ, Roger V. The gender specific frequency of risk factor and CHD diagnoses prior to incident MI: A community study. BMC Family Practice 2007;8. | A |
| 1. Yayan J. Weak prediction power of the framingham risk score for coronary artery disease in nonagenarians. PLoS ONE 2014;9(11). | A |
| 1. Ye S, Shaffer JA, Kang MS, Harlapur M, Muntner P, Epel E, et al. Relation between leukocyte telomere length and incident coronary heart disease events (from the 1995 canadian nova scotia health survey). American Journal of Cardiology 2013;111(7):962-7. | A |
| 1. Ye S, Willeit J, Kronenberg F, Xu Q, Kiechl S. Association of genetic variation on chromosome 9p21 with susceptibility and progression of atherosclerosis. A population-based, prospective study. Journal of the American College of Cardiology 2008;52(5):378-84. | A |
| 1. Yeh JS, Cheng HM, Hsu PF, Sung SH, Liu WL, Fang HL, Chuang SY. Hysterectomy in young women associates with higher risk of stroke: A nationwide cohort study. International Journal of Cardiology 2013;168(3):2616-21. | A |
| 1. Yingchoncharoen T, Limpijankit T, Jongjirasiri S, Laothamatas J, Yamwong S, Sritara P. Arterial stiffness contributes to coronary artery disease risk prediction beyond the traditional risk score (RAMA-EGAT score). Heart Asia 2012;4(1):77-82. | A |
| 1. Yoda S, Nakanishi K, Tano A, Hori Y, Hayase M, Mineki T, et al. Prognostic value of major cardiac event risk score estimated with gated myocardial perfusion imaging in japanese patients with coronary artery disease. International Heart Journal 2016;57(4):408-16. | A |
| 1. Yoo CS, Lee K, Yi SH, Kim JS, Kim HC. Association of heart rate variability with the framingham risk score in healthy adults. Korean Journal of Family Medicine 2011;32(6):334-40. | A |
| 1. Yoshida M, Mita T, Yamamoto R, Shimizu T, Ikeda F, Ohmura C, et al. Combination of the framingham risk score and carotid intima-media thickness improves the prediction of cardiovascular events in patients with type 2 diabetes. Diabetes Care 2012;35(1):178-80. | C |
| 1. Young JB, Gauthier-Loiselle M, Bailey RA, Manceur AM, Lefebvre P, Greenberg M, et al. Development of predictive risk models for major adverse cardiovascular events among patients with type 2 diabetes mellitus using health insurance claims data. Cardiovascular Diabetology 2018;17(1). | A |
| 1. Yuan N, Scherzer R, Tanriverdi K, Martin J, Rahalkar S, Hsue P. MicroRNA biomarkers associated with type 1 myocardial infarction in hiv-positive individuals. AIDS 2019;33(15):2351-61. | B |
| 1. Yuyun MF, Khaw KT, Luben R, Welch A, Bingham S, Day NE, Wareham NJ. A prospective study of microalbuminuria and incident coronary heart disease and its prognostic significance in a british population: The epic-norfolk study. American Journal of Epidemiology 2004;159(3):284-93. | A |
| 1. Zagaceta J, Bastarrika G, Zulueta JJ, Colina I, Alcaide AB, Campo A, et al. Prospective comparison of non-invasive risk markers of major cardiovascular events in COPD patients. Respiratory Research 2017;18(1). | B |
| 1. Zalawadiya SK, Gunasekaran PC, Bavishi CP, Veeranna V, Panaich S, Afonso L. Left ventricular hypertrophy and risk reclassification for coronary events in multi-ethnic adults. European Journal of Preventive Cardiology 2015;22(5):673-9. | A |
| 1. Zarich S, Luciano C, Hulford J, Abdullah A. Prevalence of metabolic syndrome in young patients with acute MI: Does the framingham risk score underestimate cardiovascular risk in this population? Diabetes and Vascular Disease Research 2006;3(2):103-7. | A |
| 1. Zgibor JC, Piatt GA, Ruppert K, Orchard TJ, Roberts MS. Deficiencies of cardiovascular risk prediction models for type 1 diabetes. Diabetes Care 2006;29(8):1860-5. | B |
| 1. Zhang A, Qi L, Zhang Y, Ren Z, Zhao C, Wang Q, et al. Development of a prediction model to estimate the 5-year risk of cardiovascular events and all-cause mortality in haemodialysis patients: Aretrospective study. PeerJ 2022;10. | B |
| 1. Zhang L, Niu M, Zhang H, Wang Y, Mao Z, Zhang X, et al. Nonlaboratory-based risk assessment model for coronary heart disease screening: Model development and validation. International Journal of Medical Informatics 2022;162. | A |
| 1. Zhang XL, Wan G, Yuan MX, Yang GR, Fu HJ, Zhu LX, et al. Improved framingham risk scores of patients with type 2 diabetes mellitus in the beijing community: A 10-year prospective study of the effects of multifactorial interventions on cardiovascular risk factors (the beijing communities diabetes study 22). Diabetes Therapy 2020;11(4):885-903. | A |
| 1. Zheng L, Li J, Hu D, Luo Y, Li X, Xu Y, et al. Association of low ankle-brachial index with mortality in patients with ischemic heart disease. Journal of Atherosclerosis and Thrombosis 2010;17(7):759-67. | A |
| 1. Zhou QM, Zheng Y, Cai T. Subgroup specific incremental value of new markers for risk prediction. Lifetime Data Analysis 2013;19(2):142-69. | A |
| 1. Zhu Z, Liu Y, Zhang C, Yuan Z, Zhang Q, Tang F, et al. Identification of cardiovascular risk components in urban chinese with metabolic syndrome and application to coronary heart disease prediction: A longitudinal study. PLoS ONE 2013;8(12). | A |
| 1. Ziegler L, Frumento P, Wallén H, de Faire U, Gigante B. The predictive role of interleukin 6 trans-signalling in middle-aged men and women at low-intermediate risk of cardiovascular events. European Journal of Preventive Cardiology 2020;27(2):122-9. | C |
| 1. Zuo Y, Han X, Tian X, Chen S, Wu S, Wang A. Association of impaired fasting glucose with cardiovascular disease in the absence of risk factor. Journal of Clinical Endocrinology and Metabolism 2022;107(4):E1710-8. | C |

A: Did not validate a Framingham coronary heart disease (CHD) risk rule.

B: Evaluated Framingham models other than the Wilson CHD risk rule.

C: Assessed outcomes other than CHD or hard CHD.

D: Had a follow-up duration less than 5 years.
