# Supplementary material for: Research inefficiencies in external validation studies of the Framingham Wilson coronary heart disease risk rule: A systematic review
Source: PLoS One. 2024 Sep 13;19(9):e0310321. doi: 10.1371/journal.pone.0310321 (PMC12140082; doi:10.1371/journal.pone.0310321)
Supplement: S3 Table — (DOCX) [file pone.0310321.s003.docx]

## S7 Table. Journals that published the included studies of the Framingham Wilson coronary heart disease risk rule.

| Number of studies | Journals that published external validation study of Framingham Wilson coronary heart disease risk rule |
| --- | --- |
| 3 | Medicina Clinica |
| 2 | American Journal of Cardiology, Diabetic Care, European Heart Journal, Methods of Information in Medicine, PLoS ONE |
| 1 | American Heart Journal, Annals of Family Medicine, Annals of Internal Medicine, Archives of Internal Medicine, Atención Primaria, BMC Cardiovascular Disorders, Cardiology, Circulation Journal, Diabetic Medicine, European Journal of Preventive Cardiology, HIV Clinical Trials, Hypertension Research, International Journal of Epidemiology, Journal of Atherosclerosis and Thrombosis, Journal of Epidemiology and Community Health, Kidney International, Liver International, Liver Transplantation, Medical Journal of Australia, Preventing Chronic Disease, Renal Failure, Revista Colombiana de Cardiología, Revista Española de Cardiología, Revista Española de Salud Pública, Salud Pública de México, Scandinavian Journal of Primary Health Care |

| Number of studies | Journals that published studies with a performance measure of Framingham Wilson coronary heart disease risk rule |
| --- | --- |
| 14 | Journal of the American Medical Association |
| 5 | Journal of the American College of Cardiology |
| 3 | Circulation, European Heart Journal, European Journal of Preventive Cardiology, Heart |
| 2 | American Heart Journal, American Journal of Cardiology, Atherosclerosis |
| 1 | American Journal of Medicine, Annals of Internal Medicine, Arteriosclerosis Thrombosis Vascular Biology, British Journal of General Practice, Circulation-Cardiovascular Genetics, Diabetes, Diabetic Care, Diabetic Medicine, Frontiers in Genetics, Genetic Epidemiology, Growth Factors, International Journal of Cardiovascular Imaging, JACC-Cardiovascular Imaging, Journal of Cardiovascular Magnetic Resonance, Journal of Human Hypertension, Kidney International, Nefrologia, PLoS ONE, Preventive Medicine Reports, Revista Española de Cardiología, Scientific Reports |
