# Supplementary material for: Research inefficiencies in external validation studies of the Framingham Wilson coronary heart disease risk rule: A systematic review
Source: PLoS One. 2024 Sep 13;19(9):e0310321. doi: 10.1371/journal.pone.0310321 (PMC12140082; doi:10.1371/journal.pone.0310321)
Supplement: S4 Table — (DOCX) [file pone.0310321.s004.docx]

## S8 Table. Studies that derived a new cardiovascular CPR using the poor performance of the Framingham Wilson coronary heart disease risk rule in the external validation study as a justification.

| External validation study | Derivation study of a new cardiovascular CPR |
| --- | --- |
| Cañón-Barroso et al. (1) | Su X, Xu Y, Tan Z, Wang X, Yang P, Su Y, et al. Prediction for cardiovascular diseases based on laboratory data: An analysis of random forest model. J Clin Lab Anal. 2020;34(9):e23421. |
| Chien et al. (2) | Jamthikar A, Gupta D, Khanna NN, Saba L, Laird JR, Suri JS. Cardiovascular/stroke risk prevention: A new machine learning framework integrating carotid ultrasound image-based phenotypes and its harmonics with conventional risk factors. Indian Heart J. 2020;72(4):258-64. |
| Herrera et al. (3) | De Socio GV, Pucci G, Baldelli F, Schillaci G. Observed versus predicted cardiovascular events and all-cause death in HIV infection: a longitudinal cohort study. BMC Infect Dis. 2017;17(1):414. |
| Comín et al. (4) | Ramos R, Baena-Diez JM, Quesada M, Solanas P, Subirana I, Sala J, et al. Derivation and validation of REASON: a risk score identifying candidates to screen for peripheral arterial disease using ankle brachial index. Atherosclerosis. 2011;214(2):474-9. |
| Davis et al. (5) | Davis WA, Knuiman MW, Davis TM. An Australian cardiovascular risk equation for type 2 diabetes: the Fremantle Diabetes Study. Intern Med J. 2010;40(4):286-92. |
| DeFilippis et al. (6) | Cho IJ, Sung JM, Chang HJ, Chung N, Kim HC. Incremental Value of Repeated Risk Factor Measurements for Cardiovascular Disease Prediction in Middle-Aged Korean Adults: Results From the NHIS-HEALS (National Health Insurance System-National Health Screening Cohort). Circ Cardiovasc Qual Outcomes. 2017;10(11).  Blaha MJ, Naazie IN, Cainzos-Achirica M, Dardari ZA, Defilippis AP, McClelland RL, et al. Derivation of a coronary age calculator using traditional risk factors and coronary artery calcium: The multi-ethnic study of atherosclerosis. Journal of the American Heart Association. 2021;10(6). |
| Ducloux et al. (7) | Israni AK, Snyder JJ, Skeans MA, Peng Y, Maclean JR, Weinhandl ED, et al. Predicting coronary heart disease after kidney transplantation: Patient Outcomes in Renal Transplantation (PORT) Study. Am J Transplant. 2010;10(2):338-53.  Pita-Fernandez S, Pertega-Diaz S, Valdes-Canedo F, Seijo-Bestilleiro R, Seoane-Pillado T, Fernandez-Rivera C, et al. Incidence of cardiovascular events after kidney transplantation and cardiovascular risk scores: study protocol. BMC Cardiovasc Disord. 2011;11:2. |
| Empana et al. (8) | Balkau B, Hu G, Qiao Q, Tuomilehto J, Borch-Johnsen K, Pyorala K, et al. Prediction of the risk of cardiovascular mortality using a score that includes glucose as a risk factor. The DECODE Study. Diabetologia. 2004;47(12):2118-28.  Barzi F, Patel A, Gu D, Sritara P, Lam TH, Rodgers A, et al. Cardiovascular risk prediction tools for populations in Asia. J Epidemiol Community Health. 2007;61(2):115-21.  Cournot M, Taraszkiewicz D, Cambou JP, Galinier M, Boccalon H, Hanaire-Broutin H, et al. Additional prognostic value of physical examination, exercise testing, and arterial ultrasonography for coronary risk assessment in primary prevention. American heart journal. 2009;158(5):845-51.  Empana JP, Tafflet M, Escolano S, Vergnaux AC, Bineau S, Ruidavets JB, et al. Predicting CHD risk in France: a pooled analysis of the D.E.S.I.R., Three City, PRIME, and SU.VI.MAX studies. Eur J Cardiovasc Prev Rehabil. 2011;18(2):175-85.  Ferrario M, Chiodini P, Chambless LE, Cesana G, Vanuzzo D, Panico S, et al. Prediction of coronary events in a low incidence population. Assessing accuracy of the CUORE Cohort Study prediction equation. Int J Epidemiol. 2005;34(2):413-21. |
| Guckelberger et al. (9) | Moody WE, Holloway B, Arumugam P, Gill S, Wahid YS, Boivin CM, et al. Prognostic value of coronary risk factors, exercise capacity and single photon emission computed tomography in liver transplantation candidates: A 5-year follow-up study. J Nucl Cardiol. 2020. |
| Marrugat et al. (10) | Empana JP, Tafflet M, Escolano S, Vergnaux AC, Bineau S, Ruidavets JB, et al. Predicting CHD risk in France: a pooled analysis of the D.E.S.I.R., Three City, PRIME, and SU.VI.MAX studies. Eur J Cardiovasc Prev Rehabil. 2011;18(2):175-85.  Huerta JM, Tormo MJ, Gavrila D, Navarro C. Cardiovascular risk estimated after 13 years of follow-up in a low-incidence Mediterranean region with high-prevalence of cardiovascular risk factors. BMC Public Health. 2010;10:640. |
| Van der Heijden et al. (11) | Magri CJ, Debono R, Calleja N, Galea J, Fava S. Prognostic indicators and generation of novel risk equations for estimation of 10-year and 20-year mortality following acute coronary syndrome. Postgrad Med J. 2017;93(1099):245-9.  Price AH, Weir CJ, Welsh P, McLachlan S, Strachan MWJ, Sattar N, et al. Comparison of non-traditional biomarkers, and combinations of biomarkers, for vascular risk prediction in people with type 2 diabetes: The Edinburgh Type 2 Diabetes Study. Atherosclerosis. 2017;264:67-73.  Willis M, Asseburg C, Slee A, Nilsson A, Neslusan C. Macrovascular Risk Equations Based on the CANVAS Program. PharmacoEconomics. 2021;39(4):447-61. |

1. Canon-Barroso L, Muro EC, Herrera ND, Ochoa GF, Hueros JI, Buitrago F. Performance of the Framingham and SCORE cardiovascular risk prediction functions in a non-diabetic population of a Spanish health care centre: a validation study. Scandinavian journal of primary health care. 2010;28(4):242-8.

2. Chien KL, Lin HJ, Su TC, Chen YY, Chen PC. Comparing the Consistency and Performance of Various Coronary Heart Disease Prediction Models for Primary Prevention Using a National Representative Cohort in Taiwan. Circ J. 2018;82(7):1805-12.

3. Herrera S, Guelar A, Sorli L, Vila J, Molas E, Grau M, et al. The Framingham function overestimates the risk of ischemic heart disease in HIV-infected patients from Barcelona. HIV Clin Trials. 2016;17(4):131-9.

4. Comin E, Solanas P, Cabezas C, Subirana I, Ramos R, Gene-Badia J, et al. [Estimating cardiovascular risk in Spain using different algorithms]. Rev Esp Cardiol. 2007;60(7):693-702.

5. Davis WA, Colagiuri S, Davis TM. Comparison of the Framingham and United Kingdom Prospective Diabetes Study cardiovascular risk equations in Australian patients with type 2 diabetes from the Fremantle Diabetes Study. Med J Aust. 2009;190(4):180-4.

6. DeFilippis AP, Young R, Carrubba CJ, McEvoy JW, Budoff MJ, Blumenthal RS, et al. An analysis of calibration and discrimination among multiple cardiovascular risk scores in a modern multiethnic cohort. Annals of internal medicine. 2015;162(4):266-75.

7. Ducloux D, Kazory A, Chalopin JM. Predicting coronary heart disease in renal transplant recipients: a prospective study. Kidney Int. 2004;66(1):441-7.

8. Empana JP, Ducimetiere P, Arveiler D, Ferrieres J, Evans A, Ruidavets JB, et al. Are the Framingham and PROCAM coronary heart disease risk functions applicable to different European populations? The PRIME Study. European heart journal. 2003;24(21):1903-11.

9. Guckelberger O, Mutzke F, Glanemann M, Neumann UP, Jonas S, Neuhaus R, et al. Validation of cardiovascular risk scores in a liver transplant population. Liver Transpl. 2006;12(3):394-401.

10. Marrugat J, Subirana I, Comin E, Cabezas C, Vila J, Elosua R, et al. Validity of an adaptation of the Framingham cardiovascular risk function: the VERIFICA Study. J Epidemiol Community Health. 2007;61(1):40-7.

11. van der Heijden AA, Ortegon MM, Niessen LW, Nijpels G, Dekker JM. Prediction of coronary heart disease risk in a general, pre-diabetic, and diabetic population during 10 years of follow-up: accuracy of the Framingham, SCORE, and UKPDS risk functions: The Hoorn Study. Diabetes care. 2009;32(11):2094-8.
