# Supplementary material for: Research inefficiencies in external validation studies of the Framingham Wilson coronary heart disease risk rule: A systematic review
Source: PLoS One. 2024 Sep 13;19(9):e0310321. doi: 10.1371/journal.pone.0310321 (PMC12140082; doi:10.1371/journal.pone.0310321)
Supplement: S1 Appendix — (DOCX) [file pone.0310321.s005.docx]

S2 Appendix. Methods for meta-analysis of predicted and observed (P/O) ratio and *c* statistic

1. Calculation of the predicted and observed number of 10-year coronary heart disease (CHD) events

The Framingham Wilson CHD risk rule predicts a 10-year risk of CHD event. However, many studies reported outcomes with follow-up durations different from 10 years. For these studies, we used the following methods [1] to calculate the number of CHD events anticipated with a 10-year follow-up.

Firstly, a cumulative incidence (CI) was calculated by,

|  | $CI =\frac{Number of CHD events}{Number of participants}$ | (1) |
| --- | --- | --- |

Then, an incidence rate (IR) was determined using the following equation,

|  | $IR =-\frac{1}{T} ln(1-CI)$ | (2) |
| --- | --- | --- |

where $T$ is the follow-up duration. Based on the calculated incidence rate, a 10-year cumulative incidence is calculated by,

|  | $10 year CI=1-e^{(-IR\times10)}$ | (3) |
| --- | --- | --- |

Lastly, the number of CHD events anticipated with a 10-year follow-up was calculated by multiplying the number of total participants and the 10-year cumulative incidence.

The number of CHD outcomes predicted by the Framingham Wilson CHD risk rule was calculated by multiplying the number of participants and predicted risk. When studies presented total points assessed by the Framingham Wilson CHD rule, we used the corresponding risk reported by Wilson et al. [2] to calculate the predicted number of CHD outcomes (e.g. 10 points based on LDL cholesterol for men corresponds to 27% 10-year risk).

Example: *A study by Fowkes et al. [3] included the ARIC cohorts with a total sample size of 14,109 participants: 6105 men with 571 CHD events during a 13.1-year median follow-up and 8,004 women with 362 CHD events during a 13.2-year median follow-up. Cumulative incidence calculated using equation (1) was 0.093529894 for men and 0.045227386 for women. The corresponding incidence rate estimated by equation (2) was 0.007495971 for men and 0.03445462 for women. Similarly, the 10-year cumulative incidence was 0.072219138 for men and 0.03445462 for women, based on equation (3). Lastly, by multiplying the number of participants by the 10-year cumulative incidence, the anticipated number of 10-year CHD events was 441 for men and 276 for women. Therefore, the total number of 10-year CHD events was 717 for the ARIC cohort.*

| Group | Sample size | Number of events | Follow up, year | Cumulative incidence | Incidence rate | 10-year cumulative incidence | 10-year number of event |
| --- | --- | --- | --- | --- | --- | --- | --- |
| Men | 6,105 | 571 | 13.1 | 0.093529894 | 0.007495971 | 0.072219138 | 441 |
| Women | 8,004 | 362 | 13.2 | 0.045227386 | 0.003506217 | 0.03445462 | 276 |
| **Total** | **14,109** |  |  |  |  |  | **717** |

*Since the 10-year CHD risk estimated by the Framingham Wilson CHD risk rule was 12.8% for men and 7.3% for women, the number of predicted CHD events was 781 for men and 584 for women, with a total number of CHD events of 1,366.*

1. Meta-analysis

To conduct meta-analyses, we used the methods described by Debray et al. [4]. Here, we briefly summarise how we adopted and applied some of their methods in our analyses.

1. Meta-analysis of P/O ratio

Because the formulas suggested by Derbray et al. [4] rely on Observed/Predicted (O/P) ratios, we converted P/O ratios to O/P ratios. Then, the O/P ratios were transformed to a natural logarithmic scale, $ln(O/P)$. The standard error of $ln(O/P)$ was calculated using the next equation.

|  | $SE of\ln(O/{P)}=\sqrt{\frac{1-observed 10 year risk of CHD event}{CHD event observed in 10 years}}$ | (4) |
| --- | --- | --- |

After conducting random effects meta-analysis using $ln(O/P)$ and $SE of\ln(O/{P)}$, the results were transformed and converted back to P/O ratios and corresponding 95% confidence intervals.

*Example: On the ARIC cohort from the study by Fowkes et al. [3], the 10-year predicted and observed CHD events among 14,109 participants were 1,366 and 717, respectively. Therefore, the P/O and O/P ratios can be calculated by 1366/717 = 1.905 and by 717/1366 = 0.525. The* $ln(O/P)$*calculated using the equation (4) was -0.6445. The* $SE of\ln(O/{P)}$ *was determined based on equation (4) was 0.036, with a corresponding 95% confidence interval for the* $ln(O/P)$*of* -0.716 - -0.573*. After meta-analysing, the* $ln(O/P)$ *and its 95% confidence interval were transformed and converted back to the P/O ratio and 95% confidence interval of 1.905 and 1.773 - 2.045, as presented in Fig. 3-A.*

1. Meta-analysis of *c* statistic

As described by Debray et al. [4], *c* statistics were logit transformed simply by,

|  | $logit c=\ln\left( \frac{c}{1-c} \right)$ | (5) |
| --- | --- | --- |

The standard error of $logit c$ was obtained using the following equation,

|  | $SE of logit c=\sqrt{\frac{1+n^{*}\frac{1-c}{2-c}+\frac{m^{*}c}{1+c}}{mnc(1-c)}}$ | (5) |
| --- | --- | --- |

where $n$ is the number of participants with CHD events, $m$ is the number of participants without CHF events, and $n^{*}= m^{*}=\frac{1}{2}\left( n+m \right)-1.$When *c* statistics were reported for multiple groups (e.g., male and female), a weighted average of them was used for each study.

After conducting random effects meta-analysis using $logit c$ and $SE oflogit c$, the results were transformed and converted back to *c* statistics and corresponding 95% confidence intervals.

*Example: A study by Orford et al. [5] reported the c statistic of the Framingham Wilson CHD risk rule was 0.630. There were 1,393 participants, 206 with CHD and 1126 without CHD.* $logit c$ *and* $SE of logit c$ *calculated using equations (5) and (6) were 0.532 and 0.0896, respectively. After meta-analysing, these were converted to c statistic and 95% confidence interval of 0.630 and 0.588 - 0.670.*

**References**

1. Szklo M, Szklo M. Epidemiology : beyond the basics. 3rd ed. Nieto FJ, editor. Burlington, Mass: Jones & Bartlett Learning; 2014.

2. Wilson PW, D'Agostino RB, Levy D, Belanger AM, Silbershatz H, Kannel WB. Prediction of coronary heart disease using risk factor categories. Circulation. 1998;97(18):1837-47. PubMed PMID: 9603539.

3. Ankle Brachial Index C, Fowkes FG, Murray GD, Butcher I, Heald CL, Lee RJ, et al. Ankle brachial index combined with Framingham Risk Score to predict cardiovascular events and mortality: a meta-analysis. JAMA : the journal of the American Medical Association. 2008;300(2):197-208. doi: 10.1001/jama.300.2.197. PubMed PMID: 18612117; PubMed Central PMCID: PMCPMC2932628.

4. Debray TP, Damen JA, Riley RD, Snell K, Reitsma JB, Hooft L, et al. A framework for meta-analysis of prediction model studies with binary and time-to-event outcomes. Statistical methods in medical research. 2019;28(9):2768-86. Epub 20180723. doi: 10.1177/0962280218785504. PubMed PMID: 30032705; PubMed Central PMCID: PMCPMC6728752.

5. Orford JL, Sesso HD, Stedman M, Gagnon D, Vokonas P, Gaziano JM. A comparison of the Framingham and European Society of Cardiology coronary heart disease risk prediction models in the normative aging study. American heart journal. 2002;144(1):95-100. doi: 10.1067/mhj.2002.123317. PubMed PMID: 12094194.
