# Supplementary material for: Research inefficiencies in external validation studies of the Framingham Wilson coronary heart disease risk rule: A systematic review
Source: PLoS One. 2024 Sep 13;19(9):e0310321. doi: 10.1371/journal.pone.0310321 (PMC12140082; doi:10.1371/journal.pone.0310321)
Supplement: S3 Appendix — (DOCX) [file pone.0310321.s007.docx]

S1 Appendix. List of included articles of the Framingham Wilson coronary heart disease (CHD) risk rule

**A. Articles with studies that aimed to externally validate the Framingham Wilson CHD risk rule: “external validation studies”**

1. Beaney KE, Cooper JA, Ullah Shahid S, Ahmed W, Qamar R, Drenos F, et al. Clinical Utility of a Coronary Heart Disease Risk Prediction Gene Score in UK Healthy Middle Aged Men and in the Pakistani Population. PLoS One. 2015;10(7):e0130754.

2. Becker A, Leber AW, Becker C, von Ziegler F, Tittus J, Schroeder I, et al. Predictive value of coronary calcifications for future cardiac events in asymptomatic patients with diabetes mellitus: a prospective study in 716 patients over 8 years. BMC Cardiovasc Disord. 2008;8:27.

3. Brunner EJ, Shipley MJ, Marmot MG, Kivimaki M, Witte DR. Do the Joint British Society (JBS2) guidelines on prevention of cardiovascular disease with respect to plasma glucose improve risk stratification in the general population? Prospective cohort study. Diabetic Medicine. 2010;27(5):550-5.

4. Buitrago F, Calvo-Hueros JI, Gomez-Jimenez C, Canon-Barroso L, Suarez-Gonzalez F, Robles NR. Hidden chronic renal insufficiency and cardiovascular events in patients with hypertension in a primary care center. Ren Fail. 2010;32(7):757-65.

5. Buitrago F, Calvo-Hueros JI, Canon-Barroso L, Pozuelos-Estrada G, Molina-Martinez L, Espigares-Arroyo M, et al. Original and REGICOR Framingham functions in a nondiabetic population of a Spanish health care center: a validation study. Annals of family medicine. 2011;9(5):431-8.

6. Calvo-Hueros JI, Canon-Barroso L, Morales-Gabardino JA, Buitrago F. Cardiovascular risk and validation of cardiovascular risk prediction functions in a cohort of patients with type 2 diabetes followed for 10 years in Badajoz (SPAIN). AN observational study. Prim Care Diabetes. 2021;15(1):115-20.

7. Calvo Hueros JI, Canon Barroso L, Gomez Jimenez C, Martin Hidalgo-Barquero MV, Espigares Arroyo M, Buitrago Ramirez F. [Cardiovascular risk in patients with renal failure]. Med Clin (Barc). 2008;131(2):41-6.

8. Canon-Barroso L, Cruces-Muro E, Fernandez-Ochoa G, Nieto-Hernandez T, Garcia-Vellido A, Buitrago F. [Validation of 3 equations of coronary risk in diabetic population of a primary care center]. Med Clin (Barc). 2006;126(13):485-90.

9. Canon-Barroso L, Diaz Herrera N, Calvo Hueros JI, Cruces Muro E, Nieto Hernandez T, Buitrago Ramirez F. [Incidence of cardiovascular disease and validity of equations of coronary risk in diabetic patients with metabolic syndrome]. Med Clin (Barc). 2007;128(14):529-35.

10. Canon-Barroso L, Diaz Herrera N, Cruces Muro E, Nieto Hernandez T, Garrote Florencio T, Buitrago F. [Predictive capacity, comparison and clinic consequences of the Framingham-Wilson and REGICOR tables in persons for whom care has been provided at a health care center, Badajoz, Spain]. Rev Esp Salud Publica. 2007;81(4):353-64.

11. Canon-Barroso L, Muro EC, Herrera ND, Ochoa GF, Hueros JI, Buitrago F. Performance of the Framingham and SCORE cardiovascular risk prediction functions in a non-diabetic population of a Spanish health care centre: a validation study. Scandinavian journal of primary health care. 2010;28(4):242-8.

12. Chien KL, Lin HJ, Su TC, Chen YY, Chen PC. Comparing the Consistency and Performance of Various Coronary Heart Disease Prediction Models for Primary Prevention Using a National Representative Cohort in Taiwan. Circ J. 2018;82(7):1805-12.

13. Comin E, Solanas P, Cabezas C, Subirana I, Ramos R, Gene-Badia J, et al. [Estimating cardiovascular risk in Spain using different algorithms]. Rev Esp Cardiol. 2007;60(7):693-702.

14. Davis WA, Colagiuri S, Davis TM. Comparison of the Framingham and United Kingdom Prospective Diabetes Study cardiovascular risk equations in Australian patients with type 2 diabetes from the Fremantle Diabetes Study. Med J Aust. 2009;190(4):180-4.

15. DeFilippis AP, Young R, Carrubba CJ, McEvoy JW, Budoff MJ, Blumenthal RS, et al. An analysis of calibration and discrimination among multiple cardiovascular risk scores in a modern multiethnic cohort. Annals of internal medicine. 2015;162(4):266-75.

16. Ducloux D, Kazory A, Chalopin JM. Predicting coronary heart disease in renal transplant recipients: a prospective study. Kidney Int. 2004;66(1):441-7.

17. Empana JP, Ducimetiere P, Arveiler D, Ferrieres J, Evans A, Ruidavets JB, et al. Are the Framingham and PROCAM coronary heart disease risk functions applicable to different European populations? The PRIME Study. Eur Heart J. 2003;24(21):1903-11.

18. Ferrario M, Chiodini P, Chambless LE, Cesana G, Vanuzzo D, Panico S, et al. Prediction of coronary events in a low incidence population. Assessing accuracy of the CUORE Cohort Study prediction equation. Int J Epidemiol. 2005;34(2):413-21.

19. Gander J, Sui X, Hazlett LJ, Cai B, Hébert JR, Blair SN. Factors related to coronary heart disease risk among men: validation of the Framingham Risk Score. Preventing chronic disease. 2014;11:E140.

20. Guckelberger O, Mutzke F, Glanemann M, Neumann UP, Jonas S, Neuhaus R, et al. Validation of cardiovascular risk scores in a liver transplant population. Liver Transpl. 2006;12(3):394-401.

21. Herrera S, Guelar A, Sorli L, Vila J, Molas E, Grau M, et al. The Framingham function overestimates the risk of ischemic heart disease in HIV-infected patients from Barcelona. HIV Clin Trials. 2016;17(4):131-9.

22. Jimenez-Corona A, Lopez-Ridaura R, Williams K, Gonzalez-Villalpando ME, Simon J, Gonzalez-Villalpando C. Applicability of Framingham risk equations for studying a low-income Mexican population. Salud Publica Mex. 2009;51(4):298-305.

23. Jimeno Mollet J, Molist Brunet N, Franch Nadal J, Serrano Borraz V, Serrano Barragan L, Gracia Gimenez R. [Variability in the calculation of coronary risk in type-2 diabetes mellitus]. Aten Primaria. 2005;35(1):30-6.

24. Mainous AG, 3rd, Koopman RJ, Diaz VA, Everett CJ, Wilson PW, Tilley BC. A coronary heart disease risk score based on patient-reported information. Am J Cardiol. 2007;99(9):1236-41.

25. Marrugat J, Subirana I, Comin E, Cabezas C, Vila J, Elosua R, et al. Validity of an adaptation of the Framingham cardiovascular risk function: the VERIFICA Study. J Epidemiol Community Health. 2007;61(1):40-7.

26. Merry AH, Boer JM, Schouten LJ, Ambergen T, Steyerberg EW, Feskens EJ, et al. Risk prediction of incident coronary heart disease in The Netherlands: re-estimation and improvement of the SCORE risk function. European journal of preventive cardiology. 2012;19(4):840-8.

27. Muñoz OM, Rodríguez NI, Ruiz Á, Rondón M. Validación de los modelos de predicción de Framingham y PROCAM como estimadores del riesgo cardiovascular en una población colombiana. Rev Colomb Cardiol. 2014;21(4):202-12.

28. Nishimura K, Okamura T, Watanabe M, Nakai M, Takegami M, Higashiyama A, et al. Predicting Coronary Heart Disease Using Risk Factor Categories for a Japanese Urban Population, and Comparison with the Framingham Risk Score: The Suita Study. Journal of Atherosclerosis and Thrombosis. 2014;21(8):784-98.

29. Onat A, Can G, Hergenc G, Kucukdurmaz Z, Ugur M, Yuksel H. High absolute coronary disease risk among Turks: involvement of risk factors additional to conventional ones. Cardiology. 2010;115(4):297-306.

30. Orford JL, Sesso HD, Stedman M, Gagnon D, Vokonas P, Gaziano JM. A comparison of the Framingham and European Society of Cardiology coronary heart disease risk prediction models in the normative aging study. Am Heart J. 2002;144(1):95-100.

31. Protopsaltis ID, Konstantinopoulos PA, Kamaratos AV, Melidonis AI. Comparative study of prognostic value for coronary disease risk between the U.K. prospective diabetes study and Framingham models. Diabetes care. 2004;27(1):277-8.

32. Reissigova J, Zvarova J. The Framingham risk function underestimated absolute coronary heart disease risk in Czech men. Methods Inf Med. 2007;46(1):43-9.

33. Rodondi N, Locatelli I, Aujesky D, Butler J, Vittinghoff E, Simonsick E, et al. Framingham risk score and alternatives for prediction of coronary heart disease in older adults. PLoS One. 2012;7(3):e34287.

34. Simmons RK, Sharp S, Boekholdt SM, Sargeant LA, Khaw K-T, Wareham NJ, et al. Evaluation of the Framingham Risk Score in the European Prospective Investigation of Cancer–Norfolk Cohort: Does Adding Glycated Hemoglobin Improve the Prediction of Coronary Heart Disease Events? Archives of internal medicine (1960). 2008;168(11):1209-16.

35. Suka M, Sugimori H, Yoshida K. Application of the updated Framingham risk score to Japanese men. Hypertens Res. 2001;24(6):685-9.

36. Suka M, Sugimori H, Yoshida K. Validity of the Framingham risk model applied to Japanese men. Methods Inf Med. 2002;41(3):213-5.

37. Treeprasertsuk S, Leverage S, Adams LA, Lindor KD, Sauver J, Angulo P. The Framingham risk score and heart disease in nonalcoholic fatty liver disease. Liver International. 2012;32(6):945-50.

38. Vaidya D, Yanek LR, Moy TF, Pearson TA, Becker LC, Becker DM. Incidence of Coronary Artery Disease in Siblings of Patients With Premature Coronary Artery Disease: 10 Years of Follow-up. The American journal of cardiology. 2007;100(9):1410-5.

39. van der Heijden AA, Ortegon MM, Niessen LW, Nijpels G, Dekker JM. Prediction of coronary heart disease risk in a general, pre-diabetic, and diabetic population during 10 years of follow-up: accuracy of the Framingham, SCORE, and UKPDS risk functions: The Hoorn Study. Diabetes care. 2009;32(11):2094-8.

**B. Articles with studies in which a performance measure of the Framingham Wilson CHD risk rule could be obtained**

1. Baena-Diez JM, Vidal-Solsona M, Byram AO, Gonzalez-Casafont I, Ledesma-Ulloa G, Marti-Sans N. The epidemiology of cardiovascular disease in primary care. the Zona Franca Cohort study in Barcelona, Spain. Rev Esp Cardiol. 2010;63(11):1261-9.

2. Barbier CE, Themudo R, Bjerner T, Johansson L, Lind L, Ahlstrom H. Long-term prognosis of unrecognized myocardial infarction detected with cardiovascular magnetic resonance in an elderly population. J Cardiovasc Magn Reson. 2016;18(1):43.

3. Blake DR, Meigs JB, Muller DC, Najjar SS, Andres R, Nathan DM. Impaired glucose tolerance, but not impaired fasting glucose, is associated with increased levels of coronary heart disease risk factors: results from the Baltimore Longitudinal Study on Aging. Diabetes. 2004;53(8):2095-100.

4. Buitrago F, Calvo JI, Gomez-Jimenez C, Canon L, Robles NR, Angulo E. [Comparison and agreement of the Cockcroft-Gault and MDRD equations to estimate glomerular filtration rate in diagnosis of occult chronic kidney disease]. Nefrologia. 2008;28(3):301-10.

5. Buitrago F, Calvo JI, Redondo-Lopez V, Canon-Barroso L, Rodriguez-Perez L, Hinojosa-Diaz JF. Cardiovascular events in patients with obesity: an observational study. The British journal of general practice : the journal of the Royal College of General Practitioners. 2010;60(577):584-9.

6. Chang SM, Nabi F, Xu J, Peterson LE, Achari A, Pratt CM, et al. The coronary artery calcium score and stress myocardial perfusion imaging provide independent and complementary prediction of cardiac risk. Journal of the American College of Cardiology. 2009;54(20):1872-82.

7. Corona AJ, Martinez DR, Avila MH, Haffner S, Williams K, Gonzalez Villalpando ME, et al. Microalbuminuria as a predictor of myocardial infarction in a Mexican population: the Mexico City Diabetes Study. Kidney Int Suppl. 2005(97):S34-9.

8. Denes P, Larson JC, Lloyd-Jones DM, Prineas RJ, Greenland P. Major and minor ECG abnormalities in asymptomatic women and risk of cardiovascular events and mortality. JAMA : the journal of the American Medical Association. 2007;297(9):978-85.

9. Drawz PE, Baraniuk S, Davis BR, Brown CD, Colon PJ, Sr., Cujyet AB, et al. Cardiovascular risk assessment: addition of CKD and race to the Framingham equation. American heart journal. 2012;164(6):925-31 e2.

10. Ensrud K, LaCroix A, Thompson JR, Thompson DD, Eastell R, Reid DM, et al. Lasofoxifene and cardiovascular events in postmenopausal women with osteoporosis: Five-year results from the Postmenopausal Evaluation and Risk Reduction with Lasofoxifene (PEARL) trial. Circulation. 2010;122(17):1716-24.

11. Erbel R, Mohlenkamp S, Moebus S, Schmermund A, Lehmann N, Stang A, et al. Coronary risk stratification, discrimination, and reclassification improvement based on quantification of subclinical coronary atherosclerosis: the Heinz Nixdorf Recall study. Journal of the American College of Cardiology. 2010;56(17):1397-406.

12. Forés R, Alzamora MT, Pera G, Baena-Diez JM, Mundet-Tuduri X, Toran P. Contribution of the ankle-brachial index to improve the prediction of coronary risk: The ARTPER cohort. PloS one. 2018;13(1):e0191283.

13. Fowkes FG, Murray GD, Butcher I, Heald CL, Lee RJ, Chambless LE, et al. Ankle brachial index combined with Framingham Risk Score to predict cardiovascular events and mortality: a meta-analysis. JAMA : the journal of the American Medical Association. 2008;300(2):197-208.

14. Fowkes FG, Murray GD, Butcher I, Folsom AR, Hirsch AT, Couper DJ, et al. Development and validation of an ankle brachial index risk model for the prediction of cardiovascular events. European journal of preventive cardiology. 2014;21(3):310-20.

15. Gander JC, Sui X, Hebert JR, Lavie CJ, Hazlett LJ, Cai B, et al. Addition of estimated cardiorespiratory fitness to the clinical assessment of 10-year coronary heart disease risk in asymptomatic men. Prev Med Rep. 2017;7:30-7.

16. Gerritsen KG, Falke LL, van Vuuren SH, Leeuwis JW, Broekhuizen R, Nguyen TQ, et al. Plasma CTGF is independently related to an increased risk of cardiovascular events and mortality in patients with atherosclerotic disease: the SMART study. Growth Factors. 2016;34(3-4):149-58.

17. Goldstein BA, Knowles JW, Salfati E, Ioannidis JP, Assimes TL. Simple, standardized incorporation of genetic risk into non-genetic risk prediction tools for complex traits: coronary heart disease as an example. Front Genet. 2014;5:254.

18. Goldstein BA, Yang L, Salfati E, Assimes TL. Contemporary Considerations for Constructing a Genetic Risk Score: An Empirical Approach. Genet Epidemiol. 2015;39(6):439-45.

19. Grossman C, Ehrlich S, Shemesh J, Koren-Morag N, Grossman E. Coronary artery calcium and exercise electrocardiogram as predictors of coronary events in asymptomatic adults. Am J Cardiol. 2015;115(6):745-50.

20. Heidari B, Nargesi AA, Hafezi-Nejad N, Sheikhbahaei S, Pajouhi A, Nakhjavani M, et al. Assessment of serum 25-hydroxy vitamin D improves coronary heart disease risk stratification in patients with type 2 diabetes. American heart journal. 2015;170(3):573-9 e5.

21. Iribarren C, Lu M, Jorgenson E, Martinez M, Lluis-Ganella C, Subirana I, et al. Clinical Utility of Multimarker Genetic Risk Scores for Prediction of Incident Coronary Heart Disease: A Cohort Study Among Over 51 000 Individuals of European Ancestry. Circ Cardiovasc Genet. 2016;9(6):531-40.

22. Iribarren C, Lu M, Jorgenson E, Martinez M, Lluis-Ganella C, Subirana I, et al. Weighted Multi-marker Genetic Risk Scores for Incident Coronary Heart Disease among Individuals of African, Latino and East-Asian Ancestry. Sci Rep. 2018;8(1):6853.

23. Isaacs A, Willems SM, Bos D, Dehghan A, Hofman A, Ikram MA, et al. Risk scores of common genetic variants for lipid levels influence atherosclerosis and incident coronary heart disease. Arterioscler Thromb Vasc Biol. 2013;33(9):2233-9.

24. Kälsch H, Lehmann N, Mahabadi AA, Bauer M, Kara K, Huppe P, et al. Beyond Framingham risk factors and coronary calcification: does aortic valve calcification improve risk prediction? The Heinz Nixdorf Recall Study. Heart. 2014;100(12):930-7.

25. Kim HC, Greenland P, Rossouw JE, Manson JE, Cochrane BB, Lasser NL, et al. Multimarker prediction of coronary heart disease risk: the Women's Health Initiative. Journal of the American College of Cardiology. 2010;55(19):2080-91.

26. Koenig W, Löwel H, Baumert J, Meisinger C. C-Reactive Protein Modulates Risk Prediction Based on the Framingham Score - Implications for Future Risk Assessment: Results from a Large Cohort Study in Southern Germany. Circulation. 2004;109(11):1349-53.

27. Leistner DM, Klotsche J, Palm S, Pieper L, Stalla GK, Lehnert H, et al. Prognostic value of reported chest pain for cardiovascular risk stratification in primary care. Eur J Cardiovasc Prev Rehabil. 2014;21(6):727-38.

28. Lorenz MW, Schaefer C, Steinmetz H, Sitzer M. Is carotid intima media thickness useful for individual prediction of cardiovascular risk? Ten-year results from the Carotid Atherosclerosis Progression Study (CAPS). European heart journal. 2010;31(16):2041-8.

29. Mahabadi AA, Lehmann N, Mohlenkamp S, Pundt N, Dykun I, Roggenbuck U, et al. Noncoronary Measures Enhance the Predictive Value of Cardiac CT Above Traditional Risk Factors and CAC Score in the General Population. JACC Cardiovasc Imaging. 2016;9(10):1177-85.

30. Malik S, Budoff MJ, Katz R, Blumenthal RS, Bertoni AG, Nasir K, et al. Impact of subclinical atherosclerosis on cardiovascular disease events in individuals with metabolic syndrome and diabetes: the multi-ethnic study of atherosclerosis. Diabetes care. 2011;34(10):2285-90.

31. Meuwese MC, Stroes ES, Hazen SL, van Miert JN, Kuivenhoven JA, Schaub RG, et al. Serum myeloperoxidase levels are associated with the future risk of coronary artery disease in apparently healthy individuals: the EPIC-Norfolk Prospective Population Study. Journal of the American College of Cardiology. 2007;50(2):159-65.

32. Miyasaka Y, Barnes ME, Gersh BJ, Cha SS, Bailey KR, Seward JB, et al. Coronary ischemic events after first atrial fibrillation: risk and survival. Am J Med. 2007;120(4):357-63.

33. Mohlenkamp S, Lehmann N, Greenland P, Moebus S, Kalsch H, Schmermund A, et al. Coronary artery calcium score improves cardiovascular risk prediction in persons without indication for statin therapy. Atherosclerosis. 2011;215(1):229-36.

34. Mohlenkamp S, Lehmann N, Moebus S, Schmermund A, Dragano N, Stang A, et al. Quantification of coronary atherosclerosis and inflammation to predict coronary events and all-cause mortality. Journal of the American College of Cardiology. 2011;57(13):1455-64.

35. Mora S, Yanek LR, Moy TF, Fallin MD, Becker LC, Becker DM. Interaction of body mass index and framingham risk score in predicting incident coronary disease in families. Circulation. 2005;111(15):1871-6.

36. Nadjiri J, Hausleiter J, Deseive S, Will A, Hendrich E, Martinoff S, et al. Prognostic value of coronary CT angiography in diabetic patients: a 5-year follow up study. Int J Cardiovasc Imaging. 2016;32(3):483-91.

37. Nargesi AA, Heidari B, Esteghamati S, Hafezi-Nejad N, Sheikhbahaei S, Pajouhi A, et al. Contribution of vitamin D deficiency to the risk of coronary heart disease in subjects with essential hypertension. Atherosclerosis. 2016;244:165-71.

38. Nawrot TS, Staessen JA, Thijs L, Fagard RH, Tikhonoff V, Wang JG, et al. Should pulse pressure become part of the Framingham risk score? J Hum Hypertens. 2004;18(4):279-86.

39. Nilsson PM, Engstrom G, Hedblad B. The metabolic syndrome and incidence of cardiovascular disease in non-diabetic subjects--a population-based study comparing three different definitions. Diabetic medicine : a journal of the British Diabetic Association. 2007;24(5):464-72.

40. Rana JS, Cote M, Despres JP, Sandhu MS, Talmud PJ, Ninio E, et al. Inflammatory biomarkers and the prediction of coronary events among people at intermediate risk: the EPIC-Norfolk prospective population study. Heart. 2009;95(20):1682-7.

41. Silverman MG, Blaha MJ, Krumholz HM, Budoff MJ, Blankstein R, Sibley CT, et al. Impact of coronary artery calcium on coronary heart disease events in individuals at the extremes of traditional risk factor burden: the Multi-Ethnic Study of Atherosclerosis. European heart journal. 2014;35(33):2232-41.

42. Sivapalaratnam S, Boekholdt SM, Trip MD, Sandhu MS, Luben R, Kastelein JJ, et al. Family history of premature coronary heart disease and risk prediction in the EPIC-Norfolk prospective population study. Heart. 2010;96(24):1985-9.

43. Sondermeijer BM, Boekholdt SM, Rana JS, Kastelein JJ, Wareham NJ, Khaw KT. Clinical implications of JUPITER in a contemporary European population: the EPIC-Norfolk prospective population study. European heart journal. 2013;34(18):1350-7.

44. van der Steeg WA, Boekholdt SM, Stein EA, El-Harchaoui K, Stroes ES, Sandhu MS, et al. Role of the apolipoprotein B-apolipoprotein A-I ratio in cardiovascular risk assessment: a case-control analysis in EPIC-Norfolk. Annals of internal medicine. 2007;146(9):640-8.

45. Ye S, Shaffer JA, Kang MS, Harlapur M, Muntner P, Epel E, et al. Relation between leukocyte telomere length and incident coronary heart disease events (from the 1995 Canadian Nova Scotia Health Survey). Am J Cardiol. 2013;111(7):962-7.
