# Supplementary material for: Research inefficiencies in external validation studies of the Framingham Wilson coronary heart disease risk rule: A systematic review
Source: PLoS One. 2024 Sep 13;19(9):e0310321. doi: 10.1371/journal.pone.0310321 (PMC12140082; doi:10.1371/journal.pone.0310321)
Supplement: Attachment — Submitted filename: Response to Reviewers JWB.docx [file pone.0310321.s009.docx]

Centre for Evidence-Based Medicine and Department for Continuing Education

University of Oxford
Oxford, United Kingdom

10 February 2023

Fares Alahdab

Academic Editor

PLOS ONE

Dear Dr Alahdab,

Thank you very much for inviting us to revise our manuscript. We are pleased to submit the revised version of our manuscript (PONE-D-22-18030) titled “Research inefficiencies in external validation studies of the Framingham Wilson coronary heart disease risk rule: A systematic review.”

We would also like to thank the peer reviewers for evaluating our manuscript and providing constructive feedback. We confirm that our electronic database searches have been updated as of December 2022 and our revised manuscript meets the journal’s style requirements. We tried our best to address the reviewers’ comments by explaining our rationale better, describing methods clearer, and discussing the results considering contexts.

For each question raised by the reviewers, which is highlighted in bold italic characters, we provided our response followed by any revision(s) made to the manuscript in bullet point (line in marked up copy). We look forward to hearing back from you soon.

With best wishes,

Jong-Wook Ban, MD, MSc, DPhil

**Reviewer #1**

***The external validation of clinical prediction rules is a key issue in their importance moving forward. But the relative inconsistency of how to do such studies and what to do with them is holding the field back. This study was a systematic review to assess how well that is going. There’s a lot of good work in here including a systematic review with good data extraction.***

1. ***The writing is generally good though they should follow standard structure at touch more. For example, the methods section is under-structured and I was unclear what the outcome measures were.***

We appreciate the reviewer’s comment. We restructured the methods section by adding a section titled “Outcome measures” and revised the contents of the data analysis section to help readers understand methods better.

- Line 230 - 236: “Research inefficiency occurs when research studies fail to “contribute to knowledge or to practice and policy,” to their maximum potential (22, 37). In our study, we assessed two types of inefficiency among external validation studies of the Framingham Wilson CHD risk rule. Firstly, we evaluated the inefficiency from external validation studies that do not attempt to update the Framingham Wilson CHD risk rule when it performs poorly; thus, missing the opportunity to create a CHD risk rule that could be useful for populations similar to the one used in the external validation.”
- Line 238 – 343: “Secondly, we assessed the inefficiency from external validation studies that are potentially eligible to be included in a meta-analysis and contribute to understanding the performance of the Framingham Wilson CHD risk rule, but fail to do so because either a P/O ratio or c statistic is not reported or cannot be obtained from provided data. We chose to evaluate the P/O ratio because existing systematic reviews (38-40) of the Framingham Wilson CHD risk rule invariably meta-analyzed the P/O ratio to summarize calibration.”
- Line 269 – 274: “We presented the proportion of external validation studies in which authors concluded that the Framingham Wilson CHD risk rule (a) performed adequately, (b) performed inadequately and updated the CPR, and (c) performed inadequately but did not update the CPR. We also calculated the proportion of eligible external validation studies that could not be included in the meta-analysis of P/O ratio or c statistic because a relevant performance measure was not reported or could not be estimated from provided data.”
- Line 276 – 282: “Additionally, we conducted post hoc citation analyses to evaluate the potential impact of external validation studies in which authors concluded that the Framingham Wilson CHD risk rule performed inadequately but did not update it. In Scopus, we ran forward citation searches of these external validation studies, which were updated in December 2022. We searched for studies that derived a new cardiovascular CPR while using the Framingham Wilson CHD risk rule's poor performance as one of the justifications and citing one of these external validation studies.”

2. ***Unfortunately, the two main criteria the study was looking at just didn’t strike me as fair or valuable. They assessed if (1) the new model updated the original and (2) if they assessed overall performance.***

***I’m unconvinced by their belief that any new model should make a new parameterization of the old. What do we do with a new parameterization of an old model? We have so many and applying them is unclear. We have dozens of new models based on old ones, the new ones aren’t externally valid either (and won’t be) because the differences between populations are real, not a sign of flaws that the next paper will correct. So I’m unclear what we’d do with the information and why.***

We revised the following paragraphs in the introduction section to more clearly explain why updating the sub-optimally performed CPR might be more efficient use of external validation studies.

- Line 64 - 75: “However, CPRs often perform unsatisfactorily when applied to populations different from the ones they are derived, in external validation studies (8-10), which might be due to inflated accuracy from suboptimal design and methods used to derive the CPRs or differences in populations and settings between derivation and external validation studies (4, 11).”
- Line 77 - 85: “Sometimes, authors of external validation studies declare that the CPRs, when they perform poorly, are not generalizable (12-14). There are several disadvantages for this approach. When no valid CPR exists for the population, clinicians cannot make decisions guided by a CPR until a new accurate CPR is created by often repeating the entire derivation process from ground up, because using poorly performing CPR can lead to decisions harmful to patients (15). Moreover, this means that the knowledge accumulated while deriving the CPR is simply wasted (11, 16). Further, this approach might promote the proliferation of many redundant CPRs for the same problem (11, 16) because the poor performance of CPR is one of the most commonly stated justifications by authors who derive a new cardiovascular CPR (17).”
- Line 87 - 97: “Alternatively, authors could attempt to update the CPR while conducting the external validation study when it performs poorly (3, 11, 16, 18). The performance of the CPR could improve simply by adjusting the calibration intercept or by re-estimating coefficients of predictor variables (11, 19-21). To the contrary to dismissing the CPR that performs poorly in external validation, previous research and evidence accrued is efficiently used when the CPR is updated (11, 16, 22). Although the updated CPR still should be externally validated, with this approach, it might be unnecessary for researchers to create a new CPR just for this population by rerunning the entire derivation process (11, 16). For example, the REGICO function was created by calibrating the Framingham Wilcon CHD risk rule in Spanish cohort (23), which has been successfully validated in various Spanish cohorts (24-26), and adopted by Spanish clinical practice guidelines (27, 28).

3. ***Their finding that model assessment is unclear and inconsistent is more valuable, though also problematic. Finding that CPRs don’t present C-statistics is bad and interesting. It fits my experience as well. But the calibration assessment, which the authors focus on, is much less clear. The authors emphasize the predicted to observed ratio. While I like this ratio also, it’s an atypical measure that isn’t even mentioned in the TRIPOD elaboration paper. Expecting it to be presented isn’t fair to current articles. To me the larger problem here is that it's legitimately unclear what calibration measures should be presented, not that papers are doing a poor job presenting those results.***

Calibration of a clinical prediction rule can be presented in several ways as shown in Table 2 of our manuscript. To our knowledge, existing systematic reviews of the Framingham Wilson CHD risk rule that meta-analyzed a calibration measure almost always focused on the P/O ratio. Therefore, we evaluated whether external validation studies of the Framingham Wilson CHD risk rule either reported a P/O ratio or provided sufficient data to calculate it; and could contribute to understanding the performance of the Framingham Wilson CHD risk rule, to their maximum potential. We revised the methods section to make this rationale clear to readers.

- Line 238 - 243: “Secondly, we assessed the inefficiency from external validation studies that are potentially eligible to be included in a meta-analysis and contribute to understanding the performance of the Framingham Wilson CHD risk rule, but fail to do so because either a P/O ratio or c statistic is not reported or cannot be obtained from provided data. We chose to evaluate the P/O ratio because existing systematic reviews (38-40) of the Framingham Wilson CHD risk rule invariably meta-analyzed the P/O ratio to summarize calibration.”

4. ***I suppose if it were me I would focus on measures of model assessment, de-emphasize the P:O ratio and simply show the # of papers that presented no measures of discrimination and no measure of calibration. I think everyone would agree that without those, an article is missing key features.***

We revised the results section to highlight the number and proportion of external validation studies that failed to report any discrimination and calibration measures.

- Line 316 - 486: “Performance measures of the Framingham Wilson CHD risk rule reported by the included studies are summarised in Table 2. Of 39 studies that aimed to externally validate the Framingham Wilson CHD risk rule, 17 (43.6%) did not reported any calibration measure. Only 14 (35.9%) studies reported calibration according to the recommendations from the TRIPOD statement: 5 (12.8%) studies with both calibration table and plot, 8 (20.5%) studies with a calibration plot only, one (2.6%) study with a calibration table only. A measure of discrimination was not reported in a minority (11 of 39, 28.2%) of studies that aimed to externally validate the Framingham Wilson CHD risk rule. However, c statistic was reported with a 95% confidence interval only in 16 (41.0%) external validation studies, adhering to the recommendation in the TRIPOD statement.”

**Reviewer #2**:

*The manuscript is well written and well structured. It focuses on an important area of research relevant to the quality of clinical prediction models. More specifically, it focuses on research inefficiencies in external validation studies of the Framingham Wilson coronary heart disease risk rule. Findings from this paper could be generalized to external validation studies in other domains.*

*The manuscript could benefit from:*

*- better consistency of study findings to align with objectives*

*- elaborating why only two areas of research inefficiencies were focused on versus including other TRIPOD items*

*- updating the search to include more recent publications*

*- realigning/restructuring presentation of results*

*More detailed comments are stated below.*

**ABSTRACT**

1. ***The authors indicate that 39 external validation studies were identified. Later in the manuscript the authors present that 97 studies were included. These additional studies should also be presented in the abstract to avoid confusion (they are also likely external validation studies).***

We appreciate the reviewer’s comment. We revised the abstract to avoid confusion by adding a sentence to present 98 included studies that evaluated the Framingham Wilson coronary heart disease (CHD) risk rule. Please note that we wish to keep the distinction between two types of include studies. We explained our rationale for this in our response to the reviewer’s comment 10.

- Line 34 - 35: “We identified 98 studies that evaluated the Framingham Wilson CHD risk rule; 40 of which were external validation studies.”

**INTRODUCTION**

2. ***The introduction is clear but would be helpful to readers to know when the Framingham Wilson CHD paper was published. This will help the reader to make a connection with the TRIPOD adherence depending on which year they were published - especially useful for the discussion section.***

We revised the introduction section and explained when the Framingham Wilson CHD risk rule was published.

- Line 147 - 149: “… a systematic review found that the Framingham Wilson coronary heart disease (CHD) risk rule, which was published in 1998, was the most frequently validated cardiovascular CPR with 89 external validation studies …”

**METHODS**

3. ***In the methods you mentioned "We evaluated a CPR for coronary heart disease (CHD) risk derived by Wilson et al. (24) using the Framingham Heart Study cohort in 1998." Perhaps you meant to be evaluating external validation studies of that CPR?***

We changed the paragraph to make it clear that we evaluated external validation studies of the Framingham Wilson CHD risk rule.

- Line 160 - 162: “We evaluated external validation studies of a CPR for coronary heart disease (CHD) risk derived by Wilson et al. (24) using the Framingham Heart Study cohort. Published in 1998, the Framingham Wilson CHD risk rule estimates the 10-year risk of CHD …”

4. ***Search was updated in June 2020. That was more than two years ago so there are likely other (and possibly improved) external validations of the Framingham Wilson CPR? I suggest updating it.***

We updated the forward citation search as of December 2022. In addition, the searches for the additional analysis were also updated. We revised the abstract, methods, and results section. Also, figure and tables have been updated accordingly.

- Line 35 - 47: “Of these 40 studies, 27 (67.5%) concluded the Framingham Wilson CHD risk rule performed poorly but did not update it. Of 23 external validation studies conducted with data that could be included in meta-analyses, 13 (56.5%) could not fully contribute to the meta-analyses ...”
- Line 173 - 174: “This initial search was updated in June 2020 and December 2022.”
- Line 278 - 279: “In Scopus, we ran forward citation searches of these external validation studies, which were updated in December 2022.”
- Line 293 - 294: “A total of 98 studies that evaluated the Framingham Wilson CHD risk rule from 84 publications were included, Fig 1.”
- Line 300 - 302: “For 40 (40.8%) of 98 included studies, one of the aims was externally validating the Framingham Wilson CHD risk rule. For 58 (59.2%) studies …”
- Line 673 – 675: “Of 40 external validation studies of the Framingham Wilson CHD risk rule, authors of 27 external validation studies (67.5%) concluded that the performance was inadequate but did not update the CPR.”
- Line 701 – 704: “In a post hoc citation analysis, a total of 1,341 references were found in forward citation searches of 26 external validation studies that concluded the performance of the Framingham Wilson CHD risk rule was inadequate but did not update it. From these references, we found 20 studies …”
- Line 704: “A total of 44 studies were included in the meta-analysis of the P/O ratio: 15 studies …”
- Line 738 - 740: “A total of 24 studies were included in the meta-analysis of *c* statistic: 16 studies with an explicit aim of externally validating the Framingham Wilson CHD risk rule and 8 studies where a *c* statistic could be obtained from data.”
- Line 765 – 767: “Of 40 studies that aimed to externally validate the Framingham Wilson CHD risk rule, 23 studies were conducted in the USA and Europe: therefore could have been included in the meta-analyses as presented in Table 3.”
- Line 808 – 810: “In summary, of 23 studies that aimed to externally validate the Framingham Wilson CHD risk rule, only ten (43.5%) contributed to understanding both the summary P/O ratio and c statistic in eligible meta-analyses. On the other hand, 13 (56.5%) studies that …”

5. **Scopus was used. Embase is known to include additional citations. Might be helpful to check with an information librarian if it is worth including other databases.**

We used the forward citation search because we have shown in the past that this method can efficiently and effectively identify external validation and impact studies of a clinical prediction rule (1, 2). We did not use traditional electronic databases such as EMBASE because they do not offer the citation search function.

There are three main search engines for citation analyses: Google Scholar, Scopus, and Web of Science. Although Google Scholar has consistently shown that it produces the most comprehensive results, we did not use it because it was impractical when handling a large number of references and the comprehensiveness of results often add little value due to irrelevant results (3). We chose Scopus because it tends to perform better than Web of Science in the health and medical sciences field (4).

6. ***In methods you indicate the search was not limited to any language. Did you find any in other languages? Did you end up needing a translator for them?***

Among 98 included studies (84 articles) that evaluated the Framingham Wilson CHD risk rule, five were only available in Spanish. We used Google Translate to translate these papers. Also, one of our co-authors was a native Spanish speaker and was available for help when needed.

7. ***Under Data Analysis, you indicate "(c) performed inadequately but did not update the CPR." I assume these are the studies that are considered inefficient. Could it be that they just wanted to externally validate the study and not update or derive a new rule? Or are these studies that ended up deriving a new rule? Please refer to TRIPOD and other studies which categorize external validation studies as with and without updating.***

The TRIPOD statement describes different cases of external validation studies, which can be with derivation and with/without updating. It recommends authors what to include in their reports. However, since the TRIPOD statement is a reporting guideline rather than methodological standard, it does not make recommendations about whether to update a CPR in external validation studies.

We revised the following paragraphs in the introduction and methods section to more clearly explain why external validation studies that do not attempt to update a CPR when it performs poorly might be inefficient research that do not contribute to knowledge and practice to their maximum potential. We also provided references from CPR literature recommending this approach.

- Line 77 - 85: “Sometimes, authors of external validation studies declare that the CPRs, when they perform poorly, are not generalizable (12-14). There are several disadvantages for this approach. When no valid CPR exists for the population, clinicians cannot make decisions guided by a CPR until a new accurate CPR is created by often repeating the entire derivation process from ground up, because using poorly performing CPR can lead to decisions harmful to patients (15). Moreover, this means that the knowledge accumulated while deriving the CPR is simply wasted (11, 16). Further, this approach might promote the proliferation of many redundant CPRs for the same problem (11, 16) because the poor performance of CPR is one of the most commonly stated justifications by authors who derive a new cardiovascular CPR (17).”
- Line 87 - 97: “Alternatively, authors could attempt to update the CPR while conducting the external validation study when it performs poorly (3, 11, 16, 18). The performance of the CPR could improve simply by adjusting the calibration intercept or by re-estimating coefficients of predictor variables (11, 19-21). To the contrary to dismissing the CPR that performs poorly in external validation, previous research and evidence accrued is efficiently used when the CPR is updated (11, 16, 22). Although the updated CPR still should be externally validated, with this approach, it might be unnecessary for researchers to create a new CPR just for this population by rerunning the entire derivation process (11, 16). For example, the REGICO function was created by calibrating the Framingham Wilcon CHD risk rule in Spanish cohort (23), which has been successfully validated in various Spanish cohorts (24-26), and adopted by Spanish clinical practice guidelines (27, 28).
- Line 229 - 236: “Outcome measures: Research inefficiency occurs when research studies fail to “contribute to knowledge or to practice and policy,” to their maximum potential (26, 27). In our study, we assessed two types of inefficiency among external validation studies of the Framingham Wilson CHD risk rule. Firstly, we evaluated the inefficiency from external validation studies that do not attempt to update the Framingham Wilson CHD risk rule when it performs poorly; therefore, misses the opportunity to create a CHD risk rule that could be useful for populations similar to the one used in the external validation.”

8. ***Authors could look into using the Critical Appraisal and Data Extraction for Systematic Reviews of Prediction Modelling Studies (CHARMS) to extract and present more comprehensive issues with external validation studies. For example, authors have referenced the TRIPOD guideline. There are several other items that could be included in this study including TRIPOD items 4-12.***

Many systematic reviews have already shown a wide range of methodological and reporting issues among external validation studies (5-9). Therefore, our study focused on two specific types of inefficiency among external validation studies of the Framingham Wilson CHD risk rule: external validation studies that do not attempt to update the Framingham Wilson CHD risk rule when it performs poorly and external validation studies that cannot contribute to understanding the performance of the Framingham Wilson CHD risk rule because either a P/O ratio or c statistic is not reported or cannot be obtained from provided data.

Although it is true that we might find shortcomings in methods and reporting, similar to the ones already demonstrated in existing systematic reviews, we believe that these are outside of this study’s scope and hope to focus on our primary objectives.

**RESULTS**

9. ***In the Introduction you state that "For example, a systematic review found that the Framingham Wilson coronary heart disease (CHD) risk rule was the most frequently validated cardiovascular CPR with 89 external validation studies (22)." Please see above but you report that you have identified only 39 external validation studies.***

We agree that the categories of included studies could have been explained better. Both our study and the systematic review by Damen et al. (reference 22) included two types of studies: (1) studies that aimed to externally validate the CPR, “external validation studies”, and (2) studies where a performance measure of the CPR could be obtained although they did not explicitly aim to externally validate the CPR. The first type of studies was the main interest of our study and denominator in the primary analyses. One such example is the study by Empana et al. (10) that assessed whether the Framingham Wilson coronary heart disease (CHD) risk rule could be applied to European populations.

The second type of studies where a performance measure of the CPR could be obtained was also included because they could contribute to meta-analyses. One example is the study by Buitrago et al. (11), which had a primary aim of assessing the accuracy of Cockcroft-Gault and simplified MDRD equations in patients with renal failure but also presented risk of CHD based on the Framingham Wilson CHR risk rule and actual coronary events. We believe, in the systematic review by Damen et al., all included studies were referred as “external validation.”

To make this distinction clear, we revised the following paragraph in the data collection section.

- Line 205 - 209: “The included studies were categorised into two groups: (1) studies in which one of the stated aims was externally validating Framingham Wilson CHD risk rule, which will be referred to as “external validation studies” in our study, and (2) studies that did not aim to externally validate the Framingham Wilson CHD risk rule but in which a valid performance measure of a CPR was reported or could be calculated using provided data.”

10. ***In connection with above point, I think there are more external validation studies than 39. It might be due to how you define external validation studies versus the other 58 studies. The other studies are also likely external validation studies but with reporting pitfalls (i.e. they are possibly not reporting that they are externally validating). Reporting pitfalls are common with derivation and validation studies and hence TRIPOD was developed as a guidance.***

As explained above, this is correct. However, we would like to keep the distinction between these categories of included studies because it might be unfair to expect for the second type of the include studies, which did not aim to externally validate the Framingham Wilson CHD risk rule but happened to contain data for meta-analyses, to follow recommendations from reporting guideline only relevant to CPR studies and to update the Framingham Wilson CHD risk rule when it performed poorly.

11. ***I understand that 26 out of 29 studies concluded the Framingham Wilson CHD risk rule performed poorly but did not update it. Some studies have no plan to either create a new rule nor update it but rather just want to see how well the rule performs on their patients, as is - these should be their own group. However, among the studies that either derived a new rule or updated the existing one, what proportions were deriving a new rule (vs updating it)?***

We analysed 40 studies that had a primary aim was externally validating the Framingham Wilson CHD risk rule, “external validation studies.” Of these, 27 external validation studies concluded that the performance was inadequate but did not update the CPR, as presented in Fig 2.

In addition to externally validating the Framingham Wilson CHD risk rule, four external validation studies (10.3%) also aimed to derive a new cardiovascular CPR. Two of these studies updated the Framingham Wilson CHD risk rule due to poor performance.

In addition to externally validating the Framingham Wilson CHD risk rule, four external validation studies (10.3%) aimed to update the Framingham Wilson CHD risk rule. These studies presented the updated Framingham Wilson CHR risk rule.

One study (2.6%) aimed to update the SCORE chart and updated it, in addition to externally validating the Framingham Wilson CHD risk rule.

One study (2.6%) did not explicitly aim to derive a new CPR but included a multivariable analysis, which could be considered as a new CPR.

12. ***In addition to above point, for the studies that updated the rule, how many considered less extensive methods prior to trying more extensive methods to update the prediction rule?***

Unfortunately, study authors did not provide details about how they updated the CPR such as attempting to use simple methods before applying more complex techniques. There were four external validation studies that updated the Framingham Wilson CHD risk rule. Two studies conducted a simple recalibration (12, 13). Two studies have conducted more comprehensive updates by re-estimating parameters and modifying predictor variables (14, 15). We incorrectly reported that the “three studies updated the CPR by recalibrating and one study by both recalibrating and re-estimating.” Therefore, we corrected the statement and provided references.

- Line 676 - 679: “… concluded the Framingham Wilson CHD risk rule performed poorly updated the CPR: two studies by recalibrating (43, 44) and two studies by re-estimating parameters and modifying predictor variables (45, 46).”

13. ***The results in the Tables would need to be reorganized once the studies are combined. Perhaps to list all as external validation studies but show a column that indicates if the authors reported their objectives clearly. Also, put more emphasis on results answering the objectives.***

This is related to the reviewer’s comment 10. Similarly, we would like to keep the distinction between these categories of included studies and focus on “external validation studies” for our main analysis because research inefficiency in these studies was what we aimed to examine. We updated the following paragraphs to highlight answers to our research questions.

- Line 673 - 675: “Of 40 external validation studies of the Framingham Wilson CHD risk rule, authors of 27 external validation studies (67.5%) concluded that the performance was inadequate but did not update the CPR.”
- Line 808 – 812: “In summary, of 23 studies that aimed to externally validate the Framingham Wilson CHD risk rule, only ten (43.5%) contributed to understanding both the summary P/O ratio and c statistic in eligible meta-analyses. On the other hand, 13 (56.5%) studies that aimed to externally validate the Framingham Wilson CHD risk rule did not contribute to eligible meta-analyses to their full potential …”

**DISCUSSION**

14. ***Findings from this study on deficiencies of external validation studies is a common issue in other domains. Would be useful to readers to compare with other systematic reviews in other domains.***

We revised the following paragraph in the comparison with existing literature section to highlight some of the key deficiencies of external validation studies documented in existing literature.

- Line 858 - 863: “Many systematic reviews have pointed out various methodological and reporting issues in external validation studies. For example, systematic reviews have shown that many external validation studies include an insufficient number of participants with and without outcome events (8, 69) which can result in biased and imprecise estimation of predictive performance measures (70, 71). Despite this, external validation studies infrequently describe how the sample size is justified (72, 73) and complete case analyses are common (8, 69).”
- Line 865 – 872: “These findings are consistent with systematic reviews in diabetes, cancer, chronic kidney disease, and cardiovascular disease that showed external validation studies often do not report recommended performance measures, especially for calibration (8, 30, 31, 74, 75). For example, Collins et al. (74) systematically reviewed studies externally validating CPRs from various clinical domains found that the majority (67.9%) of external validation studies did not report any calibration measure while a measure of discrimination was reported in most studies (73.1%).”

15. ***The authors indicate "Even when authors of the external validation studies reported these performance measures of the Framingham Wilson CHD risk rule, most did not adhere to the recommendations from the TRIPOD statement." As with the Introduction it would be good to know how many of the external validation studies were published prior to the TRIPOD statement -- a limitations to adhering to TRIPOD statement.***

We appreciate the reviewer’s comment. This is an important limitation of our study. We added a sentence discussing this in the strengths and limitations section.

- Line 900 - 907: “We assessed whether external validation studies of the Framingham Wilson CHD risk rule reported its performance measure following the recommendations from the TRIPOD statement, which was published in 2015. Because most external validation studies, except for three, that we evaluated predate the publication of the TRIPOD statement, our findings should not be interpreted as an assessment of reporting quality against the recommendations form the TRIPOD statement. Our intention was to investigate whether external validation studies provide sufficient information so that their results could contribute to the understanding the overall performance of the Framingham Wilson CHD risk rule.”

Things to complete

Update revised sentences.

Update line numbers

Additional documents needed

**References**

1.	Ban JW, Stevens R, Perera R. Predictors for independent external validation of cardiovascular risk clinical prediction rules: Cox proportional hazards regression analyses. Diagn Progn Res. 2018;2:3.

2.	Ban JW, Chan MS, Muthee TB, Paez A, Stevens R, Perera R. Design, methods, and reporting of impact studies of cardiovascular clinical prediction rules are suboptimal: a systematic review. J Clin Epidemiol. 2021;133:111-20.

3.	Chapman K, Ellinger AE. An evaluation of Web of Science, Scopus and Google Scholar citations in operations management. The international journal of logistics management. 2019;30(4):1039-53.

4.	Martin-Martin A, Thelwall M, Orduna-Malea E, Delgado Lopez-Cozar E. Google Scholar, Microsoft Academic, Scopus, Dimensions, Web of Science, and OpenCitations' COCI: a multidisciplinary comparison of coverage via citations. Scientometrics. 2021;126(1):871-906.

5.	Altman DG. Prognostic models: a methodological framework and review of models for breast cancer. Cancer Invest. 2009;27(3):235-43.

6.	Bouwmeester W, Zuithoff NP, Mallett S, Geerlings MI, Vergouwe Y, Steyerberg EW, et al. Reporting and methods in clinical prediction research: a systematic review. PLoS medicine. 2012;9(5):1-12.

7.	Collins GS, de Groot JA, Dutton S, Omar O, Shanyinde M, Tajar A, et al. External validation of multivariable prediction models: a systematic review of methodological conduct and reporting. BMC medical research methodology. 2014;14:40.

8.	Heus P, Damen J, Pajouheshnia R, Scholten R, Reitsma JB, Collins GS, et al. Poor reporting of multivariable prediction model studies: towards a targeted implementation strategy of the TRIPOD statement. BMC Med. 2018;16(1):120.

9.	Tan J, Qi Y, Liu C, Xiong Y, He Q, Zhang G, et al. The use of rigorous methods was strongly warranted among prognostic prediction models for obstetric care. Journal of clinical epidemiology. 2019;115:98-105.

10.	Empana JP, Ducimetiere P, Arveiler D, Ferrieres J, Evans A, Ruidavets JB, et al. Are the Framingham and PROCAM coronary heart disease risk functions applicable to different European populations? The PRIME Study. Eur Heart J. 2003;24(21):1903-11.

11.	Buitrago F, Calvo JI, Gomez-Jimenez C, Canon L, Robles NR, Angulo E. [Comparison and agreement of the Cockcroft-Gault and MDRD equations to estimate glomerular filtration rate in diagnosis of occult chronic kidney disease]. Nefrologia. 2008;28(3):301-10.

12.	Ferrario M, Chiodini P, Chambless LE, Cesana G, Vanuzzo D, Panico S, et al. Prediction of coronary events in a low incidence population. Assessing accuracy of the CUORE Cohort Study prediction equation. Int J Epidemiol. 2005;34(2):413-21.

13.	Reissigova J, Zvarova J. The Framingham risk function underestimated absolute coronary heart disease risk in Czech men. Methods Inf Med. 2007;46(1):43-9.

14.	Rodondi N, Locatelli I, Aujesky D, Butler J, Vittinghoff E, Simonsick E, et al. Framingham risk score and alternatives for prediction of coronary heart disease in older adults. PLoS One. 2012;7(3):e34287.

15.	Nishimura K, Okamura T, Watanabe M, Nakai M, Takegami M, Higashiyama A, et al. Predicting coronary heart disease using risk factor categories for a Japanese urban population, and comparison with the framingham risk score: the suita study. J Atheroscler Thromb. 2014;21(8):784-98.
