# Supplementary material for: Research inefficiencies in external validation studies of the Framingham Wilson coronary heart disease risk rule: A systematic review
Source: PLoS One. 2024 Sep 13;19(9):e0310321. doi: 10.1371/journal.pone.0310321 (PMC12140082; doi:10.1371/journal.pone.0310321)
Supplement: Attachment — Submitted filename: Response to Reviewers.docx [file pone.0310321.s010.docx]

Centre for Evidence-Based Medicine and Department for Continuing Education

University of Oxford
Oxford, United Kingdom

28 June 2023

Miquel Vall-llosera Camps

Senior Editor

PLOS ONE

Dear Dr Miquel Vall-llosera Camps,

Thank you very much for inviting us to revise our manuscript again. We are pleased to submit the revised version of our manuscript (PONE-D-22-18030) titled “Research inefficiencies in external validation studies of the Framingham Wilson coronary heart disease risk rule: A systematic review.”

We would like to thank the peer reviewers for evaluating our manuscript and providing constructive feedback. For each question raised by the reviewers, which is highlighted in bold italic characters, we provided our response followed by any revision(s) made to the manuscript in bullet point (line in marked up copy). We look forward to hearing back from you soon.

With best wishes,

Jong-Wook Ban, MD, MSc, DPhil

***Reviewer #1: I appreciate the response to my comments. I do still find the methods section confusing, primarily due to a tendency to not follow their outline.***

1. ***The “outcomes” section does not state the primary outcome. Research inefficiency is a nice theoretical construct, but it’s not an outcome measure. I believe it’s currently in data analysis (I think it’s P/O and c-stat), but I’m still not positive and it’s in the wrong place. “Our primary outcome is the reporting of P/O ratio and c-statistic in the valiation study.” Or something.***

We added the following sentences to the outcome measures sections to define our main outcome measures clearly.

- Line 257-259: “Therefore, one of our principal outcome measures was the proportion of external validation studies that concluded the Framingham Wilson CHD risk rule performed inadequately but did not update the CPR.”

- Line 264-267: “Thus, our other main outcome measure was the proportion of eligible external validation studies that could not be included in the meta-analysis of P/O ratio or c statistic due to the insufficient reporting of relevant performance measures.”

2. ***The first sentence of data analysis is actually about inclusion criteria, right? Shouldn’t that be in study selection checklist?***

We apologize for the confusion. The first sentence is not about inclusion criteria, but about which studies are used in the meta-analysis. To make the method clearer to readers, we revised the section. Firstly, we explained how we analyzed the inefficiency from external validation studies that do not update the Framingham Wilson CHD risk rule when it performs poorly. Then, we described how we measured the inefficiency from external validation studies that are potentially eligible to be included in a meta-analysis but fail to do so due to the insufficient reporting of relevant performance measures.

- Line 272-275: “Using all included external validation studies, we calculated the proportion of studies in which authors concluded that the Framingham Wilson CHD risk rule (a) performed adequately, (b) performed inadequately and updated the CPR, and (c) performed inadequately but did not update the CPR.”

- Line 277-280: For studies conducted in the USA and European geographic regions with comparable cardiovascular disease burden, the prevalence of risk factors, and health care environment (41): the UK, Northern Europe, Western Europe, and Southern Europe, we meta-analysed the Predicted/Observed (P/O) event ratio and c statistic of the Framingham Wilson CHD risk rule.

- Line 286-288: We presented the proportion of eligible external validation studies that could not be included in the meta-analysis of P/O ratio or c statistic because a relevant performance measure was not reported or could not be estimated from provided data.

3. ***The first paragraph of Information Sources is a description of the Wilson score, which is not an information source and should probably be in the introduction.***

We moved the paragraph to the introduction section and revised it to fit the flow.

- Line 149-158: “In 1998, Wilson et al. (33) derived a CPR for coronary heart disease (CHD) risk using the Framingham Heart Study cohort. The Framingham Wilson CHD risk rule estimates the 10-year risk of CHD (composite of angina pectoris, acute myocardial infarction, coronary insufficiency, and CHD death) and hard CHD (all CHD outcomes except for angina pectoris) with the following predictors: age, cigarette use, diabetes mellitus status, total and high-density lipoprotein cholesterol categories, and blood pressure categories (33). Systematic reviews (31, 34) have shown that more external validation studies for the Framingham Wilson CHD risk rule have been conducted than any other cardiovascular CPR. For example, a systematic review found that the Framingham Wilson CHD risk rule was the most frequently validated cardiovascular CPR with 89 external validation studies (31).”

**Reviewer #3**: The R1 revision of the manuscript and the responses have addressed the previous reviewers' comments. While I agree with a previous reviewer that the meta-analyses of P/O ratio as an assessment of calibration is less clear compared with meta-analyses of C-statistics as an assessment of discrimination, there is probably little researchers can improve based on aggregated data in the framework of systematic review + meta-analyses (unless individual level data could be used).

I have two suggestions stated below.

4. ***Lines 283-308 and texts that corresponds to Fig 3-A, 3-B and Fig 4: Although under "Study Selection" the authors stated that the cardiovascular outcome evaluated was "either CHD (a composite of angina pectoris, acute myocardial infarction, coronary insufficiency, and CHD death) or hard CHD (a composite of acute myocardial infarction, coronary insufficiency, and CHD death)", the authors did not mention what exact definition of the outcome has been used for each study (for example, in the form of stratified or sub analyses) in the meta-analyses of P/O ratio (Fig 3-A, 3-B) and C-statistics (Fig 4). The definition of reported cardiovascular outcome differs from study to study, for example, "death from CHD" is quite different from "total or hard CHD". I suggest that another supplemental material could list the definition of cardiovascular outcome(s) by study that correspond to extracted data in this review and the meta-analyses of P/O ratio and C-statistics.***

As suggested by the reviewer, we provided the outcome definitions from studies that were included in the meta-analyses in a supplemental table.

- Line 281-282: “The definitions of CHD from external validation studies included in the meta-analyses are summarized in S1 Table.”

5. ***Figures 3-A and 3-B: It would be better if two columns of numbers, "predicted n/N" and "observed n/N" that have been extracted from the studies, can be displayed on the forest plots, same as references 38 (Brindle et al) and 39 (Eichler et al). The numerators must correspond to the specific definition of reported cardiovascular outcome in the supplemental material (mentioned above).***

We revised Figure 3-A and 3-B to add the numbers of predicted and observed events.

**Reviewer #4**: I feel this is a very well written article. It illustrates some important shortcomings in current external validation studies. The use of the TRIPOD reporting guidelines and the value of reducing research inefficiencies and waste are important messages that are well illustrated by this article. I agree that by not following TRIPOD reporting guidelines, there are lost opportunities to contribute to further understanding of CPRs. As noted, when a CPR performs well, clinicians more likely to have confidence in it and use it – there is a need to avoid just dismissing it if doesn’t perform well. The authors have made an excellent choice in selecting the Framingham Wilson coronary heart disease (CHD) risk rule, given its longstanding, wide use in clinical practice.

6. ***I thought the method for identifying studies to be logical and appropriate. I would suggest the authors justify their use of Scopus over other forward-citation tools, as the results differ by source. An initial check of three sources showed that Scopus does produce the largest number of results (7442), followed by Web of Science (6717) and PubMed (2666). Although there will be significant overlap across these sources, I believe there will still be unique results from each, so it is possible some potentially relevant records were missed due to a full reliance on Scopus.***

As advised, we further explained the reasons for choosing Scopus in the information source and search for external validation studies section. Also, we acknowledge the possibility that relevant records were missed because we used a single data source in the strength and limitation section.

- Line 174-178: “We conducted our forward citation search using Scopus as a citation index because we have previously demonstrated that this method could efficiently and systematically identify CPR studies (35, 36). Further, for citation searches in the health science field, Scopus tends to produce more robust results than Web of Science while minimally missing unique references (37, 38).”

- Line 968-972: “On the other hand, we only relied on Scopus to conduct our forward citation search. Although the use of a single citation index is a very common practice (81), and Scopus performs well compared with Web of Science in the health science field (37, 38), searching multiple sources might have been more ideal to ensure none of the potentially relevant references was missed.”

I also found the inclusion criteria logical and would commend the authors for considering the availability of performance measures in the absence of a completed external validation. I thought the post hoc analyses of group C in which it was noted the Framingham RR performed inadequately but the authors did not update CPR, particularly interesting.

7. ***I would like to know why only studies in USA and Europe were considered and believe this needs more explanation in the text. I would think Australia, New Zealand and Canada would also have a comparable CVD burden.***

Interpreting random-effects meta-analyses that combine the results of external validation studies from multiple countries, over different time periods, with different eligibility criteria can be difficult. Ideally, a meta-analysis of external validation studies should include studies from “plausibly related populations.” (1) However, it is not always easy to clearly determine the plausibility. Rather, we often need to evaluate how plausible it is that populations are related, which requires an epidemiological understanding about the topic but also subjective judgement. In our study, we took a conservative approach and only meta-analyzed the studies from Europe and grouped them into studies form the UK, Northern Europe, Western Europe, and Southern Europe. Nevertheless, there were only one study from Australia and one study from Canada; including these two studies would not have changed the overall conclusion of our study.

I am unable to comment on the statistical methods or results. However, the overall conclusions were sound and quite compelling. Why dismiss existing tools and replace them rather than improve and update an existing tool and contribute to the associated evidence base? I like the reference to the power of journals in encouraging authors to justify their work and to undertake updates of a CPR when it performs poorly.

This is a very practical and useful article.

**Reviewer #5**: Overall, the paper addresses an important topic by investigating the research inefficiencies in studies evaluating the Framingham Wilson coronary heart disease (CHD) risk rule. The authors systematically review the literature, including 98 studies, and focus on two types of research inefficiencies: failure to update the CHD risk rule and inability to contribute to understanding the overall performance of the rule.

The paper provides valuable insights into the extent of inefficiencies in the research process, highlighting that many studies did not update the risk rule despite finding its performance inadequate. Additionally, a significant number of studies did not contribute to eligible meta-analyses due to the lack of reporting relevant performance measures or providing data for their estimation. The paper is well-structured and presents the results in a clear and organized manner, using tables and figures to help readers understand the findings. The meta-analyses conducted by the authors also add to the strength of the study, as they synthesize the data from multiple sources to provide a more comprehensive understanding of the risk rule's performance. In particular, the methodology used in this SR showed certain strengths:

• Systematic review: The authors conducted a systematic review of the literature, following the PRISMA guidelines. This approach helps ensure a comprehensive and unbiased selection of studies for inclusion, enhancing the validity of the paper's conclusions.
• Inclusion of many studies: The paper included a total of 98 studies, providing a broad and diverse sample to assess the research inefficiencies related to the Framingham Wilson CHD risk rule.
• Use of meta-analyses: The authors conducted meta-analyses to synthesize data from multiple studies, providing more precise estimates of the risk rule's performance across different populations and settings. This approach adds to the strength of the study, as it enables a more comprehensive understanding of the risk rule's overall performance.
• Assessment of study characteristics and reporting: The paper thoroughly evaluates the characteristics of the included studies, as well as their reporting of performance measures. This assessment helps identify areas where research practices could be improved to address the observed inefficiencies.

8. **However, there was Lack of exploration of reasons behind inefficiencies**: ***The paper does not delve deeply into the underlying reasons for the observed research inefficiencies. Understanding these factors could help inform more specific recommendations for improving research practices in this area. The authors could conduct further analyses or interviews with researchers to better understand the factors contributing to the observed research inefficiencies. This could help inform targeted recommendations for improving research practices.***

We appreciate and agree with the reviewer’s comment. In fact, for the derivation step, we published a study exploring one of the key inefficiencies and evaluated why researchers keep deriving new cardiovascular CPRs despite many similar CPRs already exist (2). However, exploring reasons why the research inefficiencies occur in external validation studies and identifying factors associated with these inefficiencies, which could be used to develop targeted interventions, are beyond the aim and scope of our study. Therefore, we revised the research and clinical implications section to discuss this.

- Line 999-1004: “In this study, we identified two key inefficiencies in the external validation sept of cardiovascular CPR development. Future studies (e.g. qualitative interviews or analysis of CPR registry) could further explore causes and mechanisms for these inefficiencies. Understanding factors that influence these inefficiencies to occur might help develop targeted ways to prevent them and increase the chance for external validation studies to fully contribute to knowledge and practice.”

The writing quality of the paper is generally good, with clear language and appropriate terminology. The organization of the paper follows a logical structure, with a clear introduction, methods, results, and discussion sections. The clinical usefulness of the paper is evident, as it informs researchers and clinicians about the potential limitations of the risk rule and the need for updating it.

In conclusion, the paper provides a valuable contribution to the literature by highlighting the research inefficiencies in studies evaluating the Framingham Wilson CHD risk rule. This could potentially lead to improvements in future research and help enhance the overall quality and utility of clinical prediction rules in the field of cardiovascular disease.

**References**

1.	Steyerberg EW. Clinical prediction models : a practical approach to development, validation, and updating. New York: Springer; 2009. xxviii, 497 p. p.

2.	Ban JW, Wallace E, Stevens R, Perera R. Why do authors derive new cardiovascular clinical prediction rules in the presence of existing rules? A mixed methods study. PloS one. 2017;12(6):e0179102.
