# Supplementary material for: Research inefficiencies in external validation studies of the Framingham Wilson coronary heart disease risk rule: A systematic review
Source: PLoS One. 2024 Sep 13;19(9):e0310321. doi: 10.1371/journal.pone.0310321 (PMC12140082; doi:10.1371/journal.pone.0310321)
Supplement: Attachment — Submitted filename: Response to Reviewers.docx [file pone.0310321.s011.docx]

Academic Editor’s Comments:

1. ***lines 141-144 and 216-217: How does your third study selection criterion match with your CHD definitions for meta-analysis (Table S1)?***

The Framingham Heart Study defined the coronary heart disease (CHD) outcome and its components using explicit and detailed criteria [1], which were then used when deriving the Framingham Wilson CHD risk rule [2]. It would have been simple if studies externally validating the Framingham Wilson CHD risk rule used the same definitions and criteria. However, no external validation study exactly followed the ones from the Framingham Heart Study.

We suspect that heterogeneous outcome definitions were used because, since the Framingham Heart Study defined these outcomes several decades ago, standard practices for evaluating patients for CHD have evolved greatly [3-5]. For example, the Framingham Heart Study's definition of acute myocardial infarction was based on serial changes in electrocardiogram findings and elevated serum markers such as glutamic oxaloacetic transaminase (GGT) and lactic dehydrogenase (LDH) [1]. However, criteria such as GGT and LDH have become obsolete in clinical practice and were replaced by better diagnostic tools such as troponin-I assay, exercise stress test, and scintigraphy, which were often used to determine CHD outcomes in external validation studies.

Further, we consider it is useful to study whether the Framingham Wilson CHD risk rule can accurately predict CHD in changing times, locations, and circumstances (please also refer to our response to comment #2 for more details). Therefore, we included studies that evaluated either CHD or hard CHD, as described in our third selection criteria. For example, a study by Herrera et al. [6] defined angina pectoris as "compatible chest pain history plus a positive exercise stress test, scintigraphy, or coronary arteriogram." Although different from the Framingham Heart Study's original definition, we determined that Herrera et al.'s outcome definition of angina pectoris [6] was sensible because it was consistent with modern standards for evaluating stable patients with chest pain [3-5]. We added the following sentence to the method section to explain to readers.

- Line 155-158: “When studies defined CHD outcomes using criteria different from the ones used by the Framingham Heart Study (40) due to evolving practice standards over time, we judged whether the modifications were relevant to current clinical practice.”

2. ***lines 216-217: Provide some solid arguments why a meta-analysis makes sense when the involved studies differ so much in their outcome definitions (Table S1).***

Internal validation assesses the reproducibility of a clinical prediction rule (CPR) in the same population where the CPR is derived [7, 8]. On the other hand, external validation studies evaluate how well the CPR performs when tested in populations different from but "plausibly related to" the derivation population [8, 9]. In practice, it means that, by conducting external validation studies, CPRs are applied to circumstances or settings of the CPRs' intended use that might differ regarding locations, time, methods, or disease spectrum [8, 10]. Therefore, heterogeneity is inherent and expected in external validation studies, which might include one that originates from differences in predictor and outcome definitions [10].

Similarly, meta-analysis results of external validation studies summarise the average performance of the CPR across various circumstances or settings and often contain heterogeneity due to the differences in predictor and outcome measurements [11, 12]. Therefore, having heterogeneity is not necessarily a contraindication for conducting meta-analyses of external validation studies but is rather an opportunity to explore potential sources of variation [12], as we did in our study for geographic locations. Although a similar subgroup analysis (or meta-regression) investigating the heterogeneity due to different outcome definitions would have been possible, this was not one of our aims. Furthermore, many external validation studies in our study were also included in the existing meta-analyses of the Framingham Wilson CHF risk rule [13, 14].

3. ***line 250, Table 1: Describe the difference between a "Risk equation" and a "Risk score".***

Wilson et al. [2] presented their CHD risk rule in two formats: (1) regression equations that estimate the 10-year risk of developing CHD using coefficients of predictor variables and (2) simplified scoring systems that categorise the 10-year risk of developing CHD based on the sum of points assigned to applicable predictor variables. We added the following texts in the legend of Table 1.

Line 271-272: “^a^ Risk equation: regression equations that estimate the 10-year risk of developing CHD using coefficients of predictor variables.”

“^b^ Risk score: simplified scoring systems that categorise the 10-year risk of developing CHD based on the sum of points assigned to applicable predictor variables.”

4. ***line 252: "Journal" instead of "Jounral"***

Apologies for the error. We corrected the misspelling in the legend of Table 1.

5. ***line 261: "16 of 40 (40 %)", in Table 2 we find "17 (42.5)"***

Apologies for the error. In the previous revision, when the contents of Table 2 were revised with an additional study found in the updated search, we missed reflecting it into the text. We revised the text and provided the correct data.

- Line 281-282: “… c statistic was reported with a 95% confidence interval only in 17 of 40 (42.5%) external validation studies, …”

6. ***line 270 and Fig. 2: "27 studies", in line 282 we find "26 studies". Is this a simple typo? Or did you omit one study in the post hoc citation analysis (which one and why)?***

Apologies for the error. When the texts were updated in the previous revision, we missed updating the figure. Therefore, the values in Fig. 2 have now been updated.

7. ***line 327: "Six studies", in Table 3 we can find 8 studies.***

Six external validation studies could not be included in the meta-analysis of the P/O ratio because they neither reported a P/O ratio nor provided sufficient information about the data to estimate it. These six studies (Mainous 2007 (51), Gander 2014 (54), Van Der Heijden 2009 (14), Merry 2012 (59), Guckelberger 2006 (62), and Protopsaltis 2004 (67)) are categorised in the table as “Not available*” in the fourth column.

8. ***line 339: "ten (43.5%)", in Table 3 we can only find 9 studies.***

These ten studies are Orford 2002 (50), Rodondi 2012 (48), Emapana 2003 (55), Simmons 2008 (56), Brunner 2010 (57), Empana 2003 (60), Becker 2008 (63), Marrugat 2007 (24), Cañón-Barroso 2007 (65), Calvo-Hueros (66). For Simmons 2008 (56), conducted with 10.295 participants from the EPIC-Norfolk cohort, the study was eligible for the meta-analysis of *c* statistic. However, it was ineligible for the meta-analysis of the P/O ratio because Sivapalaratnam (2010 Oct), using a much larger sample of 22,841 participants from the same cohort, was included. To avoid misunderstanding and make the intent clear, we revised the sentence.

- Line 363-365: “…of 23 studies that aimed to externally validate the Framingham Wilson CHD risk rule, only ten (43.5%) contributed to understanding its performance in eligible meta-analyses of P/O ratio and *c* statistic.”

9. ***line 340: "13 (56.5%)", in Table 3 we find 14 studies.***

Similar to the comment 8, we consider Simmons 2008 (56) fully contributed to its eligible meta-analysis because it was only eligible for the meta-analysis of *c* statistic. Therefore, we believe 13 of 23 (56.5%) did not contribute to eligible meta-analyses to their full potential.

10. ***lines 427-434: In the manuscript, you criticize the studies that they don't follow the TRIPOD. Now you explain that all except three studies predate TRIPOD as the statement appeared in 2015. This explanation should appear much earlier in the paper (e.g. in the Indroduction section).***

As recommended, we acknowledged in the introduction section that the TRIPOD statement was published recently and the authors of studies predating it did not have reporting guidance.

- Line 94-100: “According to the TRIPOD statement published in 2015, calibration and discrimination measures "should be reported in all prediction model papers" (29). However, systematic reviews have shown that external validation studies often do not report recommended performance measures, especially measures of calibration (8, 30, 31). Most studies included in these systematic reviews predate the TRIPOD statement, and authors might not have guidance for appropriate reporting. However, it is still true that when external validation studies do not report relevant performance measures …”

11. ***line 431: "from" instead of "form"?***

Apologies. We corrected the misspelling.

- Line 456-458: “… our findings should not be interpreted as an assessment of reporting quality against the recommendations from the TRIPOD statement.”

12. ***line 433: "understanding of" instead of "understandin"?***

Apologies. We corrected the misspelling.

- Line 459-460: “… so that their results could contribute to the understanding the overall performance of the Framingham Wilson CHD risk rule.”

13. ***line 444: "step" instead of "sept"?***

Apologies. We corrected the misspelling.

- Line 471-472: “…we identified two key inefficiencies in the external validation step of cardiovascular CPR development.”

14. ***lines 478-480: This sentence is hard to understand and should be reformulated.***

We revised the conclusion section to make the message clear.

- Line 502-514: “The focus of cardiovascular CPR research should shift from deriving many new CPRs to externally validating existing CPRs and updating them when needed because the potential end-users, such as patients, clinicians, researchers, and decision-makers, need to understand the generalizability of the CPRs. However, we have shown that conducting more external validation studies of a CPR does not always strengthen the CPR's evidence of generalizability. Further, we demonstrated that dismissing a CPR when it performs poorly can lead to the creation of new, potentially redundant CPRs. Therefore, in addition to shifting the focus of CPR research, the authors of external validation studies should ensure that they are using appropriate design and methods and providing sufficient information about the conducts and results so that the evidence they generate can help the end-users about the generalizability of the CPRs.”

15. ***lines 760-761: Supporting information S5 and S6 must be appropriately mentioned in the manuscript.***

We added the following sentences to the methods section to introduce supporting information: S5 and S6. Please note that supporting documents are re-ordered to match the order in which they appear in the main text.

- Line 247-249: “We prepared this report following all applicable recommendations from the Preferred Reporting Items for Systematic Reviews and Meta-Analyses (PRISMA) statement (48) as summarised in S3 Appendix. Data from our study are provided in S4 Dataset.”

16. ***Fig. 1: "measure" instead of "meausre"***

We corrected the misspelling in Fig. 1 to read “measure.”

17. ***Fig. 3-A: Shouldn't it read "Orford (...)" instead of "outcome"?***

We corrected the misspelling in Fig. 3-A to read “Orford (2002 Jul).”

18. ***Fig. 3-B: "Belgium" instead of "Belbium"***

We corrected the misspelling in Fig. 3-B to read “Belgium.”

19. ***Figures 3-A and 3-B: Using the Fowkes (2008) paper, I tried to confirm the 777 observed participants with "outcome" of the ARIC study by way of example, and I failed. How have the observed AND predicted participants been computed in the manuscript for the studies of Figures 3-A and 3-B? You could provide this information as supporting information S7.***

The Framingham Wilson CHD risk rule predicts a 10-year risk of CHD outcome. However, some studies assessed the CHD outcome with follow-up durations different from 10 years. For example, the ARIC cohort from Fowkes (2008) reported 571 CHD events with a median follow-up of 13.1 years for men and 362 CHD events with a median follow-up of 13.2 years for women. We added S2 Appendix to explain how these data were used to estimate CHD events anticipated with a 10-year follow-up.

20. ***Figures 3-A and 3-B: Which formula did you use for the confidence interval of the P/O ratio? You could provide this information as supporting information S7.***

We added S2 Appendix to explain how we conducted meta-analyses using methods described by Debray et al. [15].

21. ***Figures 3-A, 3-B and 4: According to lines 304 and 309, N is the total number of participants. However, when checking the Orford ("outcome") study which is the first study of both Figures 3-A and 4, there are 5,611 versus 1,393 participants, respectively. Is there a typo somewhere or is the former number the person-years-under-risk? A thorough check of all the reported participant numbers is necessary.***

Apologies for these errors. We have checked all the data, calculations, and results presentation in these figures and made corrections if needed.

Previous version of Fig 3-A

Revised Fig 3-A

Previous version of Fig 3-B

Revised Fig 3-B

Previous version of Fig 4

Revised Fig 4

22. ***Fig. 4: According to Table 3, there should be 16 "bold" studies, not just 9. It seems that Western and Southern Europe have been forgotten.***

We revised Fig. 4 by changing the characters of five relevant Western and Southern European studies to make the information consistent with Table 3. There are now 16 “bold” studies in Fig. 4.

23. ***Fig. 4, USA: Two studies with more than 14,000 participants have rather wide confidence intervals compared to studies with much smaller number of participants. Is there a sound formal explanation for this fact?***

Thank you for pointing these out. We reviewed all calculations related to the meta-analysis of *c* statistic in Fig. 4 and made appropriate changes.

For Dene 2007, the number of previously reported participants was incorrect. Although the study included a total of 14,749 participants, only 1,264 participants were used in the calculation. Therefore, we corrected the number of participants for Dene 2007.

On the other hand, for Mainous 2007, the reported number of participants presented was correct. However, an incorrect value was used in our meta-analysis by mistake. Now, the appropriate confidence interval for the *c* statistic is presented.

24. ***Fig. 4: How has the c statistic been computed? Which formula did you use for the confidence interval of the c statistic? You could provide this information as supporting information S7.***

We added S2 Appendix to explain how we conducted meta-analyses using methods described by Debray et al. [15].

***Reviewer #4: I am generally satisfied with the responses but feel the authors could still include more information as to why the regional areas (e.g., US and Europe) were selected. They have provided an excellent response to me, the reviewer, but this is not included in the paper, which would be helpful to the reader.***

As advised by the reviewer, we modified our response to the reviewer’s comment from the previous revision and included the following texts in the paper to help the readers understand why studies were meta-analysed by regional areas.

Line -: “Arguably, a meta-analysis of external validation studies should include studies conducted in populations that are “plausibly related” (45), which is not always easy to determine clearly. In our study, we took a conservative approach and included in meta-analyses only the studies conducted in the USA and European geographic regions with comparable cardiovascular disease burden, the prevalence of risk factors, and healthcare environment (46): the UK, Northern Europe, Western Europe, and Southern Europe. We meta-analysed the Predicted/Observed (P/O) event ratio …”

1.	Kannel WB, Wolf PA, Garrison RJ, Cupples LA, D'Agostino RB, National Heart L, et al. The Framingham study : an epidemiological investigation of cardiovascular disease / Section 34 : Some risk factors related to the annual incidence of cardiovascular disease and death using pooled repeated biennial measurements : Framingham heart study, 30 year followup / L. Adrienne Cupples and Ralph B. D'Agostino. [Bethesda, Md.]: National Heart, Lung and Blood Institute [Bethesda, Md.]; 1987.

2.	Wilson PW, D'Agostino RB, Levy D, Belanger AM, Silbershatz H, Kannel WB. Prediction of coronary heart disease using risk factor categories. Circulation. 1998;97(18):1837-47. PubMed PMID: 9603539.

3.	Fihn SD, Gardin JM, Abrams J, Berra K, Blankenship JC, Dallas AP, et al. 2012 ACCF/AHA/ACP/AATS/PCNA/SCAI/STS guideline for the diagnosis and management of patients with stable ischemic heart disease: executive summary: a report of the American College of Cardiology Foundation/American Heart Association task force on practice guidelines, and the American College of Physicians, American Association for Thoracic Surgery, Preventive Cardiovascular Nurses Association, Society for Cardiovascular Angiography and Interventions, and Society of Thoracic Surgeons. Circulation. 2012;126(25):3097-137. Epub 20121119. doi: 10.1161/CIR.0b013e3182776f83. PubMed PMID: 23166210.

4.	Task Force M, Montalescot G, Sechtem U, Achenbach S, Andreotti F, Arden C, et al. 2013 ESC guidelines on the management of stable coronary artery disease: the Task Force on the management of stable coronary artery disease of the European Society of Cardiology. European heart journal. 2013;34(38):2949-3003. Epub 20130830. doi: 10.1093/eurheartj/eht296. PubMed PMID: 23996286.

5.	Gulati M, Levy PD, Mukherjee D, Amsterdam E, Bhatt DL, Birtcher KK, et al. 2021 AHA/ACC/ASE/CHEST/SAEM/SCCT/SCMR Guideline for the Evaluation and Diagnosis of Chest Pain: Executive Summary: A Report of the American College of Cardiology/American Heart Association Joint Committee on Clinical Practice Guidelines. Circulation. 2021;144(22):e368-e454. Epub 20211028. doi: 10.1161/CIR.0000000000001030. PubMed PMID: 34709928.

6.	Herrera S, Guelar A, Sorli L, Vila J, Molas E, Grau M, et al. The Framingham function overestimates the risk of ischemic heart disease in HIV-infected patients from Barcelona. HIV Clin Trials. 2016;17(4):131-9. Epub 20160512. doi: 10.1080/15284336.2016.1177266. PubMed PMID: 27169692.

7.	Steyerberg EW. Validation of Prediction Models. In: Steyerberg EW, editor. Clinical Prediction Models: A Practical Approach to Development, Validation, and Updating. Cham: Springer International Publishing; 2019. p. 329-44.

8.	Justice AC, Covinsky KE, Berlin JA. Assessing the generalizability of prognostic information. Annals of internal medicine. 1999;130(6):515-24. PubMed PMID: 10075620.

9.	Altman DG, Royston P. What do we mean by validating a prognostic model? Statistics in medicine. 2000;19(4):453-73. PubMed PMID: 10694730.

10.	Van Calster B, Steyerberg EW, Wynants L, van Smeden M. There is no such thing as a validated prediction model. BMC medicine. 2023;21(1):70. doi: 10.1186/s12916-023-02779-w.

11.	Debray TP, Vergouwe Y, Koffijberg H, Nieboer D, Steyerberg EW, Moons KG. A new framework to enhance the interpretation of external validation studies of clinical prediction models. Journal of clinical epidemiology. 2014. doi: 10.1016/j.jclinepi.2014.06.018. PubMed PMID: 25179855.

12.	Debray TP, Damen JA, Snell KI, Ensor J, Hooft L, Reitsma JB, et al. A guide to systematic review and meta-analysis of prediction model performance. Bmj. 2017;356:i6460. Epub 20170105. doi: 10.1136/bmj.i6460. PubMed PMID: 28057641.

13.	Damen JA, Hooft L, Schuit E, Debray TP, Collins GS, Tzoulaki I, et al. Prediction models for cardiovascular disease risk in the general population: systematic review. Bmj. 2016;353:i2416. doi: 10.1136/bmj.i2416. PubMed PMID: 27184143; PubMed Central PMCID: PMCPMC4868251.

14.	Eichler K, Puhan MA, Steurer J, Bachmann LM. Prediction of first coronary events with the Framingham score: a systematic review. American heart journal. 2007;153(5):722-31, 31 e1-8. doi: 10.1016/j.ahj.2007.02.027. PubMed PMID: 17452145.

15.	Debray TP, Damen JA, Riley RD, Snell K, Reitsma JB, Hooft L, et al. A framework for meta-analysis of prediction model studies with binary and time-to-event outcomes. Statistical methods in medical research. 2019;28(9):2768-86. Epub 20180723. doi: 10.1177/0962280218785504. PubMed PMID: 30032705; PubMed Central PMCID: PMCPMC6728752.
