# Supplementary material for: Research inefficiencies in external validation studies of the Framingham Wilson coronary heart disease risk rule: A systematic review
Source: PLoS One. 2024 Sep 13;19(9):e0310321. doi: 10.1371/journal.pone.0310321 (PMC12140082; doi:10.1371/journal.pone.0310321)
Supplement: Attachment — Submitted filename: Response to Reviewers.docx [file pone.0310321.s012.docx]

1. ***Thank you for your revision. Unfortunately, you did not address a major issue properly. Looking at your Figures 3A, 3B and 4, there is an enormous heterogeneity of the study results. In particular, 3A (USA) and 3B (Western Europe) show extremely contradicting study results (“qualitative” heterogeneity). Therefore I asked: Provide some solid arguments why a meta-analysis makes sense when the involved studies differ so much in their outcome definitions? You provided an interesting answer for me but not for your audience (future readers of your paper). That is, you should address the question in the paper, thereby referencing to Figures 3A, 3B and 4, in particular 3A (USA) and 3B (Western Europe). Provided that this enormous heterogeneity is not due to different outcome definitions, some additional explanation is needed in any case.***

We accept that the summary statistics are of unclear value when heterogeneity is high. Since the summary statistics are largely incidental to our paper and not relevant to either our primary or secondary research question, we have removed them from the Forest plots.

We have therefore added the following text to the Methods section:

- Line 227-230: “At the suggestion of a reviewer, we display results in forest plots without summary estimates when heterogeneity is high, since summary estimates from meta-analyses are incidental to our study aims rather than directly relevant to our research questions.”

In addition, we have replaced the follow paragraph in the Results section,

“The summary P/O ratio from the meta-analysis of 17 studies conducted in the USA was 1.065 with a 95% confidence interval of 0.763 - 1.486, as presented in Fig 3-A. The summary P/O ratios (95% confidence intervals) from the meta-analyses of studies conducted in Europe were 1.632 (1.346 - 1.978) for the UK, 1.846 (1.246 - 2.737) for Northern Europe, 1.399 (0.920 - 2.128) for Western Europe, and 2.186 (1.868 - 2.555) for Southern Europe, as illustrated in Fig 3-B.”

with

- Line 310-311: “As presented in Figs 3-A and 3-B, the P/O ratios that were obtained showed high heterogeneity, with an *I*^2^ statistic greater than 80% in all analyses.”

Similarly, we have replaced

“The meta-analysis results of the *c* statistic of the Framingham Wilson CHD risk rule are presented in Fig 4. The summary *c* statistic and 95% confidence interval from the meta-analysis of seven studies conducted in the USA were 0.695 and 0.643 - 0.742. The summary *c* statistic (95% confidence intervals) from the meta-analyses of studies conducted in Europe were 0.694 (0.667 - 0.720) for the UK, 0.703 (0.621-0.773) for Northern Europe, 0.691 (0.663 - 0.718) for Western Europe, and 0.658 (0.603 - 0.709) for Southern Europe.”

with

- Line 333-340: “Forest plots of the *c* statistic of the Framingham Wilson CHD risk rule are presented in Fig 4. Studies conducted in the USA, Northern Europe, and Southern Europe had high heterogeneity with a corresponding *I*^2^ statistic of 96.98%, 96.50%, and 79.06%, respectively. The summary *c* statistic (95% confidence interval) from the meta-analysis of three studies conducted in the UK was 0.699 (0.680 - 0.718) with an *I*^2^ statistic of 30.33%. The summary *c* statistic (95% confidence intervals) from the meta-analyses of five Western European studies was 0.692 (0.660 - 0.722) with an *I*^2^ statistic of 49.00%.”

Further, we have added the following text to the Summary of Findings subsection of the Discussion:

- Line 406-415: “The heterogeneity between studies suggests that, even when many previous validations have been conducted in a particular geographic location, there is still potential to learn something new due to differences in populations, changes in predictor and outcome definitions, and temporal evolution of clinical practice (71). In particular, since the Framingham Heart Study defined these outcomes several decades ago, standard practices for diagnosing CHD have evolved greatly (72-74). Therefore, we do not argue that the validation studies were wasteful in themselves. Rather, we encourage authors of validation studies to maximise the value of their study: by publishing comprehensive validation statistics and by publishing an updated (“recalibrated”) rule when indicated.”

and the following text to the Strengths and Limitations subsection:

- Line 485-488: “We only considered overall calibration (P/O ratio) and discrimination (*c* statistic) in particular; good validation should consider three or more dimensions (89, 90). However, focussing on two commonly reported aspects of validation did not prevent us from demonstrating research inefficiencies in this literature. Lastly, some…”

2. ***Minor point: You state that you have corrected the misspelling in Fig.1 (measure instead of meausre). I suggest that you check again.***

We corrected all misspells in Fig 1.

3. ***Remark: Regarding the Simmons 2008 (56) and the Sivapalaratnam (2010 Oct) study (Table 3), I want to mention that the readers of the paper will not be able to guess the details.***

We added the following sentences to the main text and revised the legend of Table 3 to explain two studies, Vaidya 2007 (53) and Simmons 2008 (54), were ineligible for the meta-analysis of the P/O ratio. We also recognise that P/O ratios can potentially be obtained using methods other than those used in our study. Therefore, we discussed it in the Strengths and Limitations section.

- Line 358-361: “All 23 studies were eligible to be included in the meta-analysis of c static. However, only 21 of 23 studies were eligible for the meta-analysis of the P/O ratio; two studies, Vaidya 2007 (53) and Simmons 2008 (54), were ineligible because they were conducted using a subset of data from the same cohort used by another eligible study.”
- Line 488-494: “Also, we followed the approaches of Debray et al (47), as described in S2 Appendix, for extracting P/O ratios; arguably, a systematic reviewer with sufficient software and mathematical knowledge could also estimate P/O ratios from published calibration plots, albeit inexactly; this would increase the number of external validation studies of the Framingham Wilson CHD risk rule that contributed to understanding the performance in eligible meta-analyses of P/O ratio and c statistic from 10 of 23 (43.5%) to 11 (47.8%), but not change our overall conclusions.”
