# Supplementary material for: Research inefficiencies in external validation studies of the Framingham Wilson coronary heart disease risk rule: A systematic review
Source: PLoS One. 2024 Sep 13;19(9):e0310321. doi: 10.1371/journal.pone.0310321 (PMC12140082; doi:10.1371/journal.pone.0310321)
Supplement: Attachment — Submitted filename: Response to Reviewers.docx [file pone.0310321.s013.docx]

PONE-D-22-18030R4

Research inefficiencies in external validation studies of the Framingham Wilson coronary heart disease risk rule: A systematic review

Additional Editor Comments:

**Please complete revision as suggested by Reviewer 6:**

1. ***Figures 3A and 3B are difficult to understand. Providing a detailed legend would help the reader interpret the figures more easily. Additionally, the x and y axis labels are missing.***

**Response**:

We appreciate the reviewer’s comment. Accordingly, we revised the legend and explained the components of Figures 3A and 3B. Also, we added the x-axis label to indicate the figures presented the Predicted/Observed (P/O) ratio. We removed numbering on the y-axis to make it clear that each P/O ratio in forest plots corresponds to the study presented in the same row. For consistency, we made similar changes to Fig 4 and its legend.

Line 332–347 : “**Study**: the last name of the study's first author (publication year and month). Studies with an explicit aim to externally validate the Framingham Wilson coronary heart disease rule are presented in bold characters; **Country**: the country where the study was conducted; **Cohort**: the specific cohort where the Framingham Wilson coronary heart disease rule was applied; **Predicted, n/N**: the number of participants with the predicted outcome (n) and the total number of participants (N); **Observed, n/N**: the number of participants with the observed outcome (n) and the total number of participants (N).”

2. ***The authors could also better address the large heterogeneity in the dataset.***

Regarding the large heterogeneity noted in the results of the included studies, we revised the manuscript to make the following points clear to the readers: (1) heterogeneity, sometimes to a large degree, is often assumed to be present in external validation studies of clinical prediction rules (CPRs) and (2) we did not aim to summarise the performance of the Framingham Wilson CHD risk rule (and meta-analyses were not directly relevant to our research questions), we did not explore the heterogeneity extensively.

Line 484-486:“Heterogeneity is often expected among external validation studies of clinical prediction rules. We did not explore the source of heterogeneity in our data because evaluating the predictive performance of the Framingham Wilson CHD risk rule was not one of our aims.”

3. ***The current language of the article is too harsh and requires major revisions for language and content.***

We would like to emphasize that it was not our intention to use harsh language, nor was it to be disrespectful to researchers externally validating clinical prediction rules. On the contrary, we admire and appreciate their work, which provides essential evidence that is a prerequisite for implementing high-quality clinical prediction rules. Our hope was to point out areas where making certain changes could enhance the value of their work. Therefore, we tried our best to identify and revise the parts of the manuscript that might have the potential to be unintentionally interpreted as too harsh criticism.

Abstract

(line 43): changed “Most… failed to” to “Many… did not”.

Introduction

(line 71): changed “wasted” to “lost”; changed “promote the proliferation” to “inadvertently contribute to creating”

(line 103-104) changed “According to the TRIPOD statement ... should be reported in all” to

“The TRIPOD statement ... recommends that authors report”

Discussion

(line 422): changed ‘fail to’ to ‘could not’

(line 427) changed ‘research waste and inefficiencies’ to ‘research inefficiencies’

(line 435) inserted ‘they still created valuable knowledge’
